# Bioprospecting *Staphylococcus* Phages with Therapeutic and Bio-Control Potential

**DOI:** 10.3390/v12020133

**Published:** 2020-01-23

**Authors:** Joseph M. Ochieng’ Oduor, Ermir Kadija, Atunga Nyachieo, Marianne W. Mureithi, Mikael Skurnik

**Affiliations:** 1 KAVI—Institute of Clinical Research, College of Health Sciences, University of Nairobi, P.O. Box, Nairobi 19676–00202, Kenya; marianne@uonbi.ac.ke; 2 Department of Bacteriology and Immunology, Medicum, Human Microbiome Research Program, Faculty of Medicine, University of Helsinki, 00014 UH Helsinki, Finland; 3 Department of Biology-Chemistry, University of Shkodra “Luigj Gurakuqi”, 4001 Shkodra, Albania; ermirkadija@gmail.com; 4 Department of Reproductive Health & Biology, Phage Biology Section, Institute of Primate Research, P.O. Box, Karen-Nairobi 24481-00502, Kenya; anyachieo@yahoo.com; 5 Division of Clinical Microbiology, Helsinki University Hospital, HUSLAB, 00029 HUS Helsinki, Finland

**Keywords:** MRSA, Kayvirus, bacteriophage, genome, proteome, stability

## Abstract

Emergence of antibiotic-resistant bacteria is a serious threat to the public health. This is also true for *Staphylococcus aureus* and other staphylococci. *Staphylococcus* phages Stab20, Stab21, Stab22, and Stab23, were isolated in Albania. Based on genomic and phylogenetic analysis, they were classified to genus *Kayvirus* of the subfamily *Twortvirinae.* In this work, we describe the in-depth characterization of the phages that electron microscopy confirmed to be myoviruses. These phages showed tolerance to pH range of 5.4 to 9.4, to maximum UV radiation energy of 25 µJ/cm^2^, to temperatures up to 45 °C, and to ethanol concentrations up to 25%, and complete resistance to chloroform. The adsorption rate constants of the phages ranged between 1.0 × 10^−9^ mL/min and 4.7 × 10^−9^ mL/min, and the burst size was from 42 to 130 plaque-forming units. The phages Stab20, 21, 22, and 23, originally isolated using *Staphylococcus xylosus* as a host, demonstrated varied host ranges among different *Staphylococcus* strains suggesting that they could be included in cocktail formulations for therapeutic or bio-control purpose. Phage particle proteomes, consisting on average of ca 60–70 gene products, revealed, in addition to straight-forward structural proteins, also the presence of enzymes such DNA polymerase, helicases, recombinases, exonucleases, and RNA ligase polymer. They are likely to be injected into the bacteria along with the genomic DNA to take over the host metabolism as soon as possible after infection.

## 1. Introduction

The WHO considers the rapid emergency of Multi-Drug-Resistant Bacteria (MDRB) as a threat to global public health [1]. It is also predicted that MDRB-related infections will cause about 10 million deaths annually by 2050 [2]. In addition, MDRB are predicted to become a major burden to the global economy as the World Bank estimates that by the year 2050 the bacteria might result in an annual gross domestic production (GDP) loss worth 120 trillion US dollars [3]. The predicted reduction in GDP will be due to increased morbidity and mortality among the workforce, and livestock loss caused by infectious MDRB pathogens such as *Staphylococcus aureus*. *S. aureus* is a pathogen that is an etiological agent of bacteremia, soft skin and tissue infections, osteomyelitis, endocarditis, meningitis, haematogenous organ infections, food poisoning, and toxic shock syndrome in humans [4,5]. In the livestock and pet industry, staphylococci are associated with arthritis and comb necrosis in chicken; mastitis in dairy cattle, extraocular infections in horses, ovine staphylococcal dermatitis in sheep and exudative epidermitis in pigs [6,7]. These infections are often expensive to treat and might results to loss of lives or livestock if the etiological agent is an MDRB that is impossible to completely eradicate from the environment. In nature, the bacteria have been exposed to all kinds of antimicrobial agents for millions of years, either as compounds secreted by competing microbes, or as predators such as bacteriophages [8]. Bacteria, including *S. aureus*, during evolution have responded by inventing resistance mechanisms towards antimicrobial agents. The antibiotic resistance arsenal of *S. aureus* includes (i) antibiotic-resistant genes acquired by horizontal gene transfer from other bacteria, (ii) altered drug target sites due to point mutations, and (iii) active efflux pumps that when activated prevent the intracellular entry of the antimicrobial agents [9,10]. Currently, among patient or livestock isolates of *S. aureus*, *S. epidermidis* and *S. haemolyticus* there are strains that are resistant to virtually all available antibiotics [11,12]. On the other hand, at the same time, there is a huge dearth in the research and development of novel antibacterial molecules. Many pharmaceutical companies have withdrawn from antibiotic production since it is considered as a less lucrative venture [13]. In addition, rapid resistance development against novel antibiotics and tough governmental regulations overseeing drug production are other discouraging factors [14,15].

Bacteriophages (phages) are prokaryotic viruses that replicate in bacteria and exist wherever the host bacteria are found. Phages are considered as a promising alternative against the rapidly emerging MDRB such as methicillin-resistant *S. aureus* (MRSA), *S. haemolyticus* (MRSH), and *S. epidermidis* (MRSE) [12,13,14,15,16]. In general, bacteriophages form the most numerous lifeform on earth with an estimated number reaching 10^30–31^ [17]. Application of phages as therapeutic or biocontrol agents has been practiced for almost a century in Georgia and Russia [18]. There are no reports from these countries describing adverse effects or inefficiency when used for either phage therapy or bioremediation [19,20]. Recent animal experiments have proved the efficacy and safety of phages as therapeutic agents [21,22]. In addition, some phage products have been approved for human use by the US Food and Drug Administration department (US-FDA) [20].

Phages and bacteria have co-evolved for billions of years under constant arms’ race [23]. As a result, bacteria have developed complex defense mechanisms against phages. These mechanisms include (i) swapping of receptors to prevent phage attachment on the host surface, (ii) abortive replication cycle or “suicide death” to save bordering bacteria, (iii) restriction modification systems to degrade phage DNA and prevent its replication, and (iv) adaptive immune system referred to as clustered regularly interspaced short palindromic repeats (CRISPR) [24]. Furthermore, some bacteria use chemical defense to get rid of predator phages [25]. As a countermeasure, phages have adapted various means to cross the bacterial defense lines including (i) possession of several receptor binding proteins, (ii) anti-CRISPR genes, and (iii) using modified nucleotides in their DNA [26,27,28,29]. In practice, this makes it possible to isolate new phages against almost any bacterial strain that might be otherwise difficult to control.

Application of phages for therapeutic purposes requires continuous isolation of phages with new host specificities to meet the emerging bacterial pathogens that evolve under the never-ending arms race between phages and bacteria. Therefore, in this work, we have characterized and present detailed analyses of four *Staphylococcus* phages that belong to genus *Kayvirus* of the subfamily *Twortvirinae*. Two of the phages demonstrated a relatively broad host range while the other two infected just a few *Staphylococcus* strains.

## 2. Materials and Methods

### 2.1. Bacterial Strains, Phages and Media

The bacterial strains used in the study are listed in Table S1. They include isolates originating from the Hospital District of Helsinki and Uusimaa Laboratories (HUSLAB, Vantaa, Finland), Finland, and pig isolates [30]. The *Staphylococcus xylosus* strain DD-34 that was used as a host strain when isolating the phages [31] is a natural sausage fermenter isolated from dried sausage [32]. The incubations of staphylococci and phage isolations were performed at 37 °C using Lysogeny Broth (LB) [33]. The bacteriological agar (Lab M limited, Lancashire, UK) content was 0.3% (*w*/*v*) for soft agar and 1.5% (*w*/*v*) for solid medium.

### 2.2. Phage Isolation and Purification

The isolation of phages Stab20, Stab21, Stab22, and Stab23 from Albanian sewage and river water samples using *S. xylosus* strain DD-34 as host bacteria was described in [31]. The accession numbers of the DNA sequences of the genomes are LR215718, LR215719, LR215720 and LR215721, respectively. The phages were propagated as described previously [21]. Semi-confluent solid media propagation method was used to prepare high titer stocks. Briefly, 200 µL of 90 min cultured host bacteria at OD_600_ 1–1.5, and 50 µL of appropriately diluted phage suspension were added to 3.0 mL LB overlay medium (0.3% agar) supplemented with 5 mM CaCl_2_. The phages were recovered from the overlay agar as described [34]. The phages were resuspended into SM-buffer with 8% sucrose after concentrating and washing thrice, and stored at +4 °C.

### 2.3. Transmission Electron Microscopy (TEM)

Phage suspensions (>10^7^ pfu/mL) were centrifuged 90 min at full speed in Eppendorf microfuge and the phages resuspended into 200 µL of 0.1 M ammonium acetate. Three µL of phage suspension was pipetted on carbon-coated copper grids, and after 60 sec adsorption the grids were stained with 2% uranyl acetate (pH 7.4) for 15 s. The grids were then observed using the JEOL JEM-1400 TEM (Jeol Ltd., Tokyo, Japan) fitted with a bottom-mounted Gatan Orius SC 1000B camera (Gatan Inc., Pleasanton, CA, USA). The specimens were inspected at 80 KV beam voltage with 80,000× and 150,000× magnifications at Electron Microscopy Unit (Institute of Biotechnology, University of Helsinki-Finland, Helsinki, Finland). The dimensions of five to ten virions were determined and the measurements used to calculate the averages and standard errors.

### 2.4. Experiments on Physical and Chemical Properties

Phage suspensions with titers 10^7^ to 10^10^ pfu/mL were prepared. Thermal stability was established by incubating phages at 35, 40, 45, 50, 55 and 60 °C in phosphate buffered solution (pH 7.4) for one hour after which the tubes were cooled on ice for 30 min. pH-stability experiments were performed by incubating phage suspensions at pH 1.4, 3.4, 5.4, 7.4, 9.4, 11.4 and 12.9, at 37 °C, for one hour. Ultra-violet (U.V) stability was evaluated by exposing 200 µL of lysate in microtiter plate wells to U.V energy (0, 25, 50, 75, 100, 125 and 150 µJ/cm^2^) using UVP CL-1000 Cross-Linker [35]. The surviving phage particles after these stability experiments were determined by double-layer agar method. Phage suspensions were also subjected to 20, 25, 30, 35, 40 and 50% (vol/vol) ethanol and 100% chloroform. Spot assay of serially diluted samples was carried out to establish phage viability.

### 2.5. Adsorption Rate Experiment

In the adsorption rate experiment log-phase host bacteria and pre-determined number of phages were used. Briefly, *S. xylosus* DD-34 bacteria were sub-cultured to 5 mL of LB and incubated at 37 °C to OD_600_ of 0.5 to 1.0. Then the bacteria were pelleted by centrifugation at 4500× *g* for 20 min and resuspended into 0.9 mL of fresh LB. Thereafter, 100 µL of phage suspension (4.5 × 10^6^ pfu) was added to experimental tube (A) and to a control tube (B) without bacteria. The two tubes were then incubated at 37 °C, 120 rpm for 10 min and sampling done at an interval of 5 min from tubes A and B. 50 µL was picked at each interval and dispensed into pre-chilled Eppendorf tubes. The samples were briefly vortexed then centrifuged at 16,100× *g* at +4 °C for 10 min. The numbers of free phages in 50 µL of the supernatant were determined by double-layer plaque assay. Plaques were counted from all the plates and number of plaques recorded at their respective time points (from 0 min to 10 min). Plaque counts from control tubes (tube B) were used as time point 0 min reference points. The values were normalized by having the average PFU of tube B representing 100%. The adsorption rate constants (*k*-values) were calculated for 5 min time points as described [36].

### 2.6. One Step Growth Curves

One step growth curve experiments were carried out as described elsewhere [37]. The plaques were counted from each plate and recorded as per corresponding time points (5, 10, 15, 20, and every 10 min until 60 min). The experiment was repeated five to ten times for each phage (Stab20, Stab21, Stab22, and Stab23) on different days.

### 2.7. SDS-PAGE and LC/MS-MS Analysis

The Stab phages were concentrated by centrifugation for 30 min at 4 °C and 5000 rpm using 100,000 kDa cutoff Vivaspin concentrator^®^ 20 [38], and further purified by a 5/40% glycerol step gradient centrifugation as described elsewhere [39]. After suspension of the pelleted phages each had a titer > 6 × 10^10^ pfu/mL. The concentrated phage stocks were diluted appropriately with RNAse free H_2_O and 20 µL mixed with 20 µL of 2× Laemmli buffer. The mixtures were heated at 100 °C for 5 min and then cooled on ice. Ten µL aliquots of the samples were analyzed by 10% SDS-PAGE. The protein bands were stained for 3 hr using the InstantBlue™ ready-to-use Coomassie protein stain. The excess stain was washed with milli-Q water and the gel image taken with the Bio-Rad XR+ gel documentation system.

Phage particle proteomes were analyzed by liquid chromatography coupled with mass spectrometry (LC-MS/MS) at the Proteomics Unit, Institute of Biotechnology, University of Helsinki. Stab phages with a titer > 6 × 10^11^ pfu/mL were used for the analysis. Prior to digestion of proteins to peptides with trypsin, the proteins in the samples were reduced with tris (2-carboxyethyl) phosphine (TCEP) and alkylated with iodoacetamide. Tryptic peptide digests were purified by C18 reversed-phase chromatography columns [40] and the mass spectrometry (MS) analysis was performed on an Orbitrap Elite Electron-Transfer Dissociation (ETD) mass spectrometer (Thermo Scientific, Waltham, MA, USA), using Xcalibur version 2.2, coupled to a Thermo Scientific nLC1000 nanoflow High Pressure Liquid Chromatography (HPLC) system. Peak extraction and subsequent protein identification were achieved using Proteome Discoverer 1.4 software (Thermo Scientific). Calibrated peak files were searched against the Stab20, Stab21, Stab22 and Stab23, and *Staphylococcus aureus* subsp. aureus ST398 proteins (ASM188707v1, NCBI) by a SEQUEST search engine. Error tolerances on the precursor and fragment ions were ± 15 ppm and ± 0.8 Da, respectively. For peptide identification, a stringent cut-off (0.05 false discovery rate or 5%) was used.

### 2.8. Host Range Testing

The host ranges of the Stab phages were determined using 100 *Staphylococcus* strains representing *S. aureus, S. epidermidis, S. saprophyticus,* and *S. haemolyticus* (Table S1). Strains to be screened for susceptibility were grown in LB medium for 90 min at 37 °C, 120 rpm to an OD_600_ of 1–1.5. Warm 3.0 mL soft agar (0.3%) LB cooled to 50 °C was mixed with 0.15 mL of the bacterial suspension, poured evenly on pre-warmed 1.5% LB agar plates and allowed to solidify. The Stab phage stocks were serially diluted and 4.0 µL of each dilution was administered onto the solidified soft agar. The plates were incubated at overnight and the strains giving a positive spot assay result were tested for relative efficiency of plating (REOP). Briefly, similar aliquots of 10^−5^ to 10^−6^ dilution of each Stab phage were parallelly plated in the soft agar with the *S. xylosus* DD-34 indicator bacteria and the test strain. After overnight incubation at 37 °C, REOPs were calculated by dividing the resulting plaque counts of the test strains with those of the indicator bacteria.

### 2.9. Statistical Analysis

The physico-chemical (thermal, pH and U.V stability), adsorption and one step growth curve experiment data were analyzed by Prism GraphPad statistical tools [41]. The comparative analysis on the stability of Stab phages was carried out using the 2way ANOVA accompanied with Bonferroni post-tests at 95% and 99% confidence intervals.

## 3. Results

Our rationale to characterize the Stab phages originally isolated using *S. xylosus* as enrichment host [31] was based on the facts that *Staphylococcus* phages generally show wide host spectra, that new *S. aureus* specific phages are not easy to encounter, and that every phage able to infect clinical *S. aureus* isolates would be a welcome addition to our collection of potential therapeutic phages.

### 3.1. Morphology

**Figure 1:**
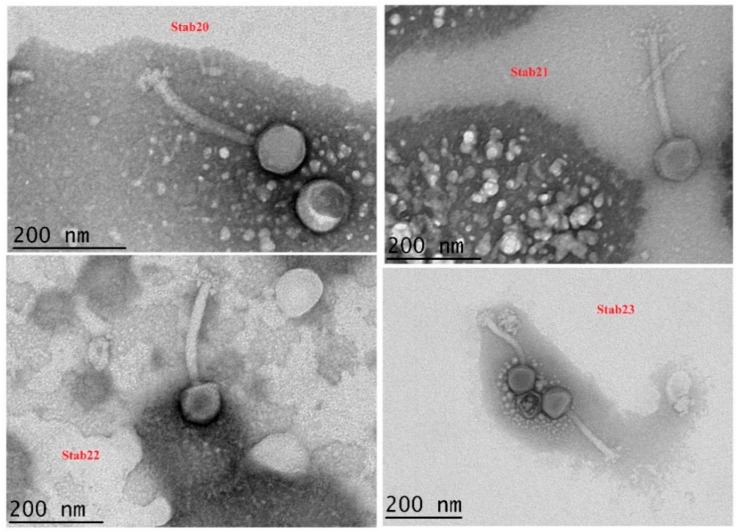
Transmission electron microscopy of phages Stab20, Stab21, Stab22, and Stab23. Uranyl acetate staining at original magnification of 25,000×.

In our previous study, genome sequence and phylogenetic analyses of phages Stab20, Stab21, Stab22, and Stab23, established that the phages are members of the genus *Kayvirus* which exclusively contains *Staphylococcus* phages [31]. Electron microscopy revealed that these phages possess icosahedral heads, long contractile tails with baseplates at the end, and tail fibers extending from the baseplates (Figure 1). The dimensions of the phage particles were close to each other but clearly distinct (Table 1). The dimensions of phages Stab20, Stab21, Stab22 and Stab23 resemble those of other genus *Kayvirus* members [36,42,43].

### 3.2. Physico-Chemical Stability

**Figure 2:**
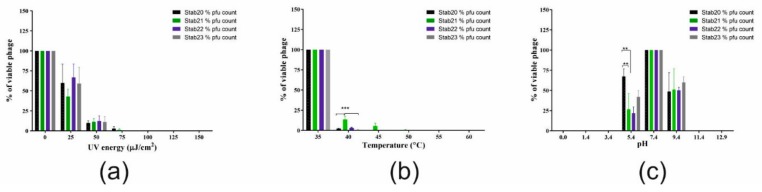
Stability of phages Stab20, Stab21, Stab22, and Stab23 under different environmental conditions. UV irradiation (**a**), Temperature (**b**) and pH (**c**).

The stability of the phages varied a little when exposed to the different environmental conditions including ultra-violet (UV) irradiation, temperature, pH and exposure to organic solvents (ethanol and chloroform). There was significant reduction of phage titer (*p* < 0.0001) when phages were exposed to 75 µJ/cm^2^ of UV-irradiation or incubated at temperatures above 45 °C. Increase in acidity or alkalinity had negative impact on the phage viability. Each phage was inactivated below pH 5.4 or above pH 9.4 (Figure 2a–c). Ethanol concentration above 25% vol/vol was enough to inactivate all four phages while they all were resistant to chloroform (Table S2). The phages were stable in 100% chloroform indicating the absence of lipids in the phage particles.

### 3.3. Adsorption Rate and Growth Curves

**Figure 3:**
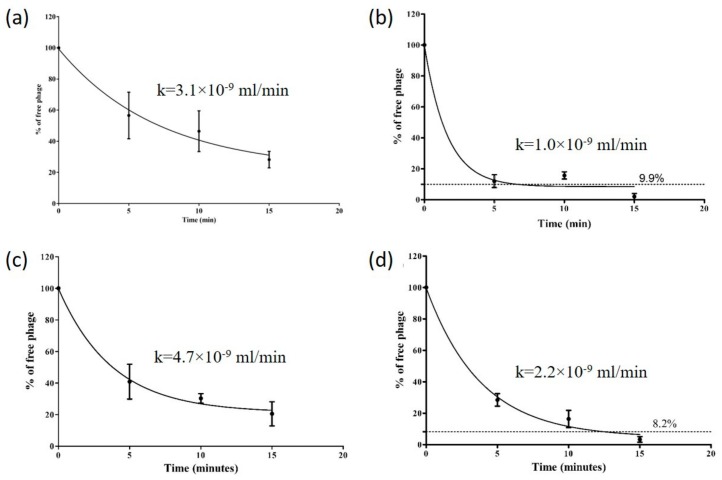
Adsorption curves and adsorption rate constants (k) of Stab20 (**a**), Stab21 (**b**), Stab22 (**c**) and Stab23 (**d**) displayed by the phages when interacting with *S. xylosus* DD-34 as host bacteria at 37 °C. The data is the average of three experiments carried out on separate days and the average bacterial titers were 7.20 × 10^8^ CFU/mL (**a**), 3.93 × 10^8^ CFU/mL (**b**), 3.83 × 10^7^ CFU/mL (**c**), and 1.20 × 10^8^ CFU/mL (**d**).

**Figure 4:**
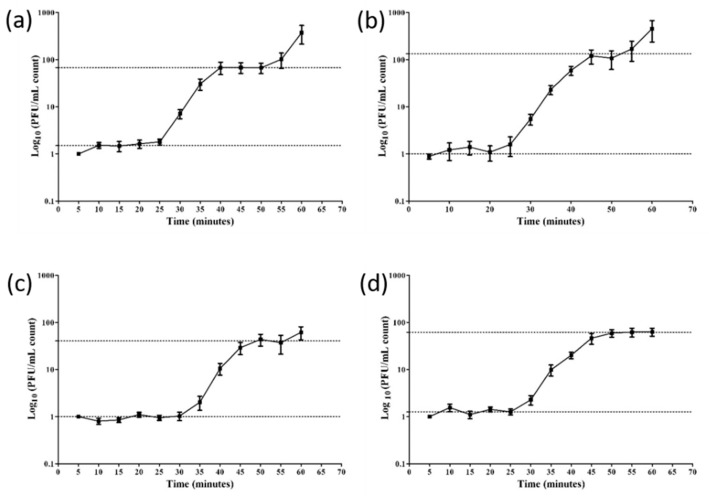
One step growth curves of phages Stab20 (**a**), Stab21 (**b**), Stab22 (**c**) and Stab23 (**d**) in *S. xylosus* DD-34 when incubated at 37 °C. The average burst sizes were 66, 130, 42 and 62 for Stab20, Stab21, Stab22, and Stab23, respectively.

The Stab phages further displayed their distinct nature through their growth curves that reflect varied adsorption rates and burst sizes. The adsorption curves represent the rates at which the phages attach to its host, also known as adsorption kinetics [44]. Of the phages, Stab21 adsorbed rapidly, ca 90% was adsorbed in 5 min while only 40, 60 and 70% of Stab20, Stab22 and Stab23, respectively, had adsorbed in the same time (Figure 3). The adsorption rate constants calculated for the 5 min time point did not differ significantly between the phages. Each phage also had its unique one step growth curve characterized by varied latent and lag phase periods. The apparent latency periods were 25–30 min. The burst sizes varied between 42 and 130 (Figure 4).

### 3.4. Analysis of Stab Phage Particle Proteomes

**Figure 5:**
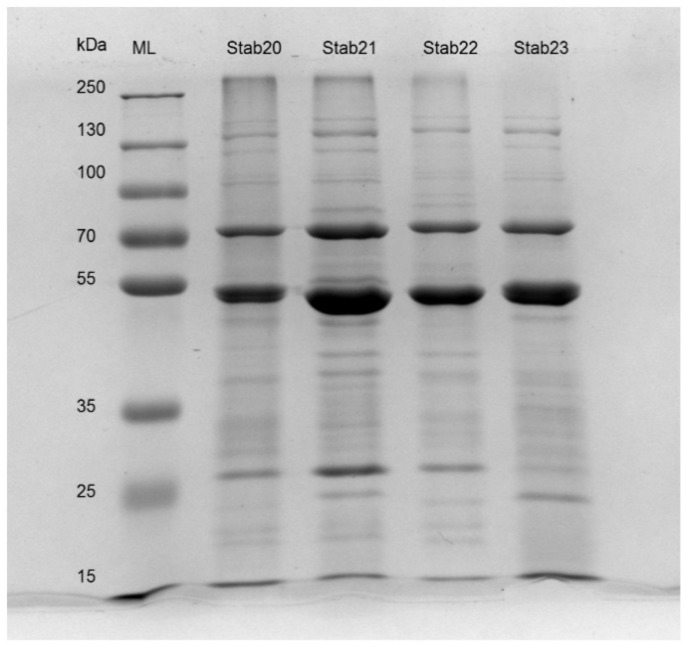
SDS-PAGE (10% acrylamide) of Stab20, Stab21, Stab22, and Stab23 showing major similarity and distinction among the phages’ structural proteins. ML—molecular ladder (broad range molecular mass marker, kDa—kiloDalton.

Previous in silico genome analysis of Stab phages predicted the presence of several structural proteins [31]. SDS-PAGE and mass spectrometry (LC-MS/MS) were used to identify the structural and phage particle-associated proteins of Stab20, Stab21, Stab22 and Stab23. The banding patterns of the phage particle proteins in SDS-PAGE analysis were very similar reflecting well the genomic similarities (Figure 5).

In SDS-PAGE the prominent bands represent the major proteins with highest copy numbers in the phages such as capsid and tail sheath proteins. Bands in the 50–55 kDa range very likely represent the predicted major capsid proteins of Stab20, Stab21, Stab22 and Stab23 that have calculated molecular masses of about 51.5 kDa. The phages also have putative 50.4 kDa capsid and scaffold proteins that would co-migrate with the major capsid protein. Indeed, close inspection of the 50–55 kDa bands of Stab20 and Stab23 in the gel reveals that they might be formed of two overlapping bands. The predicted tail tape measure proteins of the Stab phages are uniform with calculated molecular weights of 143.1 to 143.9 kDa (Figure 5) with almost identical amino acid sequences (Table S3). The tail tape measure protein determines the length of the tails, but also controls the injection of phage DNA into the host bacteria and may possess muramidase for piercing the bacterial cell-wall. The *Staphylococcus* phage K has a 1351 amino acid residue long tape measure protein, only two residues longer than that of Stab22.

**Figure 6:**
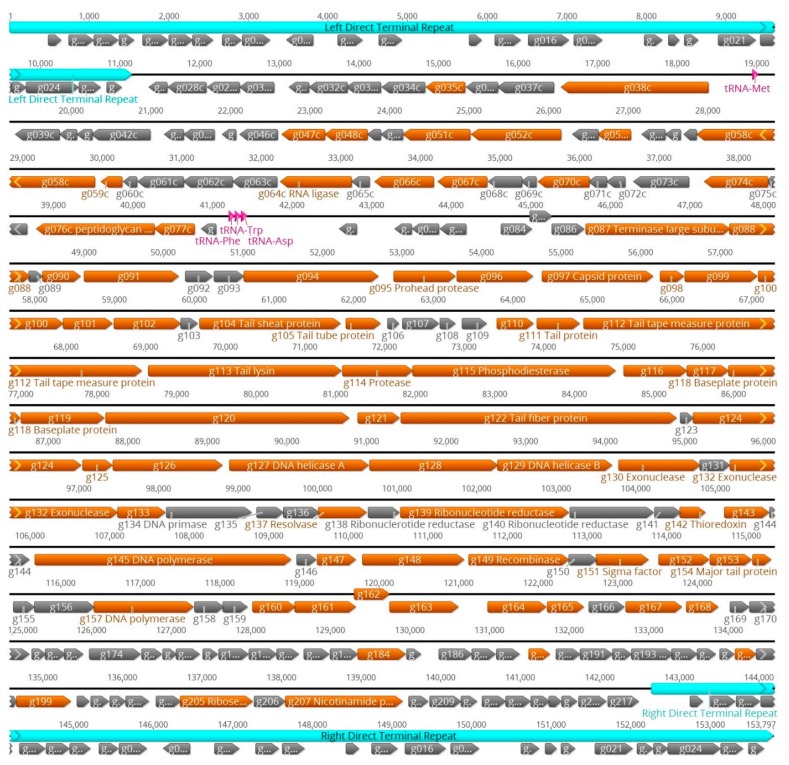
Genomic organization of phage Stab21 as a representative of the Stab phages. The sequence is shown as a black line on top of which are depicted the 11,149 bp long terminal repeat regions. The predicted genes are illustrated by arrows under the black line. The phage particle-associated gene products identified by LC-MS/MS are indicated by brown-colored arrows. The illustration was generated using Geneious 10.2 (https://www.geneious.com).

The identities of the proteins were further studied using the LC-MS/MS analysis that revealed, based on the inclusion criteria of at least two identified tryptic peptides, the presence of altogether 46 –100 phage particle-associated proteins. In Table S3, to facilitate comparisons between the phages, we have listed all the identified Stab phage proteins, with orthologues in the same rows, in parallel columns based on their decreasing molecular masses. The different total numbers of particle-associated proteins identified for the Stab phages likely reflects differences in the quantities of phage particles in the LC-MS/MS samples, with that of Stab23 being lowest. Table S3 reveals that proteins >20 kDa are most reliably identified from all phages while smaller proteins tend to be absent from some phages. Based on the results we can assign, based on experimental evidence, particle-associated/structural protein functions to 41 proteins originally annotated as hypothetical proteins (Table S4). The genomic locations of the genes encoding the phage structural and particle-associated proteins identified in the LC-MS/MS analysis are shown for phage Stab21 in Figure 6. The structural genes appear to be organized in several operon-like clusters.

In addition to proteins already annotated as structural proteins, several enzymes or proteins with other functions were also detected by LC-MS/MS (Tables S3 and S4). These included DNA polymerase (Stab21 gene *g145*, Figure 6), glycerophosphoryl diester phosphodiesterase (*g193*) likely involved in cell wall teichoic acid hydrolysis [45,46], nicotinamide phosphosribosyltransferase (*g207*) that augments nucleotide synthesis [47], ribonucleotide reductases (*g138–g140*) that are also involved in dNTP synthesis [48], endo- and exonucleases (*g130*, *g132*) playing role in host DNA degradation and provision of nucleoside 5′-monophosphate precursors for synthesis of phage DNA progeny [49]. The sigma factor (*g151*) will quide the host RNA-polymerase to start transcription from phage promoters. Also present were DNA helicases (*g127*, *g129*) that are significant in DNA synthesis and are involves in the unwinding of the double stranded DNA to create templates for replication of DNA [50], and ribose-phosphate pyrophosphokinase (*g205*) that is engaged in nucleotide salvage needed for phage replication [51]. The RNA ligase (*g064c*) was also present in the phage particles, likely involved in the repair, splicing, and editing pathways that either reseal broken RNAs or alter their primary structure [52]. The role of the PhoH-related protein (*g066c*) or PhoH-predicted ATPase is not as obvious, as they are reported to regulate phosphate uptake and metabolism under phosphate limitation [53]. GTP cyclohydrolase II has been found to be significant in riboflavin metabolism as a catalyst [54]. Furthermore, the metallophosphoesterases present in the phage particles could be involved in the catabolism of phosphodiester bonds of the host DNA to scavenge for phosphates needed in early protein biosynthesis of the phage. The lipase acylhydrolase domain protein (*g038c*) was only present in Stab21, encoded by the *g038c* gene. While no orthologous genes were present in the other Stab phages, Blastp search identifies several other *Staphylococcus* phages carrying the gene. The role of the enzyme could be in peptidoglycan/cell wall degradation thus facilitating the entry of phage DNA into the bacterial cell [55]. The proteomic data presented for the Stab phages is comparable to that of other *Staphylococcus* phages studied earlier, such as fRuSau02 for which 78 phage particle-associated proteins, including similar enzymes as for the Stab phages, were identified [30].

### 3.5. Host Range Analysis

The host ranges of the Stab phages were determined as relative efficiency of plating (REOP) when compared to the isolation host *S. xylosus* DD-34 (Table S1). The different *Staphylococcus* strains (n = 100) were isolates from both human and animal sources including MRSA (N = 46; 30 from pigs and 16 from humans), MSSA (n = 38), *S. intermedius* (n = 3), *S. epidermidis* (n = 4), *S. saprophyticus* (n = 5) and *S. haemolyticus* (n = 5). Phages Stab20 and Stab21 showed broadest host ranges. They infected 41 and 40 of the 100 strains, respectively, including both human and pig MRSA and MSSA strains and a few *S. haemolyticus, S. epidermidis*, and *S. saprophyticus* strains (Table S1). In contrast, Stab22 and Stab23 had very narrow host ranges, as both phages infected only one *S. saprophyticus* strain, and Stab 23, additionally, one *S. aureus* strain (Table S1). Interestingly, Stab20 and Stab21 propagated best in some *S. aureus* strains in which they showed REOPs of 2.4 and 2.1, respectively.

## 4. Conclusions

In this work, we have presented detailed characterization of four *Staphylococcus* phages that our previous report identified, based on genome analysis, as novel myoviruses free of any unwanted potentially harmful (toxin, antibiotic resistance, or virulence factor encoding) genes [31]. The fact that these phages can be propagated in an approved food fermenter host, *S. xylosus* strain DD-34 [32] is advantageous as such host bacteria would be safe to use to propagate phages for therapeutic or bio-control purposes [56]. A potential problem with clinical strains as propagation hosts is that they are likely to contain prophages and may produce enterotoxins, therefore, it is safer to propagate the phages in a food-quality *S. xylosus*. Even though the Stab phages had distinct host ranges, with Stab20 and Stab21 having a wide and Stab22 and Stab23 a narrow host range, their genomes proved to be closely related to other phages of therapeutic and biocontrol significance such as phage Sb-1 [31,57]. Proteomic analysis depicted uniformity among some of their structural proteins with the *Staphylococcus* phage K and other members of *Kayvirus* genus [58]. These Stab phages were stable at temperatures below 40 °C, between pH 5.4–9.4, and could resist U.V energy up to 25 µJ/cm^2^. In addition, they were resistant to chloroform but stable up to 25% ethanol.

The ancient battle for survival between bacteria and antibiotics or bacteriophages is never going to end. Continuing the use of old antibiotics and phages will not end the bacterial menace since the microbes can mutate and develop resistance to both agents [59]. However, the commercial development of novel antibiotics is both an expensive and time-consuming process often with limited scope for industrial profitability [14]. Subsequently, the search for new phages with therapeutic potential against MDR bacteria has turned into a promising alternative. Compared to discovery, development, and production of antibiotics, therapeutic phages are easy and cheap to produce [60]. In summary, these findings suggest that the Stab phages reported here may be useful candidates for phage cocktails of therapeutic or biocontrol significance. Indeed, phage Stab21 was the only phage able to infect an *S. aureus* strain recently isolated from a patient with chronic sinusitis.

## Figures and Tables

**Table 1:** Dimensions of the Stab phage particles. The measurements were taken using the TEM-camera inbuilt software at a magnification of ×15,000.

Phage	Structural Dimensions of the Stab Phages			
	Capsid Head	Tail Length	Tail Width	Baseplate Width
Stab20	84.0 ± 3.1 nm (n = 5)	163.2 ± 11.4 nm (n = 5)	21.1 ± 0.7 nm (n = 5)	48.1 ± 1.2 nm (n = 5)
Stab21	91.3 ± 0.25 nm (n = 8)	196.5 ± 3.1 nm (n = 8)	23.4 ± 0.6 nm (n = 5)	44.9 ± 1.5 nm (n = 7)
Stab22	94.3 ± 0.5 nm (n = 10)	201.6 ± 0.6 nm (n = 5)	21.3 ± 0.4 nm (n = 5)	41.8 ± 0.7 nm (n = 5)
Stab23	92.5 ± 2.6 nm (n = 10)	198.9 ± 2.9 nm (n = 9)	20.3 ± 0.3 nm (n = 9)	42.3 ± 0.8 nm (n = 5)

## Supplementary Materials

The following are available online at https://www.mdpi.com/1999-4915/12/2/133/s1, Table S1: Host range analysis of the Stab phages. Table S2: Stability of Stab phages in chloroform and ethanol. Table S3: The Stab phage particle-associated proteins identified using LC-MS/MS. Table S4: Annotation of Stab phage gene products.

## References

[1] World Health Organization Antibiotic Resistance. https://www.who.int/news-room/fact-sheets/detail/antibiotic-resistance.

[2] O’Neill J. Tackling Drug-Resistant Infections Globally: Final Report and Recommendations. https://amr-review.org/.

[3] Adeyi O.O., Baris E., Jonas O.B., Irwin A., Berthe F.C.J., Le Gall F.G., Marquez P.V., Nikolic I.A., Plante C.A., Schneidman M. (2017). Final Report Drug-Resistant Infections: A Threat to Our Economic Future.

[4] David M.Z., Daum R.S. (2010). Community-Associated Methicillin-Resistant *Staphylococcus aureus*: Epidemiology and Clinical Consequences of an Emerging Epidemic. Clin. Microbiol. Rev..

[5] Becker K., Heilmann C., Peters G. (2014). Coagulase-Negative *Staphylococci*. Clin. Microbiol. Rev..

[6] Foster A.P. (2012). Staphylococcal skin disease in livestock. Vet. Dermatol..

[7] Lowder B.V., Guinane C.M., Ben Zakour N.L., Weinert L.A., Conway-Morris A., Cartwright R.A., Simpson A.J., Rambaut A., Nübel U., Fitzgerald J.R. (2009). Recent human-to-poultry host jump, adaptation, and pandemic spread of *Staphylococcus aureus*. Proc. Natl. Acad. Sci. USA.

[8] Bhullar K., Waglechner N., Pawlowski A., Koteva K., Banks E.D., Johnston M.D., Barton H.A., Wright G.D. (2012). Antibiotic resistance is prevalent in an isolated cave microbiome. PLoS ONE.

[9] Nawrocki K.L., Crispell E.K., McBride S.M. (2014). Antimicrobial Peptide Resistance Mechanisms of Gram-Positive Bacteria. Antibiotics.

[10] Joo H.-S., Otto M. (2015). Mechanisms of resistance to antimicrobial peptides in staphylococci. Biochim. Biophys. Acta.

[11] Foster T.J. (2017). Antibiotic resistance in *Staphylococcus aureus*. Current status and future prospects. FEMS Microbiol. Rev..

[12] Czekaj T., Ciszewski M., Szewczyk E.M. (2015). *Staphylococcus haemolyticus*—An emerging threat in the twilight of the antibiotics age. Microbiol. Read. Engl..

[13] Livermore D.M., Blaser M., Carrs O., Cassell G., Fishman N., Guidos R., Levy S., Powers J., Norrby R., Tillotson G. (2011). Discovery research: The scientific challenge of finding new antibiotics. J. Antimicrob. Chemother..

[14] Simpkin V.L., Renwick M.J., Kelly R., Mossialos E. (2017). Incentivising innovation in antibiotic drug discovery and development: Progress, challenges and next steps. J. Antibiot. (Tokyo).

[15] Renwick M., Mossialos E. (2018). What are the economic barriers of antibiotic R&D and how can we overcome them?. Expert Opin. Drug Discov..

[16] Knafl D., Tobudic S., Cheng S.C., Bellamy D.R., Thalhammer F. (2017). Dalbavancin reduces biofilms of methicillin-resistant *Staphylococcus aureus* (MRSA) and methicillin-resistant *Staphylococcus epidermidis* (MRSE). Eur. J. Clin. Microbiol. Infect. Dis..

[17] Jurczak-Kurek A., Gąsior T., Nejman-Faleńczyk B., Bloch S., Dydecka A., Topka G., Necel A., Jakubowska-Deredas M., Narajczyk M., Richert M. (2016). Biodiversity of bacteriophages: Morphological and biological properties of a large group of phages isolated from urban sewage. Sci. Rep..

[18] Rohde C., Resch G., Pirnay J.-P., Blasdel B.G., Debarbieux L., Gelman D., Górski A., Hazan R., Huys I., Kakabadze E. (2018). Expert Opinion on Three Phage Therapy Related Topics: Bacterial Phage Resistance, Phage Training and Prophages in Bacterial Production Strains. Viruses.

[19] Zhvania P., Hoyle N.S., Nadareishvili L., Nizharadze D., Kutateladze M. (2017). Phage Therapy in a 16-Year-Old Boy with Netherton Syndrome. Front. Med..

[20] Moye Z.D., Woolston J., Sulakvelidze A. (2018). Bacteriophage Applications for Food Production and Processing. Viruses.

[21] Oduor J.M.O., Onkoba N., Maloba F., Nyachieo A. (2016). Experimental phage therapy against hematogenous multi-drug resistant *Staphylococcus aureus* pneumonia in mice. Afr. J. Lab. Med..

[22] Hua Y., Luo T., Yang Y., Dong D., Wang R., Wang Y., Xu M., Guo X., Hu F., He P. (2018). Phage Therapy as a Promising New Treatment for Lung Infection Caused by Carbapenem-Resistant *Acinetobacter baumannii* in Mice. Front. Microbiol..

[23] Hanlon G.W. (2007). Bacteriophages: An appraisal of their role in the treatment of bacterial infections. Int. J. Antimicrob. Agents.

[24] Oechslin F. (2018). Resistance Development to Bacteriophages Occurring during Bacteriophage Therapy. Viruses.

[25] Kronheim S., Daniel-Ivad M., Duan Z., Hwang S., Wong A.I., Mantel I., Nodwell J.R., Maxwell K.L. (2018). A chemical defence against phage infection. Nature.

[26] Scanlan P.D., Hall A.R., Lopez-Pascua L.D.C., Buckling A. (2011). Genetic basis of infectivity evolution in a bacteriophage. Mol. Ecol..

[27] Maxwell K.L. (2016). Phages Fight Back: Inactivation of the CRISPR-Cas Bacterial Immune System by Anti-CRISPR Proteins. PLOS Pathog..

[28] Tao P., Wu X., Rao V. (2018). Unexpected evolutionary benefit to phages imparted by bacterial CRISPR-Cas9. Sci. Adv..

[29] Samson J.E., Magadán A.H., Sabri M., Moineau S. (2013). Revenge of the phages: Defeating bacterial defences. Nat. Rev. Microbiol..

[30] Leskinen K., Tuomala H., Wicklund A., Horsma-Heikkinen J., Kuusela P., Skurnik M., Kiljunen S. (2017). Characterization of vB_SauM-fRuSau02, a Twort-Like Bacteriophage Isolated from a Therapeutic Phage Cocktail. Viruses.

[31] Oduor J.M.O., Kiljunen S., Kadija E., Mureithi M.W., Nyachieo A., Skurnik M. (2019). Genomic characterization of four novel Staphylococcus myoviruses. Arch. Virol..

[32] Moller J.K., Hinrichsen L.L., Andersen H.J. (1998). Formation of amino acid (L-leucine, L-phenylalanine) derived volatile flavour compounds by *Moraxella phenylpyruvica* and *Staphylococcus xylosus* in cured meat model systems. Int J Food Microbiol.

[33] Sambrook J., Russell D.W., Laboratory C.S.H. (2001). Molecular Cloning: A Laboratory Manual.

[34] Fischer S., Kittler S., Klein G., Glünder G. (2013). Microplate-Test for the Rapid Determination of Bacteriophage-Susceptibility of *Campylobacter* Isolates—Development and Validation. PLoS ONE.

[35] AJ US Analytik Jena AG. http://us.analytik-jena.com/.

[36] Vandersteegen K., Kropinski A.M., Nash J.H.E., Noben J.-P., Hermans K., Lavigne R. (2013). Romulus and Remus, Two Phage Isolates Representing a Distinct Clade within the Twortlikevirus Genus, Display Suitable Properties for Phage Therapy Applications. J. Virol..

[37] Kropinski A.M. (2018). Practical Advice on the One-Step Growth Curve. Methods Mol. Biol. Clifton N. J..

[38] Sartorius Vivaspin 20, 100,000 MWCO PES, 48pc. Sartorius. https://www.sartorius.com/shop/ww/en/usd/master-products/centrifugal-ultrafiltration-devices/vivaspin-20%2c-100%2c000-mwco-pes%2c-48pc/p/VS2042.

[39] Sambrook J., Russell D.W. (2006). Purification of Bacteriophage λ Particles by Centrifugation through a Glycerol Step Gradient. Cold Spring Harb. Protoc..

[40] Varjosalo M., Keskitalo S., Van Drogen A., Nurkkala H., Vichalkovski A., Aebersold R., Gstaiger M. (2013). The Protein Interaction Landscape of the Human CMGC Kinase Group. Cell Rep..

[41] Prism 8. https://www.graphpad.com/scientific-software/prism/.

[42] Cui Z., Guo X., Dong K., Zhang Y., Li Q., Zhu Y., Zeng L., Tang R., Li L. (2017). Safety assessment of Staphylococcus phages of the family Myoviridae based on complete genome sequences. Sci. Rep..

[43] Takemura-Uchiyama I., Uchiyama J., Kato S., Inoue T., Ujihara T., Ohara N., Daibata M., Matsuzaki S. (2013). Evaluating efficacy of bacteriophage therapy against *Staphylococcus aureus* infections using a silkworm larval infection model. FEMS Microbiol. Lett..

[44] Storms Z.J., Sauvageau D. (2015). Modeling tailed bacteriophage adsorption: Insight into mechanisms. Virology.

[45] Cornelissen A., Sadovskaya I., Vinogradov E., Blangy S., Spinelli S., Casey E., Mahony J., Noben J.-P., Dal Bello F., Cambillau C. (2016). The Baseplate of *Lactobacillus delbrueckii* Bacteriophage Ld17 Harbors a Glycerophosphodiesterase. J. Biol. Chem..

[46] Myers C.L., Ireland R.G., Garrett T.A., Brown E.D. (2015). Characterization of Wall Teichoic Acid Degradation by the Bacteriophage ϕ29 Appendage Protein GP12 Using Synthetic Substrate Analogs. J. Biol. Chem..

[47] Lee J.Y., Li Z., Miller E.S. (2017). Vibrio Phage KVP40 Encodes a Functional NAD+ Salvage Pathway. J. Bacteriol..

[48] Harrison A.O., Moore R.M., Polson S.W., Wommack K.E. (2019). Reannotation of the Ribonucleotide Reductase in a Cyanophage Reveals Life History Strategies within the Virioplankton. Front. Microbiol..

[49] Mitsunobu H., Zhu B., Lee S.-J., Tabor S., Richardson C.C. (2014). Flap endonuclease of bacteriophage T7. Bacteriophage.

[50] Lionnet T., Spiering M.M., Benkovic S.J., Bensimon D., Croquette V. (2007). Real-time observation of bacteriophage T4 gp41 helicase reveals an unwinding mechanism. Proc. Natl. Acad. Sci. USA.

[51] Nilsson E., Li K., Fridlund J., Šulčius S., Bunse C., Karlsson C.M.G., Lindh M., Lundin D., Pinhassi J., Holmfeldt K. (2019). Genomic and Seasonal Variations among Aquatic Phages Infecting the Baltic Sea Gammaproteobacterium *Rheinheimera* sp. Strain BAL341. Appl. Environ. Microbiol..

[52] Ho C.K., Shuman S. (2002). Bacteriophage T4 RNA ligase 2 (gp24.1) exemplifies a family of RNA ligases found in all phylogenetic domains. Proc. Natl. Acad. Sci. USA.

[53] Wang X., Liu J., Yu Z., Jin J., Liu X., Wang G. (2016). Novel groups and unique distribution of phage phoH genes in paddy waters in northeast China. Sci. Rep..

[54] Wagemans J., Blasdel B.G., den Bossche A.V., Uytterhoeven B., Smet J.D., Paeshuyse J., Cenens W., Aertsen A., Uetz P., Delattre A.-S. (2014). Functional elucidation of antibacterial phage ORFans targeting *Pseudomonas aeruginosa*. Cell. Microbiol..

[55] Fernández-Ruiz I., Coutinho F.H., Rodriguez-Valera F. (2018). Thousands of Novel Endolysins Discovered in Uncultured Phage Genomes. Front. Microbiol..

[56] El Haddad L., Ben Abdallah N., Plante P.-L., Dumaresq J., Katsarava R., Labrie S., Corbeil J., St-Gelais D., Moineau S. (2014). Improving the Safety of *Staphylococcus aureus* Polyvalent Phages by Their Production on a *Staphylococcus xylosus* Strain. PLoS ONE.

[57] Sergueev K.V., Filippov A.A., Farlow J., Su W., Kvachadze L., Balarjishvili N., Kutateladze M., Nikolich M.P. (2019). Correlation of Host Range Expansion of Therapeutic Bacteriophage Sb-1 with Allele State at a Hypervariable Repeat Locus. Appl. Environ. Microbiol..

[58] Ajuebor J., Buttimer C., Arroyo-Moreno S., Chanishvili N., Gabriel E.M., O’Mahony J., McAuliffe O., Neve H., Franz C., Coffey A. (2018). Comparison of *Staphylococcus* Phage K with Close Phage Relatives Commonly Employed in Phage Therapeutics. Antibiotics.

[59] Schooley R.T., Biswas B., Gill J.J., Hernandez-Morales A., Lancaster J., Lessor L., Barr J.J., Reed S.L., Rohwer F., Benler S. (2017). Development and Use of Personalized Bacteriophage-Based Therapeutic Cocktails To Treat a Patient with a Disseminated Resistant *Acinetobacter baumannii* Infection. Antimicrob. Agents Chemother..

[60] Matsuzaki S., Uchiyama J., Takemura-Uchiyama I., Daibata M. (2014). Perspective: The age of the phage. Nature.

