# Supplementary material for: Bioprospecting Staphylococcus Phages with Therapeutic and Bio-Control Potential
Source: Viruses. 2020 Jan 23;12(2):133. doi: 10.3390/v12020133 (PMC7077315; doi:10.3390/v12020133)
Supplement: Supplementary file 1 [file viruses-12-00133-s001.pdf]

Supplementary data

Bioprospecting *Staphylococcus* phages with therapeutic and bio-control potential

Joseph M. Ochieng' Oduor[1,2*] (ORCID ID: 0000-0002-0315-4051), Ermir Kadija[3] (ORCID ID: 0000-0003-1150-0011), Atunga Nyachieo[4] (ORCID ID: 0000-0002-7906-5311), Marianne W. Mureithi[1] (ORCID ID: 0000-0001-9119-3167), Mikael Skurnik[2,5*] (ORCID ID: 0000-0001-8791-9260)

[1] KAVI- Institute of Clinical Research, College of Health Sciences, University of Nairobi, Nairobi, Kenya

[2] Department of Bacteriology and Immunology, Medicum, Human Microbiome Research Program, Faculty of Medicine, University of Helsinki, Helsinki, Finland

[3] University of Shkodra "Luigj Gurakuqi", Shkodra, Albania

[4] Institute of Primate Research, Nairobi, Kenya

[5] Division of Clinical Microbiology, Helsinki University Hospital, HUSLAB, Helsinki, Finland.

*Corresponding authors: Joseph M. Ochieng' Oduor, e-mail: josemislredo@gmail.com; Mikael Skurnik, e-mail: mikael.skurnik@helsinki.fi

Supplementary tables

Table S1. Host range analysis of the Stab phages.

No.	Strains	Origin	ID	Spot assay					Average REOP			
				Stab20	Stab21	Stab22	Stab23		Stab20	Stab21	Stab22	Stab23
1	*S. aureus*	Human	5511	Neg	Neg	Neg	Neg		0.0	0.0	0.0	0.0
2	*S. aureus*	Human	5515	Pos	Pos	Neg	Neg		2.0	0.1	0.0	0.0
3	*S. aureus*	Human	5523	Neg	Neg	Neg	Neg		0.0	0.0	0.0	0.0
4	*S. aureus*	Human	5526	Neg	Neg	Neg	Neg		0.0	0.0	0.0	0.0

5	*S. aureus*	Human	5527	Neg	Neg	Neg	Neg		0.0	0.0	0.0	0.0
6	*S. aureus*	Human	5528	Neg	Neg	Neg	Neg		0.0	0.0	0.0	0.0
7	*S. aureus*	Human	5530	Neg	Neg	Neg	Neg		0.0	0.0	0.0	0.0
8	*S. aureus*	Human	5531	Pos	Pos	Neg	Neg		2.4	0.5	0.0	0.0
9	*S. aureus*	Human	5535	Neg	Pos	Neg	Neg		0.0	0.2	0.0	0.0
10	*S. aureus*	Human	5676	Neg	Neg	Neg	Neg		0.0	0.0	0.0	0.0
11	*S. aureus*	Human	5677	Pos	Neg	Neg	Neg		2.2	0.0	0.0	0.0
12	*S. aureus*	Human	5678	Neg	Neg	Neg	Neg		0.0	0.0	0.0	0.0
13	*S. aureus*	Human	5679	Pos	Pos	Neg	Neg		1.6	0.2	0.0	0.0
14	*S. aureus*	Human	5680	Pos	Neg	Neg	Neg		1.8	0.0	0.0	0.0
15	*S. aureus*	Human	5681	Neg	Neg	Neg	Neg		0.0	0.0	0.0	0.0
16	*S. aureus*	Human	5682	Pos	Pos	Neg	Neg		0.5	0.1	0.0	0.0
17	*S. aureus*	Human	5683	Neg	Neg	Neg	Neg		0.0	0.0	0.0	0.0
18	*S. aureus*	Human	5684	Pos	Pos	Neg	Neg		0.9	0.8	0.0	0.0
19	*S. aureus*	Human	5685	Pos	Pos	Neg	Neg		0.4	0.3	0.0	0.0
20	*S. aureus*	Human	5686	Pos	Pos	Neg	Neg		2.4	0.4	0.0	0.0
21	*S. aureus*	Human	5689	Neg	Neg	Neg	Neg		0.0	0.0	0.0	0.0
22	*S. aureus*	Human	5690	Pos	Pos	Neg	Neg		1.0	0.3	0.0	0.0
23	*S. aureus*	Human	5691	Neg	Neg	Neg	Neg		0.0	0.0	0.0	0.0
24	*S. aureus*	Human	5692	Neg	Neg	Neg	Neg		0.0	0.0	0.0	0.0
25	*S. aureus*	Human	5693	Pos	Pos	Neg	Neg		1.2	0.5	0.0	0.0
26	*S. aureus*	Human	5694	Pos	Pos	Neg	Neg		1.3	0.7	0.0	0.0
27	*S. aureus*	Human	5695	Neg	Neg	Neg	Neg		0.0	0.0	0.0	0.0
28	*S. aureus*	Human	5696	Pos	Pos	Neg	Neg		1.3	0.1	0.0	0.0
29	*S. aureus*	Human	5697	Neg	Neg	Neg	Neg		0.0	0.0	0.0	0.0
30	*S. aureus*	Human	5698	Neg	Neg	Neg	Neg		0.0	0.0	0.0	0.0
31	*S. aureus*	Human	5699	Neg	Neg	Neg	Neg		0.0	0.0	0.0	0.0
32	*S. aureus*	Human	5700	Neg	Neg	Neg	Neg		0.0	0.0	0.0	0.0
33	*S. aureus*	Human	5701	Pos	Pos	Neg	Neg		1.3	0.1	0.0	0.0
34	*S. aureus*	Human	5702	Pos	Pos	Neg	Neg		0.8	0.1	0.0	0.0
35	*S. aureus*	Human	5703	Neg	Neg	Neg	Neg		0.0	0.0	0.0	0.0

**36**	*S. aureus*	Human	5704	Pos	Pos	Neg	Neg		0.3	0.1	0.0	0.0
**37**	*S. aureus*	Human	5705	Neg	Neg	Neg	Neg		0.0	0.0	0.0	0.0
**38**	*S. aureus*	Human	5849	Neg	Neg	Neg	Neg		0.0	0.0	0.0	0.0
**39**	*S. aureus*	Human	5851	Neg	Neg	Neg	Neg		0.0	0.0	0.0	0.0
**40**	*S. aureus*	Human	5852	Pos	Pos	Neg	Neg		1.5	0.1	0.0	0.0
**41**	*S. aureus*	Human	5853	Pos	Pos	Neg	Pos		0.2	0.2	0.0	<0.1
**42**	*S. aureus*	Human	5854	Pos	Pos	Neg	Neg		0.6	0.4	0.0	0.0
**43**	*S. aureus*	Human	5855	Neg	Neg	Neg	Neg		0.0	0.0	0.0	0.0
**44**	*S. aureus*	Human	5856	Pos	Pos	Neg	Neg		1.7	1.1	0.0	0.0
**45**	*S. aureus*	Human	5857	Pos	Pos	Neg	Pos		0.7	0.3	0.0	0.0
**46**	*S. aureus*	Human	5858	Neg	Neg	Neg	Neg		0.0	0.0	0.0	0.0
**47**	*S. aureus*	Human	5859	Pos	Pos	Neg	Neg		2.2	2.1	0.0	0.0
**48**	*S. aureus*	Human	5860	Neg	Neg	Neg	Neg		0.0	0.0	0.0	0.0
**49**	*S. aureus*	Human	5861	Pos	Neg	Neg	Neg		0.6	0.0	0.0	0.0
**50**	*S. aureus*	Human	6209	Neg	Neg	Neg	Neg		0.0	0.0	0.0	0.0
**51**	*S. aureus*	Human	6210	Neg	Neg	Neg	Neg		0.0	0.0	0.0	0.0
**52**	*S. aureus*	Human	6211	Neg	Neg	Neg	Neg		0.0	0.0	0.0	0.0
**53**	*S. intermedius*	Human	6212	Neg	Neg	Neg	Neg		0.0	0.0	0.0	0.0
**54**	*S. intermedius*	Human	6213	Neg	Neg	Neg	Neg		0.0	0.0	0.0	0.0
**55**	*S. intermedius*	Human	6219	Neg	Neg	Neg	Neg		0.0	0.0	0.0	0.0
**56**	*S.epidermidis*	Human	6220	Pos	Pos	Neg	Neg		<0.1	<0.1	0.0	0.0
**57**	*S. epidermidis*	Human	6221	Neg	Pos	Neg	Neg		0.0	<0.1	0.0	0.0
**58**	*S. epidermidis*	Human	6222	Neg	Neg	Neg	Neg		0.0	0.0	0.0	0.0
**59**	*S. epidermidis*	Human	6223	Neg	Neg	Neg	Neg		0.0	0.0	0.0	0.0
**60**	*S. haemolyticus*	Human	6224	Pos	Neg	Neg	Neg		<0.1	0.0	0.0	0.0
**61**	*S. haemolyticus*	Human	6225	Neg	Neg	Neg	Neg		0.0	0.0	0.0	0.0
**62**	*S. haemolyticus*	Human	6226	Neg	Neg	Neg	Neg		0.0	0.0	0.0	0.0
**63**	*S. haemolyticus*	Human	6227	Neg	Neg	Neg	Neg		0.0	0.0	0.0	0.0
**64**	*S. haemolyticus*	Human	6228	Pos	Pos	Neg	Neg		<0.1	0.1	0.0	0.0
**65**	*S. saprophyticus*	Human	6229	Neg	Neg	Neg	Neg		0.0	0.0	0.0	0.0
**66**	*S. saprophyticus*	Human	6230	Neg	Neg	Neg	Neg		0.0	0.0	0.0	0.0

67	*S. saprophyticus*	Human	6231	Pos	Pos	Pos	Pos		1.8	1.3	<0.1	<0.1
68	*S. saprophyticus*	Human	6232	Neg	Neg	Neg	Neg		0.0	0.0	0.0	0.0
69	*S. saprophyticus*	Human	6233	Neg	Neg	Neg	Neg		0.0	0.0	0.0	0.0
70	*S. aureus* (MRSA)	Pig	6248	Neg	Neg	Neg	Neg		0.0	0.0	0.0	0.0
71	*S. aureus* (MSSA)	Pig	6249	Neg	Neg	Neg	Neg		0.0	0.0	0.0	0.0
72	*S. aureus* (MRSA)	Pig	6250	Neg	Neg	Neg	Neg		0.0	0.0	0.0	0.0
73	*S. aureus* (MRSA)	Pig	6251	Neg	Neg	Neg	Neg		0.0	0.0	0.0	0.0
74	*S. aureus* (MSSA)	Pig	6252	Pos	Pos	Neg	Neg		<0.1	0.1	0.0	0.0
75	*S. aureus* (MRSA)	Pig	6253	Pos	Pos	Neg	Neg		0.7	0.4	0.0	0.0
76	*S. aureus* (MRSA)	Pig	6254	Neg	Neg	Neg	Neg		0.0	0.0	0.0	0.0
77	*S. aureus* (MRSA)	Pig	6258	Neg	Neg	Neg	Neg		0.0	0.0	0.0	0.0
<u>78</u>	*S. aureus* (MRSA)	Pig	6259	Neg	Neg	Neg	Neg		0.0	0.0	0.0	0.0
79	*S. aureus* (MRSA)	Pig	6259	Neg	Neg	Neg	Neg		0.0	0.0	0.0	0.0
80	*S. aureus* (MRSA)	Pig	6260	Neg	Neg	Neg	Neg		0.0	0.0	0.0	0.0
81	*S. aureus* (MRSA)	Pig	6261	Neg	Neg	Neg	Neg		0.0	0.0	0.0	0.0
82	*S. aureus* (MRSA)	Pig	6262	Neg	Neg	Neg	Neg		0.0	0.0	0.0	0.0
83	*S. aureus* (MRSA)	Pig	6263	Neg	Neg	Neg	Neg		0.0	0.0	0.0	0.0
84	*S. aureus* (MRSA)	Pig	6264	Neg	Neg	Neg	Neg		0.0	0.0	0.0	0.0
85	*S. aureus* (MRSA)	Pig	6265	Neg	Neg	Neg	Neg		0.0	0.0	0.0	0.0
86	*S. aureus* (MRSA)	Pig	6266	Neg	Neg	Neg	Neg		0.0	0.0	0.0	0.0
87	*S. aureus* (MRSA)	Pig	6273	Pos	Pos	Neg	Neg		0.1	0.3	0.0	0.0
88	*S. aureus* (MRSA)	Pig	6274	Pos	Pos	Neg	Neg		0.9	0.4	0.0	0.0
89	*S. aureus* (MSSA)	Pig	6278	Neg	Pos	Neg	Neg		0.0	0.1	0.0	0.0
90	*S. aureus* (MRSA)	Pig	6280	Pos	Pos	Neg	Neg		0.4	0.3	0.0	0.0
91	*S. aureus* (MRSA)	Pig	6281	Pos	Pos	Neg	Neg		0.7	1.2	0.0	0.0
92	*S. aureus* (MRSA)	Pig	6283	Pos	Pos	Neg	Neg		0.8	1.0	0.0	0.0
93	*S. aureus* (MRSA)	Pig	6284	Pos	Pos	Neg	Neg		0.9	1.1	0.0	0.0
94	*S. aureus* (MRSA)	Pig	6286	Pos	Pos	Neg	Neg		0.1	0.4	0.0	0.0
95	*S. aureus* (MRSA)	Pig	6287	Neg	Pos	Neg	Neg		0.0	0.2	0.0	0.0
96	*S. aureus* (MRSA)	Pig	6288	Pos	Pos	Neg	Neg		<0.1	0.1	0.0	0.0
97	*S. aureus* (MRSA)	Pig	6295	Pos	Pos	Neg	Neg		0.4	0.5	0.0	0.0

98	*S. aureus* (MRSA)	Pig	6296	Pos	Pos	Neg	Neg		0.5	0.3	0.0	0.0
99	*S. aureus* (MRSA)	Pig	6297	Pos	Pos	Neg	Neg		0.4	0.5	0.0	0.0
100	*S. aureus* (MRSA)	Pig	6298	Pos	Pos	Neg	Neg		0.5	0.8	0.0	0.0
101	*S. xylosus*	**Sausage**	**DD-34**	Pos	Pos	Pos	Pos		1.0	1.0	1.0	1.0

REOP: relative efficiency of plating.

Table S2. Summary of the experiments on the stability of Stab phages in chloroform and ethanol. A '+' indicates resistance to the treatment; a '–' indicates sensitivity.

Treatment	Stab20	Stab21	Stab22	Stab23
**PBS**	+	+	+	+
**Chloroform**	+	+	+	+
**Ethanol 25%**	+	+	+	+
**Ethanol 30%**	-	-	-	-
**Ethanol 100%**	-	-	-	-

Table S3. The Stab phage particle associated proteins identified using LC-MS/MS of tryptic peptides. The selection criteria for the proteins were a minimum of 5% sequence coverage and identification of ≥ 2 unique peptides. The proteins given in bold-face are present in two or more phages, or identified by more than 5 tryptic peptides, and thereby considered as reliable hits

PHAGE	Stab20				Stab21				Stab22				Stab23			
Predicted function of Gp	Gene	Coverage (%)	# Unique Peptides	MW [kDa]	Gene	Coverage (%)	# Unique Peptides	MW [kDa]	Gene	Coverage (%)	# Unique Peptides	MW [kDa]	Gene	Coverage (%)	# Unique Peptides	MW [kDa]
**Tail tape-measure protein**	g114	46,97	47	143,7	g112	47,15	52	143,9	g109	51,7	51	143,7	g108	21,58	18	143,7
**Adsorption-associated tail protein**	g124	41,23	29	129,2	g122	56,25	48	129,1	g119	55,35	46	129,8	g118	11,49	9	129,7
**DNA polymerase**	g149	32,37	25	124,5	g145	34,7	30	124,5	g142	45,71	35	124,4	g142	20,85	15	122,7
**Tail morphogenetic protein**	g122	11,87	6	112,1	g120	26,27	18	116,4	g117	16,73	6	85,3	g116	3,3	3	134,9
**Glycerophosphoryl diester phosphodiesterase**	g117	35,73	14	95,9	g115	39,98	21	96,6	g112	55,92	33	96,6	g111	31,18	14	96,5
**Nicotinamide phosphosribosyltransferase**	g210	23,87	13	92,2	g207	45,1	14	56,2	g198	29,03	18	93,5	g193	15,71	5	56,2
**Tail lysin; N-acetylmuramoyl-L-alanine amidase**	g115	23,51	14	91,2	g113	31,96	18	93	g110	33,37	17	92,5	g109	25,77	14	92,4
**Ribonucleotide reductase large subunit**	g143	49,72	28	80,2	g139	62,22	35	81,2	g136	70,22	40	80,9	g136	38,2	18	80,9
**Hypothetical protein**	g061c	10,83	4	79,7	g058c	21,26	10	79,7	g053c	16,84	6	79,7	g054c	10,54	7	80,1
**Recombination exonuclease B**	g135	15,81	6	73,2	g132	9,53	3	73,5	g129	20,74	8	73,7	g129	4,38	2	73,3
**Carbohydrate binding domain-containing protein**	g126	55,94	26	72,5	g124	67,5	34	72,6	g121	34,27	12	73,2	g120	14,13	4	72,5
**Lipase acylhyrolase domain protein**					g038c	45,08	16	70,5								
**Terminase large subunit**	g087	21,16	9	70,2	g087	36,36	13	70,2	g082	43,14	18	70,4	g082	6,78	3	70,4
**DNA helicase A**	g129	35,22	13	67,2	g127	37,93	18	67,9	g124	36,05	14	67,9	g123	20,41	6	67,9
**Major tail sheath protein**	g104	50,94	19	64,4	g104	61,93	27	64,8	g099	52,96	24	64,6	g098	26,9	9	64,9
**Putative tail fiber protein**													g028c	18,6	6	64,8
**Portal protein**	g094	41,4	13	56,8	g094	55,77	23	64	g089	49,02	21	63,9	g088	22,74	9	64
**Putative replication protein**	g130	9,12	3	63,1	g128	11,71	4	63,2	g125	16,36	6	63,3				
**Endolysin; N-acetylmuramoyl-L-alanine amidase**	g077c	34,14	11	54,7	g076c	44,15	18	54,8	g072c	48,09	21	55	g072c	40,64	15	55
**DNA helicase B**	g131	14,17	6	54,6	g129	38,68	12	55,3	g126	54,32	19	55,3	g125	6,38	2	55,3
**Major capsid protein**	g097	74,08	22	51,2	g097	61,29	18	51,5	g092	61,51	19	51,5	g091	38,28	11	51,5
**Putative capsid & scaffold protein or Tail fiber protein**	g128	20,96	7	50,4	g126	33,62	10	50,4	g123	70,74	25	51	g122	29,1	11	50,7
**DNA polymerase or hypothetical protein**					g157	28,2	8	48,2	g101	16,48	5	50,9				
**Metallophosphoesterase**	g161	21,39	7	47,6					g154	36,26	9	48,3	g154	9,95	3	48,3
**DNA repair recombinase protein**	g153	46,65	20	46,7	g149	63,4	27	46,8	g146	59,28	23	46,7	g146	38,76	12	46,7
**Hypothetical protein**	g091	4,1	2	43	g091	22,98	5	43,6	g086	12,41	3	44,4				
**Hypothetical protein**	g152	28,05	7	48,3	g148	25,12	5	47,7	g145	35,09	6	44,4				
**AAA family ATPase**	g055c	26,13	7	42,5	g052c	31,69	10	43,8	g047c	51,46	17	42,7	g048c	13,39	5	43,2
**Ribonucleotide reductase small subunit or DNA primase**	g144	12,61	3	40,4					g131	7,76	2	41,7				
**Recombination exonuclease A**	g133	23,75	6	39,3	g130	23,28	4	40,2	g128	17,25	4	39,6				
**Baseplate morphogenetic protein or putative tail protein**	g121	15,52	4	39,2	g119	15,47	4	39,3	g116	19,48	5	39,2	g115	15,47	3	39,2
**Hypothetical protein**	g096	12,54	3	36,1	g096	17,28	4	36,7								
**Ribose-phosphate pyrophosphokinase**	g209	29,8	7	34,9	g205	24,68	6	35,9	g197	40,07	8	35,2				
**RNA ligase**	g066c	6,71	2	34,7	g064c	34,45	7	35,2								
**Protease or Peptidoglycan hydrolase**	g116	10,17	3	34,5	g114	13,85	4	34,7	g111	11,15	4	34,8	g110	10,47	2	34,7
**Hypothetical protein**					g088	32,89	8	34,2	g083	26,38	5	34,6				
**Hypothetical protein**	g099	25,17	4	34,1	g099	19,21	4	34,1	g094	25,17	4	34,1	g093	18,21	3	34,1
**Capsid protein or Hypothetical protein**	g100	17,81	3	33,7	g100	43	8	33,9	g095	50,51	10	33,8				
**Hypothetical protein**	g167	19,51	4	32,3	g163	25,69	4	32,4	g160	24,31	5	32,4	g160	13,19	3	32,4
**Hypothetical protein**	g054c	10,68	3	31,8	g051c	8,62	2	32,7	g046c	29,47	6	32,1				
**Hypothetical protein**	g102	50,72	8	31,7	g102	41,73	6	31,8	g097	50,72	9	31,8	g096	48,2	9	31,8
**Hypothetical protein**					g161	13,73	2	30	g158	13,73	2	30	g158	16,86	3	30
**Hypothetical protein**	g118	16,73	3	29,3	g116	27,61	4	29,9	g113	14,18	3	29,8				
**Putative membrane protein**	g075c	42,05	12	29,3	g074c	36	10	30,7	g069c	50,76	14	29,3	g069c	9,85	3	29,3
**Hypothetical protein**	g168	19,12	3	29,3	g164	56,57	11	29,3	g161	57,37	11	29,3				
Hypothetical protein									g153	19,05	2	29,2				
**PhoH-related protein or PhoH-predicted ATPase**	g067c	15,04	3	28,5	g066c	54,66	12	28,7	g061c	50,2	13	28,7	g061c	19,43	3	28,9
**Prohead protease**	g095	60,16	12	27,3	g095	61,39	16	28,8	g090	62,84	12	28,8	g089	21,46	3	28,9
**Hypothetical protein**	g147	13,64	2	23,6	g143	28,1	5	28,8	g140	28,1	5	28,8				
**Hypothetical protein**					g167	27,31	4	27,9	g164	12,61	2	28				
**Serine/threonine protein phosphatase**	g041c	9,87	2	27,2					g036c	5,08	2	27,9				
**RNA polymerase sigma factor**					g151	15,42	2	27,3	g148	20,7	3	27,3				
**Baseplate wedge subunit or Putative baseplate protein**	g120	9,4	1	26,6	g118	20,34	2	26,8	g115	11,86	2	26,8				
Hypothetical protein					g174	11,5	3	26,5								
**Hypothetical protein**					g070c	11,01	3	26,3	g065c	14,54	3	26,3				
Transglycosylase					g073c	13,22	2	26,3								
Hypothetical protein									g187	20,52	4	25,9				
**Hypothetical protein**	g195	41,18	5	24,9	g199	22,12	4	25,7	g194	22,57	3	25,8				
**Hypothetical protein or Anti-sigma factor**	g137	45,07	6	24,9	g133	42,92	8	24,7	g130	37,26	5	25	g130	26,42	3	24,8
**Hypothetical protein**	g187	14,5	2	23,4	g184	24,52	4	24,2	g180	25	4	24,2				
**Hypothetical protein**	g101	15,53	4	23,8	g101	28,99	8	23,8	g096	20,29	6	23,8	g149	21,76	3	23,9
**Resolvase**	g141	30,2	4	23,6	g137	52,45	8	23,9	g134	47,55	7	23,9	g134	13,73	2	24
**Hypothetical protein**	g156	62,38	9	23,2	g152	69,91	11	23,9	g149	70,37	12	24				
**Hypothetical protein**					g067c	27,75	2	23,5	g062c	36,41	4	23,3				
**Nucleoside 2-deoxyribosyltransferase**									g057c	34,83	5	22,5				
**Hypothetical protein**	g164	20,22	3	20,7	g160	21,2	3	21,5	g157	12,5	2	21,5				
**Holin**	g078c	20,36	4	18,1	g077c	18,28	4	20,4	g073c	17,62	4	21,1				
**Tail morphogenetic protein**					g111	49,49	5	23,1								
**HNH endonuclease**	g189	18,09	4	21,5												
Hypothetical protein					g170	14,14	2	22,3								
Metallophosphatase									g190	15,17	2	20,8				
**Hypothetical protein**	g051c	17,61	2	20,5	g048c	39,66	5	20,9	g043c	31,84	4	20,9				
**Hypothetical protein**					g047c	23,2	3	20,5	g042c	16,2	2	20,8				
**Hypothetical protein**									g002	61,24	8	20				
Hypothetical protein	g216	20,12	2	20,3												
**Hypothetical protein**	g119	68,97	8	19,9	g117	70,86	8	20,1	g114	65,14	7	20	g113	32,57	3	20
**Hypothetical protein or GTP cyclohydrase II**	g038c	25,93	3	19,5	g035c	30,12	3	20	g035c	38,89	6	19,5	g035c	13,94	2	19,9
**Hypothetical or Structural or Baseplate morphogenetic protein**	g123	59,54	8	19,2	g121	60,67	9	19,8	g118	52,25	8	19,9	g117	60,67	9	19,9
**Hypothetical protein**	g169	26,32	3	17,8	g165	24,39	3	19,3	g162	35,98	5	19,2	g162	24,39	3	19,3
**Hypothetical protein**									g207	58,18	8	19,5				
Hypothetical protein									g163	19,02	2	19,3				
**Hypothetical protein**									g019	30,38	5	19				
**Hypothetical protein**	g112	34,87	4	18,1	g110	33,33	4	19	g107	34,81	4	18,9	g106	27,22	3	18,9
**Hypothetical protein**					g147	23,75	4	18,9	g144	13,75	2	19	g144	16,25	2	18,9
**Hypothetical protein**	g090	17,61	3	18,5	g090	14,47	2	18,5	g085	23,27	4	18,5	g084	17,61	2	18,5
**Tail protein or Ig-like domain containing protein**	g157	33,53	8	18,2	g153	92,57	12	18,4	g150	44,25	6	18,4				
Hypothetical protein									g032c	13,82	2	18,5				
**Hypothetical protein**	g140	12	2	17					g133	22,78	4	18,3				
**Putative membrane protein**	g172	18,94	2	15,4	g168	20,13	4	18,1	g165	33,1	7	16,9	g165	27,81	4	17,5
**Tail tube protein**	g105	44,37	5	15,9	g105	43,75	5	16,2	g100	45,65	5	15,4	g099	52,78	6	16,1
**Hypothetical protein**					g054c	16,45	2	17,6					g050c	18,12	2	16
Ribonuclease H	g069c	13,48	2	15,8												
**Hypothetical protein**					g162	25,5	2	17,4	g159	31,54	5	17,5				
Membrane protein									g079	17,61	3	16,6				
Hypothetical protein									g059c	38,46	2	16,3				
Hypothetical protein									g189	42,07	3	16,2				
Hypothetical protein									g049c	21,01	2	15,9				
**Hypothetical protein**	g127	50	6	14,6	g125	40,85	4	17	g122	69,42	5	14,4				
Hypothetical protein					g036c	21,05	3	16,3								
Hypothetical protein					g180	30,25	2	13,4								
**Hypothetical protein**									g206	38,79	5	13,6				
Hypothetical protein									g147	21,37	2	13,4				
**Hypothetical protein**	g111	13,59	2	12,2					g106	12,96	2	12,9				
**Thioredoxin-like protein**	g146	15,45	2	12,5	g142	43,75	5	12,8	g139	58,04	5	12,8				
**Putative membrane protein**	g076c	17,65	2	12,2					g070c	13,21	2	12,8				
Nucleoside triphosphate pyrophosphohydrolase					g055c	20,95	2	12								
Hypothetical protein									g188	25,49	2	11,8				
**Hypothetical protein**	g194	48,89	4	10,4					g186	33	2	11,5				
**Hypothetical protein**	g098	15,31	2	11,2	g098	14,56	2	11,9	g093	15,79	2	10,9				
**Hypothetical or Terminal repeat encoded protein U (TreU)**					g189	20,41	2	11,2	g029	26,67	2	10,9				
**Hypothetical protein**					g197	62,07	6	10,2								
**Hypothetical protein**	g062c	28,74	2	10,1	g059c	43,68	4	10,1	g054c	65,52	8	10,2	g055c	13,83	2	10,9
Hypothetical protein					g041c	32,39	2	8,5								
Hypothetical protein									g067c	16	2	9,2				
Hypothetical protein									g210	25,33	2	8,6				
**Ig-like domain containing or Major tail protein**	g158	78,67	4	7,8	g154	81,58	6	7,9	g151	86,08	3	8,3				
Hypothetical protein									g182	19,35	2	7,6				

Table S4. Annotation of Stab phage gene products. The LC-MS/MS identified gene products are highlighted in grey

Stab20

Gp	Genomic location	Predicted function	AA	MW	Best hit (acc no)	value (query coverage)	phage with similar gene
Gp001	484..654	putative membrane protein	56	6298	AUV57100.1	1e-31 (100 %)	Staphylococcus phage vB_SauM_LM12
Gp002	670..963	hypothetical protein	97	11245	YP_009006863.1	6e-61 (100%)	Staphylococcus phage phiSA12
Gp003	960..1145	hypothetical protein	61	6540	YP_009099453.1	3e-33 (100%)	Staphylococcus phage P108
Gp004	1175..1399	hypothetical protein	74	8889	.....	.....	.....
Gp005	1415..1705	TreC	96	11361	AUV57038.1	9e-62 (100%)	Staphylococcus phage vB_SauM_LM12
Gp006	1705..1992	hypothetical protein	95	10858	YP_008853954.1	3e-63 (100%)	Staphylococcus phage S25-4
Gp007	1992..2285	terminal repeat-encoded protein	97	11538	YP_009099457.1	9e-62 (100%)	Staphylococcus phage P108
Gp008	2289..2537	hypothetical protein	82	9964	YP_009006868.1	2e-50 (100%)	Staphylococcus phage phiSA12
Gp009	2614..2862	hypothetical protein	82	9382	ARM69064.1	3e-24 (95%)	Staphylococcus phage vB_Sau_CG
Gp010	2859..3185	hypothetical protein*	108	13000	YP_009006870.1	2e-62 (100%)	Staphylococcus phage phiSA12
Gp011c	(3404..3742)	hypothetical protein	112	13528	YP_007002124.1	1e-71 (100%)	Staphylococcus phage GH15
Gp012	4054..4362	TreJ	102	11808	YP_007112862.1	2e-70 (100%)	Staphylococcus phage JD007
Gp013	4568..4852	hypothetical protein	94	10985	YP_009196040.1	1e-64 (100%)	Staphylococcus phage phiIPLA-RODI
Gp014	4927..5118	terminal repeat-encoded protein	63	7674	YP_007002127.1	1e-39 (100%)	Staphylococcus phage GH15
Gp015	5654..5812	hypothetical protein	52	6070	YP_008853962.1	2e-28 (100%)	Staphylococcus phage S25-4
Gp016	5978..6301	hypothetical protein	107	12453	YP_241037.1	1e-73 (100%)	Staphylococcus virus G1
Gp017	6395..6907	hypothetical protein	170	20668	VEV88440.1	6e-107 (100%)	Staphylococcus phage Stab21
Gp018	6971..7357	hypothetical protein	128	14985	VEV88378.1	1e-43 (99%)	Staphylococcus phage Stab23
Gp019	7800..8021	hypothetical protein	73	8464	YP_008853966.1	1e-47 (100%)	Staphylococcus phage S25-4
Gp020	8102..8245	hypothetical protein	47	5668	YP_008853967.1	1e-25 (93%)	Staphylococcus phage S25-4
Gp021	8302..8475	hypothetical protein	57	6783	VEV88448.1	1e-31 (100%)	Staphylococcus phage Stab21
Gp022	8555..8791	hypothetical protein	78	9008	YP_009196049.1	8e-48 (100%)	Staphylococcus phage phiIPLA-RODI
Gp023	8882..9226	hypothetical protein	114	13653	ARM69507.1	2e-75 (100%)	Staphylococcus phage vB_Sau_S24
Gp024	9293..9565	hypothetical protein	90	10843	BBC69463.1	2e-52 (96%)	Staphylococcus phage phiSA039
Gp025	9569..9733	hypothetical protein	54	6237	YP_009097941.1	6e-30 (100%)	Staphylococcus phage MCE-2014
Gp026	9737..9934	hypothetical protein	65	7628	VEV88452.1	7e-35 (100%)	Staphylococcus phage Stab21
Gp027	9939..10130	hypothetical protein	63	7314	VEV88454.1	7e-33 (95%)	Staphylococcus phage Stab21
Gp028	10111..10389	TreT	92	10605	YP_009195839.1	1e-54 (96%)	Staphylococcus phage phiIPLA-RODI
Gp029	10466..10696	TreU	76	9320	VEV89584.1	1e-46 (98%)	Staphylococcus phage Stab23
Gp030c	(10977..11267)	hypothetical protein	96	11641	YP_009097946.1	2e-63 (100%)	Staphylococcus phage MCE-2014
Gp031c	(11358..11606)	BofL	82	9992	ARM69513.1	2e-50 (100%)	Staphylococcus phage vB_Sau_S24
Gp032c	(11622..11867)	hypothetical protein	81	9612	YP 008853977.1	8e-53 (100%)	Staphylococcus phage S25-4
Gp033c	(11867..12112)	hypothetical protein	81	9979	YP_009097950.1	2e-50 (100%)	Staphylococcus phage MCE-2014
Gp034c	(12112..12303)	putative membrane protein	63	7912	YP_008853979.1	8e-38 (100%)	Staphylococcus phage S25-4
Gp035c	(12300..12785)	putative membrane protein	161	18130	YP_009097951.1	6e-110 (100%)	Staphylococcus phage MCE-2014
Gp036c	(12778..13218)	hypothetical protein	146	17215	YP_009097952.1	5e-102 (100%)	Staphylococcus phage MCE-2014
Gp037c	(13232..13774)	hypothetical protein	180	21527	YP_007002151.1	2e-127 (100%)	Staphylococcus phage GH15
Gp038c	(13786..14274)	hypothetical protein	162	19492	YP_009097954.1	1e-118 (100%)	Staphylococcus phage MCE-2014
Gp039c	(14289..14741)	hypothetical protein	150	17747	YP_009097955.1	1e-106 (100%)	Staphylococcus phage MCE-2014
Gp040c	(14758..15162)	hypothetical protein	134	16464	YP_009097956.1	2e-92 (100%)	Staphylococcus phage MCE-2014
Gp041c	(15165..15866)	Serine/threonine protein phosphatase	233	27262	YP_009097957.1	2e-172 (100%)	Staphylococcus phage MCE-2014
Gp042c	(16664..17212)	hypothetical protein	182	21975	ASZ78174.1	2e-111(100%)	Staphylococcus phage SA3
Gp043c	(17216..17434)	hypothetical protein	72	8368	YP_008853988.1	8e-46 (100%)	Staphylococcus phage S25-4
Gp044c	(17435..17629)	hypothetical protein	64	7641	YP_241059.1	4e-40 (100%)	Staphylococcus virus G1
Gp045c	(17619..18356)	hypothetical protein	245	28664	YP_008853990.1	6e-174 (100%)	Staphylococcus phage S25-4
Gp046c	(18532..18771)	hypothetical protein	79	9377	YP_007002161.1	4e-48 (100%)	Staphylococcus phage GH15
Gp047c	(18773..19162)	hypothetical protein	129	14773	YP_007002162.1	4e-85 (100%)	Staphylococcus phage GH15
Gp048c	(19256..19429)	hypothetical protein	57	6819	YP_007002163.1	2e-34 (100%)	Staphylococcus phage GH15
Gp049c	(19470..19952)	hypothetical protein	160	19000	YP_008853994.1	4e-111 (100%)	Staphylococcus phage S25-4
Gp050c	(20002..20544)	hypothetical protein	180	20462	YP_009195864.1	1e-124 (100%)	Staphylococcus phage phiIPLA-RODI
Gp051c	(20544..21074)	hypothetical protein	176	20542	YP_009195865.1	2e-125 (100%)	Staphylococcus phage phiIPLA-RODI
Gp052c	(21077..21241)	putative membrane protein	54	6153	YP_009195866.1	3e-31 (100%)	Staphylococcus phage phiIPLA-RODI
Gp053c	(21244..21531)	putative membrane protein	95	11294	YP_009195867.1	1e-59 (100%)	Staphylococcus phage phiIPLA-RODI
Gp054c	(21531..22376)	hypothetical protein	281	31774	YP_009195868.1	0.0 (100%)	Staphylococcus phage phiIPLA-RODI
Gp055c	(22390..23508)	AAA family ATPase	372	42215	YP_009099502.1	0.0 (100%)	Staphylococcus phage P108
Gp056c	(23660..24001)	hypothetical protein	108	13348	ARQ96019.1	5e-71 (100%)	Staphylococcus phage qdsa002
Gp057c	(23979..24395)	hypothetical protein	138	15993	YP_007002172.1	1e-97 (100%)	Staphylococcus phage GH15
Gp058c	(24528..24830)	NTP pyrophosphohydrolase	100	11304	YP_241074.1	1e-66 (100%)	Staphylococcus phage GH15
Gp059c	(24830..25018)	hypothetical protein	62	7321	YP_007002174.1	7e-37 (100%)	Staphylococcus phage GH15
Gp060c	(25062..25223)	hypothetical protein	53	6402	YP_007002175.1	3e-31 (100%)	Staphylococcus phage GH15
Gp061c	(25224..27275)	hypothetical protein	683	79750	YP_009195875.1	0.0 (100%)	Staphylococcus phage phiIPLA-RODI
Gp062c	(27354..27617)	hypothetical protein	87	10140	YP_008854007.1	2e-56 (100%)	Staphylococcus phage S25-4
Gp063c	(27634..27807)	hypothetical protein	57	6630	YP_009099510.1	7e-34 (100%)	Staphylococcus phage P108
Gp064c	(27814..28392)	putative membrane protein	192	21480	ASZ77976.1	1e-132 (100%)	Staphylococcus phage SA3
Gp065c	(28385..29011)	nucleoside 2-deoxyribosyltransferase	208	23648	YP_009006709.1	1e-140 (100%)	Staphylococcus phage phiSA12
Gp066c	(29001..29897)	RNA ligase	298	34738	YP_009195880.1	0.0 (99%)	Staphylococcus phage phiIPLA-RODI
Gp067c	(30193..30933)	PhoH-related protein	246	28533	YP_009195882.1	0.0 (100%)	Staphylococcus phage phiIPLA-RODI
Gp068c	(30985..31599)	hypothetical protein	204	23020	YP_008854014.1	1e-147 (100%)	Staphylococcus phage S25-4
Gp069c	(31615..32040)	ribonuclease H	141	15795	YP_007002186.1	3e-97 (100%)	Staphylococcus phage GH15
Gp070c	(32030..32221)	hypothetical protein	63	7472	YP_241086.1	1e-39 (100%)	Staphylococcus virus G1
Gp071c	(32244..32885)	hypothetical protein	213	24587	YP_009099518.1	3e-146 (100%)	Staphylococcus phage P108
Gp072c	(32875..33105)	transcriptional regulator	76	8832	YP_241088.1	3e-47 (100%)	Staphylococcus virus G1
Gp073c	(33108..33335)	hypothetical protein	75	9261	YP_009195888.1	4e-47 (100%)	Staphylococcus phage phiIPLA-RODI
Gp074c	(33444..34136)	putative transglycosylase	230	24934	YP_007002191.1	3e-169 (100%)	Staphylococcus phage GH15
Gp075c	(34334..35128)	putative membrane protein	264	29296	YP_007002192.1	0.0 (100%)	Staphylococcus phage GH15
Gp076c	(35128..35436)	putative membrane protein	102	12173	YP_007002193.1	1e-67 (100%)	Staphylococcus phage GH15
Gp077c	(35550..37037)	endolysin	495	54734	YP_009195893.1	0.0 (100%)	Staphylococcus phage phiIPLA-RODI
Gp078c	(37037..37540)	holin	167	18110	YP_009195894.1	7e-119 (100%)	Staphylococcus phage phiIPLA-RODI
Gp079c	(37625..37810)	hypothetical protein	61	7066	YP_241098.1	5e-36 (100%)	Staphylococcus virus G1
Gp080c	(39353..39571)	hypothetical protein	72	8679	YP_241099.1	1e-47 (100%)	Staphylococcus virus G1
Gp081c	(40058..40267)	hypothetical protein	69	7761	YP_007002198.1	4e-43 (100%)	Staphylococcus phage GH15
Gp082c	(40280..40612)	putative membrane protein	110	12505	YP_007002199.1	1e-69 (100%)	Staphylococcus phage GH15
Gp083c	(40625..40951)	hypothetical protein	108	13056	YP_007002200.1	6e-73 (100%)	Staphylococcus phage GH15
Gp084	41511..41777	hypothetical protein	88	10364	YP_009195900.1	2e-56 (100%)	Staphylococcus phage phiIPLA-RODI
Gp085	41755..42033	hypothetical protein	92	10579	YP_241104.1	4e-63 (100%)	Staphylococcus virus G1
Gp086	42030..42440	hypothetical protein	136	15626	YP_241105.1	9e-94 (100%)	Staphylococcus virus G1
Gp087	42455..44272	terminase large subunit	605	70243	YP_007112786.1	0.0 (100%)	Staphylococcus phage JD007
Gp088	44286..45104	hypothetical protein	272	30484	YP_009195906.1	0.0 (100%)	Staphylococcus phage phiIPLA-RODI
Gp089	45091..45264	hypothetical protein	57	6687	YP_009099539.1	9e-31 (100%)	Staphylococcus phage P108
Gp090	45261..45740	hypothetical protein	159	18540	YP_007002208.1	6e-112 (100%)	Staphylococcus phage GH15
Gp091	45782..47005	hypothetical protein	407	44868	YP_009195908.1	0.0 (100%)	Staphylococcus phage phiIPLA-RODI
Gp092	47090..47431	hypothetical protein	113	12826	YP_007002210.1	2e-74 (100%)	Staphylococcus phage GH15
Gp093	47450..47821	hypothetical protein	123	14479	YP_009195910.1	2e-85 (100%)	Staphylococcus phage phiIPLA-RODI
Gp094	47825..49516	portal protein	563	64075	YP_007002212.1	0.0 (100%)	Staphylococcus phage GH15
Gp095	49710..50483	prohead protease	257	28624	YP_007002213.1	0.0 (100%)	Staphylococcus phage GH15
Gp096	50502..51461	hypothetical protein	319	36116	YP_009098016.1	0.0 (100%)	Staphylococcus phage MCE-2014
Gp097	51577..52968	major capsid protein	463	51259	YP_009098017.1	0.0 (100%)	Staphylococcus phage MCE-2014
Gp098	53060..53356	hypothetical protein	98	11215	YP_009098018.1	9e-63 (100%)	Staphylococcus phage MCE-2014
Gp099	53369..54277	hypothetical protein	302	34161	YP_240905.1	0.0 (100%)	Staphylococcus virus G1
Gp100	54291..55169	hypothetical protein	292	33716	YP_009099551.1	0.0 (100%)	Staphylococcus phage P108
Gp101	55169..55789	hypothetical protein	206	23773	YP_009098021.1	2e-151 (100%)	Staphylococcus phage MCE-2014
Gp102	55808..56644	hypothetical protein	278	31768	YP_240908.1	0.0 (100%)	Staphylococcus virus G1
Gp103	56646..56861	hypothetical protein	71	8280	YP_240909.1	7e-48 (100%)	Staphylococcus virus G1
Gp104	56888..58651	major tail sheath protein	587	64418	YP_009195921.1	0.0 (100%)	Staphylococcus phage phiIPLA-RODI
Gp105	58724..59152	tail tube protein	142	15925	YP_240911.1	2e-102 (100%)	Staphylococcus virus G1
Gp106	59237..59401	hypothetical protein	54	6739	YP_007002224.1	5e-31 (100%)	Staphylococcus phage GH15
Gp107	59391..59531	hypothetical protein	46	5420	AFN38132.1	4e-20 (100%)	Staphylococcus phage A3R
Gp108	59573..60031	hypothetical protein	152	18111	YP_240913.1	4e-107 (100%)	Staphylococcus virus G1
Gp109	60044..60238	putative membrane protein	64	7125	BBC69542.1	2e-33 (100%)	Staphylococcus phage phiSA039
Gp110	60254..60406	hypothetical protein	50	5818	YP_009098028.1	3e-30 (100%)	Staphylococcus phage MCE-2014
Gp111	60474..60785	hypothetical protein	103	12238	YP_007002227.1	1e-68 (100%)	Staphylococcus phage GH15
Gp112	60917..61375	hypothetical protein	152	18108	YP_007002228.1	5e-108 (100%)	Staphylococcus phage GH15
Gp113	61419..61955	tail morphogenetic protein	178	20963	YP_009098031.1	1e-128 (100%)	Staphylococcus phage MCE-2014
Gp114	62008..66066	tail tape measure protein	1352	1E+05	AUV56888.1	0.0 (100%)	Staphylococcus phage vB_SauM_LM12
Gp115	66145..68571	N-acetylmuramoyl-L-alanine amidase	808	91281	ASZ78029.1	0.0 (100%)	Staphylococcus phage SA3
Gp116	68585..69472	protease	295	34503	YP_009098034.1	0.0 (100%)	Staphylococcus phage MCE-2014
Gp117	69472..72018	lycerophosphoryl diester phosphodiester	848	95994	YP_009098035.1	0.0 (100%)	Staphylococcus phage MCE-2014
Gp118	72125..72916	hypothetical protein	263	29329	YP_009099345.1	0.0 (100%)	Staphylococcus phage P108
Gp119	72916..73440	hypothetical protein	174	19953	YP_240925.1	4e-124 (100%)	Staphylococcus virus G1
Gp120	73440..74144	baseplate wedge subunit protein	234	26584	YP_240926.1	5e-174 (100%)	Staphylococcus virus G1
Gp121	74159..75205	putative tail protein	348	39179	YP_007002237.1	0.0 (100%)	Staphylococcus phage GH15
Gp122	75226..78291	hypothetical protein	1021	1E+05	YP_009098040.1	0.0 (100%)	Staphylococcus phage MCE-2014
Gp123	78402..78923	hypothetical protein	173	19239	YP_240929.1	5e-125 (100%)	Staphylococcus virus G1
Gp124	78944..82402	adsorption-associated tail protein	1152	1E+05	YP_009195940.1	0.0 (100%)	Staphylococcus phage phiIPLA-RODI
Gp125	82451..82609	hypothetical protein	52	6305	YP_009099353.1	1e-27 (100%)	Staphylococcus phage P108
Gp126	82610..84532	bohydrate binding domain-containing pr	640	72571	YP_009099354.1	0.0 (100%)	Staphylococcus phage P108
Gp127	84546..84920	hypothetical protein	124	14650	YP_009098045.1	1e-87 (100%)	Staphylococcus phage MCE-2014
Gp128	84927..86303	putative capsid and scaffold protein	458	50436	YP_009098046.1	0.0 (100%)	Staphylococcus phage MCE-2014
Gp129	86394..88142	DNA helicase A	582	67202	YP_009098047.1	0.0 (100%)	Staphylococcus phage MCE-2014
Gp130	88154..89767	putative Rep protein	537	63147	YP_009098048.1	0.0 (100%)	Staphylococcus phage MCE-2014
Gp131	89760..91202	DNA helicase B	480	54613	YP_009195947.1	0.0 (100%)	Staphylococcus phage phiIPLA-RODI
Gp132	91281..91700	hypothetical protein	139	16206	YP_009195948.1	4e-98 (100%)	Staphylococcus phage phiIPLA-RODI
Gp133	91700..92725	exonuclease	341	39342	YP_009195949.1	0.0 (100%)	Staphylococcus phage phiIPLA-RODI
Gp134	92725..93102	hypothetical protein	125	15027	YP_009195950.1	2e-84 (100%)	Staphylococcus phage phiIPLA-RODI
Gp135	93102..95021	putative recombination exonuclease B	639	73264	YP_009098052.1	0.0 (100%)	Staphylococcus phage MCE-2014
Gp136	95021..95602	HNH homing endonuclease	193	22894	ANH50485.1	7e-132 (100%)	Staphylococcus phage pSco-10
Gp137	95602..96198	hypothetical protein	198	23207	YP_009098053.1	7e-145 (100%)	Staphylococcus phage MCE-2014
Gp138	96213..97280	DNA primase	355	41040	YP_009195953.1	0.0 (100%)	Staphylococcus phage phiIPLA-RODI
Gp139	97346..97684	hypothetical protein	112	12964	YP_240943.1	3e-74 (100%)	Staphylococcus virus G1
Gp140	97684..98136	hypothetical protein	150	17044	AUV56979.1	2e-103 (100%)	Staphylococcus phage vB_SauM_LM12
Gp141	98123..98731	resolvase	202	23640	YP_009195956.1	2e-150 (100%)	Staphylococcus phage phiIPLA-RODI
Gp142	98748..99140	ribonucleotide reduction protein NrdI	130	14737	YP_009098058.1	1e-90 (100%)	Staphylococcus phage MCE-2014

Stab21

Gp	Genomic location	Predicted function	AA	MW	Best hit (acc no)	value (query coverage)	phage with similar gene
Gp001	485..646	hypothetical protein	53	6072	YP_007112871.1	9e-31 (100%)	Staphylococcus phage JD007
Gp002	741..1049	hypothetical protein	102	11792	YP_007112870.1	6e-67 (100%)	Staphylococcus phage JD007
Gp003	1061..1360	hypothetical protein	99	11562	YP_007112869.1	1e-63 (100%)	Staphylococcus phage JD007
Gp004	1376..1561	TreB	61	6855	YP_007112868.1	3e-31 (100%)	Staphylococcus phage JD007
Gp005	1669..1989	hypothetical protein	106	12354	AVX47357.1	4e-46 (100%)	Staphylococcus phage vB_SauM_0414_108
Gp006	2004..2297	hypothetical protein	97	11604	BBC69665.1	7e-64 (100%)	Staphylococcus phage phiSA039
Gp007	2301..2558	TreF	85	10310	YP_009196055.1	1e-53 (100%)	Staphylococcus phage phiIPLA-RODI
Gp008	2672..2911	hypothetical protein	79	8973	YP_009196056.1	1e-41 (100%)	Staphylococcus phage phiIPLA-RODI
Gp009	2922..3269	hypothetical protein	115	13689	YP_009098142.1	4e-77 (100%)	Staphylococcus phage Team1
Gp010c	c(3476..3814)	hypothetical protein	112	13472	YP_009196058.1	1e-71 (100%)	Staphylococcus phage phiIPLA-RODI
Gp011	4125..4433	TreI	102	11768	AFN37829.1	5e-69 (100%)	Staphylococcus phage StaphIN
Gp012	4640..4927	hypothetical protein	95	11102	YP_007112861.1	1e-63 (100%)	Staphylococcus phage JD007
Gp013	4977..5249	hypothetical protein	90	10539	ARM69069.1	1e-57 (100%)	Staphylococcus phage vB_Sau_CG
Gp014	5773..5931	hypothetical protein	52	6070	YP_007112859.1	9e-27 (100%)	Staphylococcus phage JD007
Gp015	6098..6421	TreP	107	12352	YP_007112857.1	9e-72 (100%)	Staphylococcus phage JD007
Gp016	6515..7027	hypothetical protein	170	20780	AXU40178.1	1e-108 (98%)	Staphylococcus phage VB_SavM_JYL01
Gp017	7091..7447	hypothetical protein	118	13865	VEV88121.1	7e-71 (99%)	Staphylococcus phage Stab20
Gp018	7977..8198	hypothetical protein	73	8469	YP_007112855.1	2e-45 (100%)	Staphylococcus phage JD007
Gp019	8279..8416	hypothetical protein	45	5459	YP_007112854.1	4e-24 (100%)	Staphylococcus phage JD007
Gp020	8478..8651	hypothetical protein	57	6765	VEV88124.1	1e-31 (100%)	Staphylococcus phage Stab20
Gp021	8904..9374	hypothetical protein	156	17885	AVX47376.1	5e-103 (100%)	Staphylococcus phage vB_SauM_0414_108
Gp022	9434..9631	hypothetical protein	65	7481	VEV88129.1	5e-33 (100%)	Staphylococcus phage Stab20
Gp023	9637..9819	hypothetical protein	60	6962	VEV88130.1	5e-31 (100%)	Staphylococcus phage Stab20
Gp024	9819..10475	hypothetical protein	218	25574	AUV56944.1	6e-72 (97%)	Staphylococcus phage vB_SauM_LM12
Gp025	10483..10758	TreT	91	10518	YP_009195839.1	4e-23 (100%)	Staphylococcus phage phiIPLA-RODI
Gp026	10834..11022	hypothetical protein	62	7512	YP_007002139.1	1e-35 (100%)	Staphylococcus phage GH15
Gp027c	c(11360..11596)	hypothetical protein	78	9557	AVX47381.1	1e-49 (100%)	Staphylococcus phage vB_SauM_0414_108
Gp028c	c(11598..12083)	hypothetical protein	161	19058	YP_241045.1	4e-111 (100%)	Staphylococcus virus G1
Gp029c	c(12096..12593)	hypothetical protein	135	16464	YP_008873525.1	4e-94 (100%)	Staphylococcus phage Sb1
Gp030c	c(12593..12934)	hypothetical protein	143	17323	AFN38052.1	2e-94 (100%)	Staphylococcus phage A3R
Gp031c	c(13125..13373)	hypothetical protein	82	9987	QAU05802.1	3e-49 (100%)	Staphylococcus virus Sa87
Gp032c	c(13373..13855)	membrane protein	160	18385	YP_007112840.1	7e-106 (100%)	Staphylococcus phage JD007
Gp033c	c(13848..14279)	hypothetical protein	143	16785	YP_008854159.1	4e-96 (100%)	Staphylococcus phage S25-3
Gp034c	c(14293..14835)	hypothetical protein	180	21586	YP_007112838.1	3e-125 (100%)	Staphylococcus phage JD007
Gp035c	c(14847..15335)	hypothetical protein	162	19434	QAU05805.1	4e-118 (100%)	Staphylococcus virus Sa87
Gp036c	c(15348..15746)	hypothetical protein	132	16141	YP_007112836.1	3e-89 (100%)	Staphylococcus phage JD007
Gp037c	c(15743..16450)	Serine/threonine protein phosphatase	235	27697	YP_007112835.1	7e-172 (100%)	Staphylococcus phage JD007
Gp038c	c(16541..18391)	lipase acylhydrolase domain protein	616	68944	YP_007112834.1	0.0 (100%)	Staphylococcus phage JD007
Gp039c	c(19301..19849)	hypothetical protein	182	21954	YP_007112833.1	1e-124 (100%)	Staphylococcus phage JD007
Gp040c	c(19853..20071)	hypothetical protein	72	8425	YP_007002157.1	8e-44 (100%)	Staphylococcus phage GH15
Gp041c	c(20072..20266)	hypothetical protein	64	7641	YP_241059.1	3e-38 (100%)	Staphylococcus virus G1
Gp042c	c(20256..20993)	hypothetical protein	245	28633	YP_007112830.1	1e-174 (100%)	Staphylococcus phage JD007
Gp043c	c(21172..21411)	hypothetical protein	79	9369	AFN38067.1	2e-51 (100%)	Staphylococcus phage A3R
Gp044c	c(21413..21802)	hypothetical protein	129	15153	YP_008854169.1	2e-89 (100%)	Staphylococcus phage S25-3
Gp045c	C (21901..22074)	hypothetical protein	57	6819	YP_007002163.1	2e-34 (100%)	Staphylococcus phage GH15
Gp046c	C (22115..22597)	hypothetical protein	160	18855	YP_008873543.1	4e-110 (100%)	Staphylococcus phage Sb1
Gp047c	c(22647..23189)	hypothetical protein	180	20415	ARM69103.1	1e-123 (100%)	Staphylococcus phage vB_Sau_CG
Gp048c	c(23189..23722)	hypothetical protein	177	20707	YP_241067.1	2e-124 (100%)	Staphylococcus virus G1
Gp049c	c(23725..23889)	hypothetical protein	54	6196	YP_008854174.1	5e-30 (100%)	Staphylococcus phage S25-3
Gp050c	c(23892..24170)	putative membrane protein	92	10955	AFN37865.1	4e-54 (100%)	Staphylococcus phage StaphIN
Gp051c	c(24170..25015)	hypothetical protein	281	31748	YP_241070.1	0.0 (100%)	Staphylococcus virus G1
Gp052c	c(25027..26154)	AAA family ATPase	375	42599	YP_007112820.1	0.0 (99%)	Staphylococcus phage JD007
Gp053c	c(26298..26624)	hypothetical protein	108	12980	YP_241072.1	4e-73 (100%)	Staphylococcus virus G1
Gp054c	c(26617..27033)	hypothetical protein	138	15979	YP_241073.1	8e-96 (100%)	Staphylococcus virus G1
Gp055c	c(27166..27468)	nucleoside triphosphate pyrophosphohydrolase	100	11304	ARM69324.1	1e-64 (100%)	Staphylococcus phage vB_Sau_Clo6
Gp056c	c(27468..27656)	hypothetical protein	62	7309	YP_007112816.1	3e-35 (100%)	Staphylococcus phage JD007
Gp057c	c(27700..27861)	hypothetical protein	53	6370	YP_241076.1	1e-29 (100%)	Staphylococcus virus G1
Gp058c	c(27861..29909)	hypothetical protein	682	79800	AZB49981.1	0.0 (100%)	Staphylococcus phage 812h1
Gp059c	c(29987..30250)	hypothetical protein	87	10147	YP_007112813.1	6e-54 (100%)	Staphylococcus phage JD007
Gp060c	c(30267..30440)	hypothetical protein	57	6670	YP_008854008.1	1e-31 (100%)	Staphylococcus phage S25-4
Gp061c	c(30447..31025)	hypothetical protein	192	21478	YP_007112811.1	4e-131 (100%)	Staphylococcus phage JD007
Gp062c	c(31018..31641)	hypothetical protein	207	23650	VEV89232.1	4e-129 (100%)	Staphylococcus phage Stab22
Gp063c	c(31641..32201)	HNH homing endonuclease	186	21870	AXY83933.1	1e-100 (100%)	Staphylococcus phage Terranova
Gp064c	c(32241..33137)	RNA ligase	298	35071	YP_008854188.1	0.0 (100%)	Staphylococcus phage S25-3
Gp065c	c(33137..33361)	hypothetical protein	74	8165	YP_008873561.1	2e-40 (100%)	Staphylococcus phage Sb1
Gp066c	c(33430..34170)	ate starvation-inducible protein PhoH, predicted	246	28575	YP_009097984.1	0.0 (100%)	Staphylococcus phage MCE-2014
Gp067c	c(34222..34836)	hypothetical protein	204	23008	YP_008854191.1	1e-145 (100%)	Staphylococcus phage S25-3
Gp068c	c(34852..35277)	ribonuclease H	141	15795	YP_007112805.1	2e-95 (100%)	Staphylococcus phage JD007
Gp069c	c(35267..35458)	hypothetical protein	63	7444	YP_008854193.1	2e-37 (100%)	Staphylococcus phage S25-3
Gp070c	c(35481..36122)	hypothetical protein	213	24573	YP_007112803.1	2e-144 (100%)	Staphylococcus phage JD007
Gp071c	c(36112..36342)	transcriptional regulator protein	76	8831	YP_007112802.1	3e-47 (100%)	Staphylococcus phage JD007
Gp072c	c(36345..36572)	hypothetical protein	75	9225	YP_008854019.1	2e-45 (100%)	Staphylococcus phage S25-4
Gp073c	c(36681..37373)	transglycosylase	230	24826	YP_009098202.1	2e-167 (100%)	Staphylococcus phage Team1
Gp074c	c(37571..38365)	membrane protein	264	29509	YP_007112798.1	0.0 (100%)	Staphylococcus phage JD007
Gp075c	c(38365..38673)	hypothetical protein	102	12135	YP_007112797.1	4e-65 (100%)	Staphylococcus phage JD007
Gp076c	c(38786..40273)	N-acetylmuramoyl-L-alanine amidase	495	54754	YP_007112796.1	0.0 (100%)	Staphylococcus phage JD007
Gp077c	c(40273..40776)	holin	167	18096	AUV56969.1	5e-117 (100%)	Staphylococcus phage vB_SauM_LM12
Gp078c	c(40861..41046)	hypothetical protein	61	7066	YP_007002196.1	3e-34 (100%)	Staphylococcus phage GH15
Gp079c	c(42594..42812)	hypothetical protein	72	8691	YP_009097998.1	2e-45 (100%)	Staphylococcus phage MCE-2014
Gp080c	c(43292..43501)	hypothetical protein	69	8019	YP_008873578.1	5e-43 (100%)	Staphylococcus phage Sb1
Gp081c	c(43514..43846)	hypothetical protein	110	12477	YP_008854029.1	3e-69 (100%)	Staphylococcus phage S25-4
Gp082c	c(43859..44185)	hypothetical protein	108	13056	YP_007002200.1	5e-71 (100%)	Staphylococcus phage GH15
Gp083c	c(44218..44484)	hypothetical protein	88	10121	YP_241102.1	1e-50 (100%)	Staphylococcus virus G1
Gp084	44625..45011	membrane protein	128	14819	YP_008854032.1	3e-82 (100%)	Staphylococcus phage S25-4
Gp085	44989..45267	hypothetical protein	92	10579	YP_008873583.1	3e-61 (100%)	Staphylococcus phage Sb1
Gp086	45264..45674	hypothetical protein	136	15626	YP_007112787.1	7e-92 (100%)	Staphylococcus phage JD007
Gp087	45689..47506	Terminase, large subunit	605	70243	YP_009041305.1	0.0 (100%)	Staphylococcus virus K
Gp088	47499..48320	hypothetical protein	273	30649	YP_009098222.1	0.0 (100%)	Staphylococcus phage Team1
Gp089	48307..48480	hypothetical protein	57	6674	YP_240894.1	1e-29 (100%)	Staphylococcus virus G1
Gp090	48477..48956	hypothetical protein	159	18524	YP_007112783.1	2e-110 (100%)	Staphylococcus phage JD007
Gp091	48999..50189	hypothetical protein	396	43647	YP_008854038.1	0.0 (100%)	Staphylococcus phage S25-4
Gp092	50275..50616	hypothetical protein	113	12852	YP_240898.1	4e-72 (100%)	Staphylococcus G1
Gp093	50634..51005	hypothetical protein	123	14479	YP_009195910.1	2e-83 (100%)	Staphylococcus phage phiIPLA-RODI
Gp094	51009..52700	Portal protein	563	64051	YP_240900.1	0.0 (100%)	Staphylococcus virus G1
Gp095	52894..53667	Prohead protease	257	28624	YP_007002213.1	0.0 (100%)	Staphylococcus phage GH15
Gp096	53686..54642	hypothetical protein	318	36016	YP_007112777.1	0.0 (100%)	Staphylococcus phage JD007
Gp097	54758..56149	Major capsid protein	463	51211	YP_007112776.1	0.0 (100%)	Staphylococcus phage JD007
Gp098	56241..56537	hypothetical protein	98	11257	YP_007112775.1	5e-60 (100%)	Staphylococcus phage JD007
Gp099	56550..57458	hypothetical protein	302	34161	YP_240905.1	0.0 (100%)	Staphylococcus virus G1
Gp100	57472..58350	hypothetical protein	292	33758	YP_007112773.1	0.0 (100%)	Staphylococcus phage JD007
Gp101	58350..58970	hypothetical protein	206	23746	YP_240907.1	5e-149 (100%)	Staphylococcus virus G1
Gp102	58989..59825	hypothetical protein	278	31782	YP_240908.1	0.0 (100%)	Staphylococcus virus G1
Gp103	59827..60042	hypothetical protein	71	8280	YP_240909.1	6e-46 (100%)	Staphylococcus virus G1
Gp104	60069..61832	major tail sheath protein	587	64458	YP_007112769.1	0.0 (100%)	Staphylococcus phage JD007
Gp105	61905..62333	tail tube protein	142	15925	YP_009041323.1	1e-100 (100%)	Staphylococcus virus K
Gp106	62430..62570	hypothetical protein	46	5408	YP_008873604.1	2e-23 (100%)	Staphylococcus phage Sb1
Gp107	62613..63071	hypothetical protein	152	18131	EF136582.1	3e-97 (100%)	Staphylococcus phage 812 strain phi812
Gp108	63084..63278	hypothetical protein	64	7157	YP_240914.1	7e-35 (100%)	Staphylococcus virus G1
Gp109	63360..63671	hypothetical protein	103	12252	YP_008854056.1	1e-6 (100%)	Staphylococcus phage S25-4
Gp110	63803..64261	hypothetical protein*	152	18122	YP_008854057.1	5e-106 (100%)	Staphylococcus phage S25-4
Gp111	64305..64841	tail morphogenetic protein	178	20915	YP_009195929.1	2e-125 (100%)	Staphylococcus phage phiIPLA-RODI
Gp112	64894..68952	putative tail lysin	1352	1E+05	YP_007002230.1	0.0 (100%)	Staphylococcus phage GH15
Gp113	69031..71457	tail lysin	808	91180	YP_007112760.1	0.0 (100%)	Staphylococcus phage JD007
Gp114	71471..72358	protease	295	34593	YP_009041332.1	0.0 (100%)	Staphylococcus virus K
Gp115	72358..74904	Glycerophosphoryl diester phosphodiesterase	848	96085	YP_007112758.1	0.0 (100%)	Staphylococcus phage JD007
Gp116	75011..75802	hypothetical protein	263	29543	YP_240924.1	0.0 (100%)	Staphylococcus virus G1
Gp117	75802..76326	hypothetical protein	174	19953	YP_007002235.1	3e-122 (100%)	Staphylococcus phage GH15
Gp118	76326..77030	putative baseplate protein	234	26584	YP_008873616.1	4e-172 (100%)	Staphylococcus phage Sb1
Gp119	77045..78091	baseplate	348	39179	ARM68952.1	0.0 (100%)	Staphylococcus phage vB_Sau_CG
Gp120	78112..81171	hypothetical protein	1019	1E+05	YP_009041338.1	0.0 (100%)	Staphylococcus virus K
Gp121	81282..81803	hypothetical protein	173	19239	YP_007002239.1	4e-123 (100%)	Staphylococcus phage GH15
Gp122	81824..85282	adsorption-associated tail protein	1152	1E+05	ARM68955.1	0.0 (100%)	Staphylococcus phage vB_Sau_CG
Gp123	85331..85489	hypothetical protein	52	6208	YP_240931.1	3e-27 (100%)	Staphylococcus virus G1
Gp124	85490..87412	hypothetical protein	640	72602	YP_007112749.1	0.0 (100%)	Staphylococcus phage JD007
Gp125	87435..87806	hypothetical protein	123	14496	YP_007112748.1	9e-84 (100%)	Staphylococcus phage JD007
Gp126	87813..89189	hypothetical protein	458	50465	YP_008854075.1	0.0 (100%)	Staphylococcus phage S25-4
Gp127	89280..91028	DNA helicase A	582	67219	AKC02275.1	0.0 (100%)	Staphylococcus phage IME-SA1
Gp128	91040..92653	putative Rep protein	537	63159	YP_007002246.1	0.0 (100%)	Staphylococcus phage GH15
Gp129	92646..94088	DNA helicase B	480	54558	AVX47482.1	0.0 (100%)	Staphylococcus phage vB_SauM_0414_108
Gp130	94167..95192	putative exonuclease	341	39267	YP_007002248.1	0.0 (100%)	Staphylococcus phage GH15
Gp131	95192..95569	hypothetical protein	125	14912	YP_007112960.1	2e-86 (100%)	Staphylococcus phage JD007
Gp132	95569..97488	exonuclease	639	73446	YP_007112959.1	0.0 (100%)	Staphylococcus phage JD007
Gp133	97488..98084	hypothetical protein	198	23180	YP_007112958.1	3e-143 (100%)	Staphylococcus phage JD007
Gp134	98099..99166	DNA primase	355	40927	YP_007112957.1	0.0 (100%)	Staphylococcus phage JD007
Gp135	99232..99570	hypothetical protein	112	12964	YP_240943.1	2e-72 (100%)	Staphylococcus virus G1
Gp136	99570..100022	hypothetical protein	150	17012	YP_008854085.1	2e-101 (100%)	Staphylococcus phage S25-4
Gp137	100009..100617	resolvase	202	23613	YP_008873634.1	1e-148 (100%)	Staphylococcus phage Sb1
Gp138	100634..101026	Ribonucleotide reduction protein NrdI	130	14734	ABL87151.1	2e-88 (100%)	Staphylococcus phage 812
Gp139	101041..103155	nucleotide reductase of class Ib (aerobic), alpha s	704	80291	YP_009006789.1	0.0 (100%)	Staphylococcus phage phiSA12
Gp140	103169..104218	nucleotide reductase of class Ib (aerobic), beta s	349	40446	YP_007002259.1	0.0 (100%)	Staphylococcus phage GH15
Gp141	104236..104565	hypothetical protein	109	12384	YP_007112950.1	5e-73 (100%)	Staphylococcus phage JD007
Gp142	104549..104869	thioredoxin-like protein	106	12059	YP_008873639.1	1e-69 (100%)	Staphylococcus phage Sb1

Stab22

Gp	Genomic location	Predicted function	AA	MW	Best hit acc. No.	value (query coverage)	phage with similar gene
Gp001	312..491	hypothetical protein	59	7075	YP_008854130.1	3e-15 (96%)	Staphylococcus phage S25-4
Gp002	715..1239	hypothetical protein	174	19562	AVP40463.1	4e-69 (98%)	Staphylococcus phage phiSA_BS1
Gp003	1307..1519	hypothetical protein	70	7958	.....	.....	.....
Gp004	1869..2162	TreA	97	11137	ARM69483.1	2e-56 (100%)	Staphylococcus phage vB_Sau_S24
Gp005	2159..2344	membrane protein	61	7046	YP_008853952.1	8e-17 (98%)	Staphylococcus phage S25-4
Gp006	2347..2520	membrane protein	57	6631	ARM69272.1	5e-17 (100%)	Staphylococcus phage vB_Sau_Clo6
Gp007	2532..2828	hypothetical protein	98	11140	AXU40163.1	1e-50 (100%)	Staphylococcus phage VB_SavM_JYL01
Gp008	3043..3246	putative membrane protein	67	7957	.....	.....	.....
Gp009	3259..3573	hypothetical protein	104	11887	ARM69275.1	2e-72 (100%)	Staphylococcus phage vB_Sau_Clo6
Gp010	3587..3886	hypothetical protein	99	11591	ARM69488.1	8e-63 (100%)	Staphylococcus phage vB_Sau_S24
Gp011	3903..4190	TreC	95	10992	ARM69276.1	4e-51 (100%)	Staphylococcus phage vB_Sau_Clo6
Gp012	4190..4486	TreE	98	11398	ARM69278.1	3e-34 (100%)	Staphylococcus phage vB_Sau_Clo6
Gp013	4511..4738	TreF	75	8713	ARM69279.1	1e-44 (100%)	Staphylococcus phage vB_Sau_Clo6
Gp014	4775..4985	terminal repeat-encoded protein	76	9014	YP_009099458.1	3e-21 (100%)	Staphylococcus phage P108
Gp015	5069..5332	hypothetical protein	87	10195	YP_008853956.1	1e-37 (91%)	Staphylococcus phage S25-4
Gp016	5332..5583	terminal repeat-encoded protein	83	9443	BBC69667.1	2e-18 (100%)	Staphylococcus phage phiSA039
Gp017c	(5834..6118)c	hypothetical protein	94	11231	ASZ78147.1	1e-54 (100%)	Staphylococcus phage SA3
Gp018	6518..6832	TreJ	104	12109	ARM69283.1	4e-60 (98%)	Staphylococcus phage vB_Sau_Clo6
Gp019	6939..7409	hypothetical protein	156	18824	AVP40358.1	4e-92 (100%)	Staphylococcus phage phiSA_BS1
Gp020	7483..7737	hypothetical protein	84	9973	AVR55650.1	5e-40 (100%)	Staphylococcus phage phiSA_BS2
Gp021	8265..8555	hypothetical protein	96	11308	.....	.....	.....
Gp022	8892..9143	hypothetical protein	83	9758	AVP40364.1	2e-37 (100%)	Staphylococcus phage phiSA_BS1
Gp023	9214..9621	hypothetical protein	135	15710	YP_009097937.1	1e-83 (98%)	Staphylococcus phage MCE-2014
Gp024	10117..10434	hypothetical protein	105	11917	BBC69674.1	1e-49 (98%)	Staphylococcus phage phiSA039
Gp025	10512..10748	hypothetical protein	78	9068	ARM69294.1	6e-37 (100%)	Staphylococcus phage vB_Sau_Clo6
Gp026	10836..11318	terminal repeat-encoded protein	160	18554	YP_009195837.1	4e-76 (99%)	Staphylococcus phage phiIPLA-RODI
Gp027	11399..11587	hypothetical protein	62	7258	VEV88129.1	2e-33 (100%)	Staphylococcus phage Stab20
Gp028	11600..11869	TreT protein	89	10190	VEV88131.1	1e-48 (100%)	Staphylococcus phage Stab20
Gp029	11956..12186	TreU protein	76	9177	VEV88132.1	2e-36 (100%)	Staphylococcus phage Stab20
Gp030c	c(12448..12711)	BofL	87	10640	YP_007002141.1	1e-24 (100%)	Staphylococcus phage GH15
Gp031c	c(12727..12972)	hypothetical protein	81	9652	AXU40190.1	4e-52 (100%)	Staphylococcus phage VB_SavM_JYL01
Gp032c	c(12972..13403)	hypothetical protein	143	17363	YP_009195845.1	2e-86 (100%)	Staphylococcus phage phiIPLA-RODI
Gp033c	c(13400..13837)	hypothetical protein	145	16823	AVR55457.1	2e-92 (97%)	Staphylococcus phage phiSA_BS2
Gp034c	c(13851..14393)	hypothetical protein	180	21507	YP_007002151.1	3e-124 (100%)	Staphylococcus phage GH15
Gp035c	c(14405..14893)	GTP cyclohydrolase II	162	19487	ARM69517.1	6e-116 (100%)	Staphylococcus phage vB_Sau_S24
Gp036c	c(15045..15755)	Serine/threonine phosphatase protein	236	27927	YP_009006684.1	8e-158 (100%)	Staphylococcus phage phiSA12
Gp037c	c(16912..17460)	hypothetical protein	182	21968	YP_008853987.1	1e-108 (100%)	Staphylococcus phage S25-4
Gp038c	c(17591..17830)	hypothetical protein	79	9442	YP_007002161.1	7e-48 (100%)	Staphylococcus phage GH15
Gp039c	c(17832..18218)	hypothetical protein	128	14789	ASZ78180.1	8e-78 (100%)	Staphylococcus phage SA3
Gp040c	c(18218..18491)	hypothetical protein	57	6819	YP_007002163.1	3e-36 (100%)	Staphylococcus phage GH15
Gp041c	c(18532..19014)	hypothetical protein	160	19046	VEV88152.1	2e-107 (100%)	Staphylococcus phage Stab20
Gp042c	c(19064..19600)	hypothetical protein	178	20718	ARM69316.1	6e-79 (99%)	Staphylococcus phage vB_Sau_Clo6
Gp043c	c(19600..20133)	hypothetical protein	177	20637	ARM69317.1	8e-117 (98%)	Staphylococcus phage vB_Sau_Clo6
Gp044c	c(20136..20300)	putative membrane protein	54	6286	YP_007002167.1	3e-26 (100%)	Staphylococcus phage GH15
Gp045c	c(20300..20599)	putative membrane protein	98	11605	ARM69530.1	2e-35 (100%)	Staphylococcus phage vB_Sau_S24
Gp046c	c(20599..21444)	hypothetical protein	281	31668	ARM69531.1	0.0 (100%)	Staphylococcus phage vB_Sau_S24
Gp047c	c(21457..22575)	AAA family ATPase	372	42054	ARM69321.1	0.0 (100%)	Staphylococcus phage vB_Sau_Clo6
Gp048c	c(22729..23070)	hypothetical protein	113	13285	VEV88159.1	6e-72 (100%)	Staphylococcus phage Stab20
Gp049c	c(23048..23464)	hypothetical protein	138	15941	YP_007002172.1	3e-95 (100%)	Staphylococcus phage GH15
Gp050c	c(23598..23900)	NTP pyrophosphohydrolase	100	11304	YP_007002173.1	4e-66 (99%)	Staphylococcus phage GH15
Gp051c	c(23900..24088)	hypothetical protein	62	7292	ARM69325.1	1e-35 (100%)	Staphylococcus phage vB_Sau_Clo6
Gp052c	c(24132..24293)	hypothetical protein	53	6447	ARM69537.1	9e-31 (100%)	Staphylococcus phage vB_Sau_S24
Gp053c	c(24294..26345)	hypothetical protein	683	79762	YP_008854006.1	0.0 (100%)	Staphylococcus phage S25-4
Gp054c	c(26422..26685)	hypothetical protein	87	10190	ARM69328.1	1e-55 (100%)	Staphylococcus phage vB_Sau_Clo6
Gp055c	c(26702..26875)	LysM domain-containing protein	57	6656	YP_007002178.1	5e-33 (100%)	Staphylococcus phage GH15
Gp056c	c(26882..27460)	membrane protein	192	21498	ARM69541.1	3e-130 (100%)	Staphylococcus phage vB_Sau_S24
Gp057c	c(27453..28055)	nucleoside 2-deoxyribosyltransferase protein	200	22430	VEV88168.1	4e-116 (100%)	Staphylococcus phage Stab20
Gp058c	c(28055..28192)	hypothetical protein	45	4936	VEV88417.1	6e-23 (100%)	Staphylococcus phage Stab23
Gp059c	c(28194..28604)	hypothetical protein	136	15636	AVP40314.1	3e-90 (100%)	Staphylococcus phage phiSA_BS1
Gp060c	c(28604..28828)	putative membrane protein	74	8129	ARM69543.1	1e-36 (100%)	Staphylococcus phage vB_Sau_S24
Gp061c	c(28896..29636)	PhoH-related protein	246	28646	ARM69333.1	0.0 (100%)	Staphylococcus phage vB_Sau_Clo6
Gp062c	c(29690..30265)	hypothetical protein	191	21477	ARM69334.1	6e-129 (100%)	Staphylococcus phage vB_Sau_Clo6
Gp063c	c(30283..30708)	ribonuclease H	141	15844	ARM69546.1	5e-96 (100%)	Staphylococcus phage vB_Sau_S24
Gp064c	c(30701..30889)	hypothetical protein	62	7442	ARM69336.1	2e-36 (100%)	Staphylococcus phage vB_Sau_Clo6
Gp065c	c(30912..31553)	hypothetical protein	213	24475	ARM69337.1	7e-144 (100%)	Staphylococcus phage vB_Sau_Clo6
Gp066c	c(31543..31773)	transcriptional regulator	76	8831	YP_007112802.1	4e-49 (100%)	Staphylococcus phage JD007
Gp067c	c(31776..32003)	hypothetical protein	75	9231	YP_007002190.1	4e-44 (100%)	Staphylococcus phage GH15
Gp068c	c(32113..32811)	transglycosylase	232	25289	ARM69551.1	3e-168 (100%)	Staphylococcus phage vB_Sau_S24
Gp069c	c(33001..33795)	putative membrane protein	264	29353	VEV89254.1	0.0 (100%)	Staphylococcus phage Stab23
Gp070c	c(33796..34104)	putative membrane protein	102	12254	AUV57026.1	2e-63 (100%)	Staphylococcus phage vB_SauM_LM12
Gp071c	c(34219..34514)	hypothetical protein	98	11766	VEV89258.1	8e-65 (100%)	Staphylococcus phage Stab23
Gp072c	c(34616..36106)	N-acetylmuramoyl-L-alanine amidase	496	54904	YP_009097995.1	0.0 (100%)	Staphylococcus phage MCE-2014
Gp073c	c(36106..36609)	holin	167	18068	YP_009195894.1	2e-116 (100%)	Staphylococcus phage phiIPLA-RODI
Gp074c	c(36695..36880)	hypothetical protein	61	7052	YP_007002196.1	7e-36 (100%)	Staphylococcus phage GH15
Gp075c	c(38219..38437)	hypothetical protein	72	8709	YP_007002197.1	5e-47 (100%)	Staphylococcus phage GH15
Gp076c	c(38906..39115)	hypothetical protein	69	7871	ANH50542.1	1e-41 (100%)	Staphylococcus phage pSco-10
Gp077c	c(39128..39460)	hypothetical protein	110	12563	YP_007002199.1	1e-69 (100%)	Staphylococcus phage GH15
Gp078c	c(39473..39799)	putative membrane protein	108	13157	ARM69350.1	1e-71 (100%)	Staphylococcus phage vB_Sau_Clo6
Gp079	40239..40625	membrane protein	128	14809	YP_009195900.1	2e-57 (100%)	Staphylococcus phage phiIPLA-RODI
Gp080	40603..40881	hypothetical protein	92	10610	ANH50538.1	4e-61 (100%)	Staphylococcus phage pSco-10
Gp081	40878..41288	hypothetical protein	136	15698	ANH50537.1	8e-93 (100%)	Staphylococcus phage pSco-10
Gp082	41303..43120	terminase, large subunit	605	70430	ARM69355.1	0.0 (100%)	Staphylococcus phage vB_Sau_S24
Gp083	43134..43934	hypothetical protein	266	29765	ARM69356.1	0.0 (100%)	Staphylococcus phage vB_Sau_S24
Gp084	43921..44094	hypothetical protein	57	6755	ARM69144.1	1e-28 (100%)	Staphylococcus phage vB_Sau_Clo6
Gp085	44091..44570	hypothetical protein	159	18521	YP_007002206.1	1e-109 (100%)	Staphylococcus phage GH15
Gp086	44663..45823	hypothetical protein	386	42608	AUV56911.1	4e-146 (100%)	Staphylococcus phage vB_SauM_LM12
Gp087	45962..46252	membrane protein	96	11038	ARM69147.1	3e-59 (100%)	Staphylococcus phage vB_Sau_Clo6
Gp088	46258..46629	hypothetical protein	123	14479	YP_009195910.1	2e-85 (100%)	Staphylococcus phage phiIPLA-RODI
Gp089	46633..48324	portal protein	563	63969	ARM69149.1	0.0 (100%)	Staphylococcus phage vB_Sau_Clo6
Gp090	48518..49282	prohead protease	254	28019	ARM69150.1	0.0 (100%)	Staphylococcus phage vB_Sau_Clo6
Gp091	49301..50260	hypothetical protein	319	36065	ARM69364.1	0.0 (100%)	Staphylococcus phage vB_Sau_S24
Gp092	50376..51767	major capsid protein	463	51298	ANH50522.1	0.0 (100%)	Staphylococcus phage pSco-10
Gp093	51859..52131	hypothetical protein	90	10280	ANH50521.1	1e-42 (100%)	Staphylococcus phage pSco-10
Gp094	52144..53052	hypothetical protein	302	34108	ARM69154.1	0.0 (100%)	Staphylococcus phage vB_Sau_Clo6
Gp095	53066..53944	capsid protein	292	33715	ARM69368.1	0.0 (100%)	Staphylococcus phage vB_Sau_S24
Gp096	53944..54564	hypothetical protein	206	23735	ANH50518.1	2e-147 (100%)	Staphylococcus phage pSco-10
Gp097	54583..55419	hypothetical protein	278	31816	YP_007112771.1	0.0 (100%)	Staphylococcus phage JD007
Gp098	55421..55636	hypothetical protein	71	8252	YP_007002221.1	3e-47 (100%)	Staphylococcus phage GH15
Gp099	55663..57426	major tail sheath	587	64225	ASZ78017.1	0.0 (100%)	Staphylococcus phage SA3
Gp100	57499..57909	tail tube protein	136	15202	AFN38130.1	3e-90 (100%)	Staphylococcus phage A3R
Gp101	58431..59729	hypothetical protein	432	50246	YP_238556.1	5e-156 (99%)	Staphylococcus virus Twort
Gp102	59784..59942	hypothetical protein	52	6502	YP_009006753.1	1e-29 (100%)	Staphylococcus phage phiSA12
Gp103	59932..60069	hypothetical protein	45	5334	AFN38132.1	8e-19 (100%)	Staphylococcus phage A3R
Gp104	60103..60564	hypothetical protein	153	18043	ARM68939.1	4e-104 (98%)	Staphylococcus phage vB_Sau_CG
Gp105	60577..60771	membrane protein	64	6992	ANH50510.1	3e-34 (100%)	Staphylococcus phage pSco-10
Gp106	60842..61153	hypothetical protein	106	12160	YP_007002227.1	2e-62 (100%)	Staphylococcus phage GH15
Gp107	61285..61740	hypothetical protein	151	17978	ARM69381.1	2e-104 (100%)	Staphylococcus phage vB_Sau_S24
Gp108	61775..62320	tail morphogenetic protein	181	21234	AXU40054.1	3e-129 (100%)	Staphylococcus phage VB_SavM_JYL01
Gp109	62373..66422	tail length tape-measure protein	1349	143659	ARM69383.1	0.0 (100%)	Staphylococcus phage vB_Sau_S24
Gp110	66502..68925	tail lysin	807	91433	ASZ78029.1	0.0 (100%)	Staphylococcus phage SA3
Gp111	68939..69826	protease	295	34633	YP_009195932.1	0.0 (100%)	Staphylococcus phage phiIPLA-RODI
Gp112	69826..72372	Glycerophosphoryl diester phosphodiesterase	848	96077	ARM69386.1	0.0 (100%)	Staphylococcus phage vB_Sau_S24
Gp113	72479..73270	hypothetical protein	263	29292	ARM69174.1	0.0 (100%)	Staphylococcus phage vB_Sau_Clo6
Gp114	73270..73794	hypothetical protein	174	19948	ARM69175.1	2e-123 (100%)	Staphylococcus phage vB_Sau_Clo6
Gp115	73794..74498	baseplate wedge subunit protein	234	26539	ARM69389.1	5e-172 (100%)	Staphylococcus phage vB_Sau_S24
Gp116	74513..75559	baseplate morphogenetic protein	348	39121	ARM69177.1	0.0 (100%)	Staphylococcus phage vB_Sau_Clo6
Gp117	75580..77820	hypothetical protein	746	85246	YP_008854069.1	0.0 (100%)	Staphylococcus phage S25-4
Gp118	77928..78449	structural protein	173	19298	BBC69556.1	2e-124 (100%)	Staphylococcus phage phiSA039
Gp119	78470..81946	adsorption-associated tail protein	1158	129844	ARM69393.1	0.0 (100%)	Staphylococcus phage vB_Sau_S24
Gp120	81995..82153	hypothetical protein	52	6277	YP_008854072.1	3e-29 (100%)	Staphylococcus phage S25-4
Gp121	82154..84073	hypothetical protein	639	73210	ARM69182.1	0.0 (100%)	Staphylococcus phage vB_Sau_Clo6
Gp122	84087..84452	hypothetical protein	121	14388	ARM69183.1	5e-74 (100%)	Staphylococcus phage vB_Sau_Clo6
Gp123	84459..85832	tail fiber protein	457	50960	ARM69184.1	0.0 (100%)	Staphylococcus phage vB_Sau_Clo6
Gp124	85921..87669	DNA helicase A	582	67211	ARM69399.1	0.0 (100%)	Staphylococcus phage vB_Sau_S24
Gp125	87681..89294	Rep protein	537	63201	ARM69186.1	0.0 (100%)	Staphylococcus phage vB_Sau_Clo6
Gp126	89287..90729	DNA helicase B	480	54531	ARM69187.1	0.0 (100%)	Staphylococcus phage vB_Sau_Clo6
Gp127	90808..91089	hypothetical protein	93	10916	AVP40297.1	4e-54 (100%)	Staphylococcus phage phiSA_BS1
Gp128	91089..92114	recombination exonuclease A	341	39505	YP_008854079.1	0.0 (100%)	Staphylococcus phage S25-4
Gp129	92114..94033	recombination exonuclease B	639	73035	ARM69190.1	0.0 (100%)	Staphylococcus phage vB_Sau_Clo6
Gp130	94033..94629	anti-sigma factor	198	23365	ARM69192.1	2e-133 (100%)	Staphylococcus phage vB_Sau_Clo6
Gp131	94644..95711	DNA primase	355	40979	ANH50483.1	0.0 (100%)	Staphylococcus phage pSco-10
Gp132	95777..96115	hypothetical protein	112	12964	YP_240943.1	2e-72 (100%)	Staphylococcus virus G1
Gp133	96115..96567	hypothetical protein	150	17155	ANH50481.1	5e-99 (100%)	Staphylococcus phage pSco-10
Gp134	96554..97162	resolvase	202	23617	ANH50480.1	1e-147 (100%)	Staphylococcus phage pSco-10
Gp135	97179..97571	Ribonucleotide reduction protein Class Ib, NrdI	130	14764	ARM69197.1	1e-90 (100%)	Staphylococcus phage vB_Sau_Clo6
Gp136	97586..99700	Ribonucleotide reductase, large subunit	704	80063	ARM69198.1	0.0 (100%)	Staphylococcus phage vB_Sau_Clo6
Gp137	99714..100763	Ribonucleotide reductase, small subunit	349	40410	YP_009099373.1	0.0 (100%)	Staphylococcus phage P108
Gp138	100781..101110	hypothetical protein	109	12401	ARM69413.1	5e-75 (100%)	Staphylococcus phage vB_Sau_S24
Gp139	101094..101414	thioredoxin	106	12048	ARM69201.1	3e-70 (100%)	Staphylococcus phage vB_Sau_Clo6
Gp140	101622..102218	hypothetical protein	198	23602	YP_007002262.1	2e-141 (100%)	Staphylococcus phage GH15
Gp141	102228..102533	integration host factor	101	11839	ANH50469.1	2e-67 (100%)	Staphylococcus phage pSco-10
Gp142	102609..105827	DNA polymerase A	1072	124521	ARM69417.1	0.0 (100%)	Staphylococcus phage vB_Sau_S24

Stab23

Gp	Genomic location	Predicted function	AA	MW	Best hit acc. No.	value (query coverage)	phage with similar gene
Gp001	553..1536	hypothetical protein	327	36796	ARM69482.1	1e-119 (79%)	Staphylococcus phage vB_Sau_S24
Gp002	1766..2059	hypothetical protein	97	11159	ARM69271.1	6e-58 (100%)	Staphylococcus phage vB_Sau_Clo6
Gp003	2056..2217	putative membrane protein	53	5951	ARM69484.1	2e-26 (98%)	Staphylococcus phage vB_Sau_S24
Gp004	2314..2499	putative membrane protein	61	7238	....	....	....
Gp005	2515..2829	hypothetical protein	104	11954	ARM69275.1	1e-70 (100%)	Staphylococcus phage vB_Sau_Clo6
Gp006	2843..3136	hypothetical protein	97	11232	ARM69277.1	5e-55 (100%)	Staphylococcus phage vB_Sau_Clo6
Gp007	3140..3391	hypothetical protein	83	9746	ARM69279.1	2e-52 (100%)	Staphylococcus phage vB_Sau_Clo6
Gp008	3479..3727	hypothetical protein	82	9860	ARM69280.1	4e-37 (100%)	Staphylococcus phage vB_Sau_Clo6
Gp009	3740..3976	hypothetical protein	78	8862	ARM69493.1	2e-50 (100%)	Staphylococcus phage vB_Sau_S24
Gp010c	(4219..4551)c	hypothetical protein	110	13307	ARM69282.1	8e-61 (100%)	Staphylococcus phage vB_Sau_Clo6
Gp011	4862..5170	TreI	102	11938	ARM69283.1	8e-64 (100%)	Staphylococcus phage vB_Sau_Clo6
Gp012	5362..5835	hypothetical protein	157	18855	ARM69285.1	3e-101 (98%)	Staphylococcus phage vB_Sau_Clo6
Gp013	5892..6137	hypothetical protein	81	9901	AVP40359.1	3e-36 (96%)	Staphylococcus phage phiSA_BS1
Gp014	6995..7153	TreN	52	5985	ARM69287.1	2e-27 (100%)	Staphylococcus phage vB_Sau_Clo6
Gp015	7227..7535	hypothetical protein	102	11944	ARM69500.1	2e-68 (100%)	Staphylococcus phage vB_Sau_Clo6
Gp016	7698..8024	TreP	108	12401	ARM69501.1	1e-71 (100%)	Staphylococcus phage vB_Sau_S24
Gp017	8123..8440	hypothetical protein	105	12181	BBC69674.1	8e-33 (100%)	Staphylococcus phage phiSA039
Gp018	8514..8921	hypothetical protein	135	15740	ARM69291.1	1e-84 (98%)	Staphylococcus phage vB_Sau_Clo6
Gp019	9497..9751	hypothetical protein	84	9730	ARM69292.1	3e-56 (100%)	Staphylococcus phage vB_Sau_Clo6
Gp020	9965..10243	hypothetical protein	92	10789	VEV88348.1	2e-51 (100%)	Staphylococcus phage Stab20
Gp021	10335..10808	hypothetical protein	157	18016	ARM69078.1	8e-87 (100%)	Staphylococcus phage vB_Sau_CG
Gp022	10887..11051	hypothetical protein	54	6181	ARM69297.1	1e-29 (100%)	Staphylococcus phage vB_Sau_Clo6
Gp023	11064..11327	hypothetical protein	87	10250	ARM69079.1	6e-55 (100%)	Staphylococcus phage vB_Sau_CG
Gp024	11331..11519	hypothetical protein	62	7173	VEV88129.1	2e-33 (100%)	Staphylococcus phage Stab20
Gp025	11532..11801	TreT	89	10112	ARM69081.1	6e-55 (100%)	Staphylococcus phage vB_Sau_CG
Gp026	11886..12107	TreU	73	8944	VEV88132.1	6e-45 (100%)	Staphylococcus phage Stab20
Gp027c	(12388..12678)c	hypothetical protein	96	11594	VEV88133.1	5e-62 (100%)	Staphylococcus phage Stab20
Gp028c	(12775..14487)c	putative tail fiber protein	570	64852	YP_009097947.1	2e-61 (24%)	Staphylococcus phage MCE-2014
Gp029c	(14555..14806)c	BofL	83	10015	ARM69301.1	6e-51 (97%)	Staphylococcus phage vB_Sau_Clo6
Gp030c	(14822..15067)c	hypothetical protein	81	9681	YP_008853977.1	7e-52 (100%)	Staphylococcus phage S25-4
Gp031c	(15067..15588)c	hypothetical protein	173	20527	YP_009099479.1	2e-80 (99%)	Staphylococcus phage P108
Gp032c	(15594..16073)c	putative membrane protein	159	17910	ARM69303.1	5e-105 (100%)	Staphylococcus phage vB_Sau_Clo6
Gp033c	(16066..16320)c	hypothetical protein	84	9675	ASZ78168.1	8e-49 (100%)	Staphylococcus phage SA3]
Gp034c	(16320..16754)c	hypothetical protein	144	16762	YP_007002150.1	1e-87 (100%)	Staphylococcus phage GH15
Gp035c	(16769..17263)c	GTP cyclohydrolase II	164	19751	ARM69306.1	3e-118 (100%)	Staphylococcus phage vB_Sau_Clo6
Gp036c	(17278..17730)c	hypothetical protein	150	17704	YP_009097955.1	2e-92 (100%)	Staphylococcus phage MCE-2014
Gp037c	(18836..19381)c	hypothetical protein	181	21873	YP_008853987.1	6e-100 (92%)	Staphylococcus phage S25-4
Gp038c	(19385..19600)c	hypothetical protein	71	8347	ARM69310.1	4e-41 (100%)	Staphylococcus phage vB_Sau_Clo6
Gp039c	(19597..20346)c	hypothetical protein	249	29109	YP_009195858.1	8e-151 (98%)	Staphylococcus phage phiIPLA-RODI
Gp040c	(20483..20722)c	hypothetical protein	79	9398	YP_007002161.1	8e-47 (100%)	Staphylococcus phage GH15
Gp041c	(20724..21110)c	hypothetical protein	128	14679	ARM69313.1	7e-84 (100%)	Staphylococcus phage vB_Sau_Clo6
Gp042c	(21208..21381)c	hypothetical protein	57	6861	ARM69314.1	3e-34 (100%)	Staphylococcus phage vB_Sau_Clo6
Gp043c	(21422..21904)c	hypothetical protein	160	19093	ARM69525.1	1e-110 (100%)	Staphylococcus phage vB_Sau_Clo6
Gp044c	(21954..22490)c	hypothetical protein	178	20200	YP_008853995.1	5e-108 (100%)	Staphylococcus phage S25-4
Gp045c	(22490..23023)c	hypothetical protein	177	20640	ARM69317.1	3e-118 (100%)	Staphylococcus phage vB_Sau_Clo6
Gp046c	(23193..23474)c	putative membrane protein	93	11124	ARM69530.1	7e-58 (100%)	Staphylococcus phage vB_Sau_S24
Gp047c	(23474..24319)c	hypothetical protein	281	31672	ARM69320.1	0.0 (100%)	Staphylococcus phage vB_Sau_Clo6
Gp048c	(24331..25461)c	AAA family ATPase	376	42536	ARM69321.1	0.0 (100%)	Staphylococcus phage vB_Sau_Clo6
Gp049c	(25612..25938)c	hypothetical protein	108	12813	ARM69322.1	3e-71 (100%)	Staphylococcus phage vB_Sau_Clo6
Gp050c	(25931..26347)c	hypothetical protein	138	16029	ARM69323.1	3e-97 (100%)	Staphylococcus phage vB_Sau_Clo6
Gp051c	(26482..26784)c	nucleoside triphosphate pyrophosphohydrolase	100	11290	YP_007002173.1	7e-66 (100%)	Staphylococcus phage GH15
Gp052c	(26784..26972)c	hypothetical protein	62	7293	YP_007112816.1	3e-35 (100%)	Staphylococcus phage JD007
Gp053c	(27016..27177)c	hypothetical protein	53	6336	ARM69326.1	8e-25 (100%)	Staphylococcus phage vB_Sau_Clo6
Gp054c	(27178..29229)c	hypothetical protein	683	80127	ARM69327.1	0.0 (100%)	Staphylococcus phage vB_Sau_Clo6
Gp055c	(29307..29570)c	hypothetical protein	87	10232	ARM69539.1	2e-56 (100%)	Staphylococcus phage vB_Sau_S24
Gp056c	(29587..29760)c	Lysin	57	6628	YP_007002178.1	1e-33 (100%)	Staphylococcus phage GH15
Gp057c	(29767..30345)c	putative membrane protein	192	21438	ARM69541.1	8e-133 (100%)	Staphylococcus phage vB_Sau_S24
Gp058c	(30338..30961)c	nucleoside 2-deoxyribosyltransferase	207	23457	VEV88533.1	5e-131 (100%)	Staphylococcus phage Stab21
Gp059c	(30961..31098)c	hypothetical protein	45	5041	AVP40312.1	4e-17 (97%)	Staphylococcus phage phiSA_BS1
Gp060c	(31100..31324)c	putative membrane protein	74	8080	ARM69119.1	5e-37 (100%)	Staphylococcus phage vB_Sau_CG
Gp061c	(31392..32132)c	PhoH-related protein	246	28760	ARM69333.1	0.0 (100%)	Staphylococcus phage vB_Sau_Clo6
Gp062c	(32184..32894)c	hypothetical protein	236	27107	ARM69545.1	2e-167 (100%)	Staphylococcus phage vB_Sau_S24
Gp063c	(32912..33337)c	ribonuclease H	141	15774	ARM69546.1	2e-98 (100%)	Staphylococcus phage vB_Sau_S24
Gp064c	(33330..33518)c	hypothetical protein	62	7505	ARM69336.1	6e-39 (100%)	Staphylococcus phage vB_Sau_Clo6
Gp065c	(33541..34182)c	hypothetical protein	213	24475	ARM69548.1	4e-146 (100%)	Staphylococcus phage vB_Sau_S24
Gp066c	(34172..34402)c	transcriptional regulator	76	8833	YP_007002189.1	2e-48 (100%)	Staphylococcus phage GH15
Gp067c	(34405..34632)c	hypothetical protein	75	9235	BBC69504.1	2e-47 (100%)	Staphylococcus phage phiSA039
Gp068c	(34741..35430)c	transglycosylase	229	25065	BBC69505.1	3e-149 (100%)	Staphylococcus phage phiSA039
Gp069c	(35625..36419)c	putative membrane protein	264	29297	ARM69552.1	0.0 (100%)	Staphylococcus phage vB_Sau_S24
Gp070c	(36420..36728)c	hypothetical protein	102	12173	YP_009099524.1	1e-67 (100%)	Staphylococcus phage P108
Gp071c	(36842..37462)c	hypothetical protein	206	24604	YP_009097994.1	2e-144 (100%)	Staphylococcus phage MCE-2014
Gp072c	(37525..39015)c	N-acetylmuramoyl-L-alanine amidase	496	54981	ARM69554.1	0.0 (99%)	Staphylococcus phage vB_Sau_S24
Gp073c	(39015..39518)c	holin	167	18111	ARM69345.1	1e-118 (100%)	Staphylococcus phage vB_Sau_Clo6
Gp074c	(39603..39788)c	hypothetical protein	61	7052	ARM69556.1	4e-36 (100%)	Staphylococcus phage vB_Sau_S24
Gp075c	(41015..41233)c	hypothetical protein	72	8665	ARM69347.1	4e-47 (100%)	Staphylococcus phage vB_Sau_Clo6
Gp076c	(41697..41906)c	hypothetical protein	69	7804	ARM69348.1	2e-42 (100%)	Staphylococcus phage vB_Sau_Clo6
Gp077c	(41919..42251)c	hypothetical protein	110	12491	ANH50541.1	1e-71 (100%)	Staphylococcus phage pSco-10
Gp078c	(42264..42590)c	hypothetical protein	108	13100	ARM69350.1	2e-73 (100%)	Staphylococcus phage vB_Sau_Clo6
Gp079	43149..43424	hypothetical protein	91	10695	ARM69352.1	6e-55 (94%)	Staphylococcus phage vB_Sau_Clo6
Gp080	43393..43680	hypothetical protein	95	10934	ANH50538.1	1e-62 (95%)	Staphylococcus phage pSco-10
Gp081	43677..44087	hypothetical protein	136	15726	ANH50537.1	2e-93 (100%)	Staphylococcus phage pSco-10
Gp082	44102..45919	terminase, large subunit	605	70485	ARM69142.1	0.0 (100%)	Staphylococcus phage vB_Sau_Clo6
Gp083	45912..46733	hypothetical protein	273	30512	YP_007002206.1	0.0 (100%)	Staphylococcus phage GH15
Gp084	46890..47369	hypothetical protein	159	18524	ARM69145.1	1e-110 (100%)	Staphylococcus phage vB_Sau_Clo6
Gp085	47412..48707	hypothetical protein	431	47006	ARM69359.1	0.0 (100%)	Staphylococcus phage vB_Sau_S24
Gp086	48789..49136	hypothetical protein	115	13154	ARM69147.1	1e-75 (100%)	Staphylococcus phage vB_Sau_Clo6
Gp087	49142..49513	hypothetical protein	123	14479	YP_009195910.1	2e-85 (100%)	Staphylococcus phage phiIPLA-RODI
Gp088	49517..51208	portal protein	563	64038	ARM69149.1	0.0 (100%)	Staphylococcus phage vB_Sau_Clo6
Gp089	51402..52166	prohead protease	254	28062	ARM69150.1	0.0 (100%)	Staphylococcus phage vB_Sau_Clo6
Gp090	52185..53135	hypothetical protein	316	35836	ARM69364.1	3e-159 (100%)	Staphylococcus phage vB_Sau_S24
Gp091	53251..54642	major capsid prontein	463	51260	ARM69152.1	0.0 (100%)	Staphylococcus phage vB_Sau_Clo6
Gp092	54734..55006	hypothetical protein	90	10382	ARM69153.1	8e-56 (100%)	Staphylococcus phage vB_Sau_Clo6
Gp093	55020..55928	hypothetical protein	302	34082	ARM69154.1	0.0 (100%)	Staphylococcus phage vB_Sau_Clo6
Gp094	55942..56820	capsid protein	292	33741	ARM69155.1	0.0 (100%)	Staphylococcus phage vB_Sau_Clo6
Gp095	56820..57440	hypothetical protein	206	23748	ARM69156.1	4e-151 (100%)	Staphylococcus phage vB_Sau_Clo6
Gp096	57459..58295	hypothetical protein	278	31794	ARM69157.1	0.0 (100%)	Staphylococcus phage vB_Sau_Clo6
Gp097	58297..58512	hypothetical protein	71	8260	YP_007002221.1	7e-48 (100%)	Staphylococcus phage GH15
Gp098	58539..60302	major tail sheath	587	64491	ARM69159.1	0.0 (100%)	Staphylococcus phage vB_Sau_Clo6
Gp099	60375..60803	tail tube protein	142	15871	ARM69160.1	5e-102 (100%)	Staphylococcus phage vB_Sau_Clo6
Gp100	60902..61039	hypothetical protein	45	5307	ANH50512.1	3e-24 (100%)	Staphylococcus phage pSco-10
Gp101	61073..61534	hypothetical protein	153	17945	ARM69163.1	3e-97 (98%)	Staphylococcus phage vB_Sau_Clo6
Gp102	61547..61789	hypothetical protein	85	8865	ARM68940.1	2e-50 (94%)	Staphylococcus phage vB_Sau_CG
Gp103	61789..61983	putative membrane protein	64	6950	ANH50510.1	6e-36 (100%)	Staphylococcus phage pSco-10
Gp104	61999..62151	hypothetical protein	50	5859	ARM69379.1	6e-30 (100%)	Staphylococcus phage vB_Sau_S24
Gp105	62219..62530	hypothetical protein	103	12190	ARM69380.1	3e-68 (100%)	Staphylococcus phage vB_Sau_S24
Gp106	62219..62530	hypothetical protein	151	18036	ARM69381.1	1e-106 (100%)	Staphylococcus phage vB_Sau_S24
Gp107	63161..63697	tail morphogenetic protein	178	21023	ARM69382.1	1e-128 (100%)	Staphylococcus phage vB_Sau_S24
Gp108	63751..67809	tail length tape-measure protein	1352	143711	ARM69383.1	0.0 (100%)	Staphylococcus phage vB_Sau_S24
Gp109	67889..70312	tail lysin	807	91344	ARM69171.1	0.0 (100%)	Staphylococcus phage vB_Sau_Clo6
Gp110	70326..71213	peptidoglycan hydrolase	295	34650	ARM69172.1	0.0 (100%)	Staphylococcus phage vB_Sau_Clo6
Gp111	71213..73759	Glycerophosphoryl diester phosphodiesterase	848	95925	ARM69173.1	0.0 (100%)	Staphylococcus phage vB_Sau_Clo6
Gp112	73866..74657	hypothetical protein	263	29277	ARM69174.1	0.0 (100%)	Staphylococcus phage vB_Sau_Clo6
Gp113	74657..75181	hypothetical protein	174	19939	ARM69388.1	4e-123 (100%)	Staphylococcus phage vB_Sau_S24
Gp114	75181..75885	baseplate wedge subunit	234	26526	ARM69176.1	1e-172 (100%)	Staphylococcus phage vB_Sau_Clo6
Gp115	75900..76946	baseplate morphogenetic protein	348	39110	ARM69177.1	0.0 (100%)	Staphylococcus phage vB_Sau_Clo6
Gp116	76967..80512	tail morphogenetic protein	1181	134937	ARM69391.1	0.0 (100%)	Staphylococcus phage vB_Sau_S24
Gp117	80623..81144	baseplate morphogenetic protein	173	19121	ARM68954.1	3e-124 (100%)	Staphylococcus phage vB_Sau_CG
Gp118	81165..84641	adsorption-associated tail protein	1158	129750	ARM69393.1	0.0 (100%)	Staphylococcus phage vB_Sau_S24
Gp119	84690..84848	hypothetical protein	52	6305	YP_009099353.1	2e-29 (100%)	Staphylococcus phage P108
Gp120	84849..86762	hypothetical protein	637	72509	ASK86679.1	0.0 (100%)	Staphylococcus phage vB_Sau_S24
Gp121	86776..87147	hypothetical protein	123	14553	ARM69397.1	2e-87 (100%)	Staphylococcus phage vB_Sau_S24
Gp122	87154..88527	tail fiber protein	457	50699	ARM69398.1	0.0 (100%)	Staphylococcus phage vB_Sau_S24
Gp123	88616..90364	DNA helicase A	582	67212	ARM69399.1	0.0 (100%)	Staphylococcus phage vB_Sau_S24
Gp124	90376..91989	replication protein	537	63285	ANH50490.1	0.0 (100%)	Staphylococcus phage pSco-10
Gp125	91982..93424	DNA helicase B	480	54588	ANH50489.1	0.0 (100%)	Staphylococcus phage pSco-10
Gp126	93504..93785	hypothetical protein	93	10859	ARM69188.1	1e-52 (100%)	Staphylococcus phage vB_Sau_Clo6
Gp127	93785..94810	recombination exonuclease A	341	39336	YP_009195949.1	0.0 (100%)	Staphylococcus phage phiIPLA-RODI
Gp128	94810..95187	hypothetical protein	125	15133	YP_009099363.1	7e-87 (100%)	Staphylococcus phage P108
Gp129	95187..97106	recombination exonuclease B	639	73229	ARM69404.1	0.0 (100%)	Staphylococcus phage vB_Sau_S24
Gp130	97106..97702	hypothetical protein	198	23207	YP_009098053.1	1e-143 (100%)	Staphylococcus phage MCE-2014
Gp131	97717..98784	DNA primase	355	40951	ANH50483.1	0.0 (100%)	Staphylococcus phage pSco-10
Gp132	98849..99187	hypothetical protein	112	12947	ARM69194.1	2e-73 (100%)	Staphylococcus phage vB_Sau_Clo6
Gp133	99187..99639	hypothetical protein	150	17126	ARM69195.1	3e-102 (100%)	Staphylococcus phage vB_Sau_Clo6
Gp134	99626..100234	resolvase	202	23692	ARM69196.1	1e-147 (100%)	Staphylococcus phage vB_Sau_Clo6
Gp135	100251..100643	ribonucleotide reduction protein NrdI	130	14738	ARM69410.1	2e-91 (100%)	Staphylococcus phage vB_Sau_S24
Gp136	100658..102772	ribonucleotide reductase, large subunit	704	79967	ARM69411.1	0.0 (100%)	Staphylococcus phage vB_Sau_S24
Gp137	102786..103835	ribonucleotide reductase, small subunit	349	40472	ARM69199.1	0.0 (100%)	Staphylococcus phage vB_Sau_Clo6
Gp138	103853..104182	hypothetical protein	109	12587	ARM69413.1	3e-74 (100%)	Staphylococcus phage vB_Sau_S24
Gp139	104166..104486	thioredoxin	106	12018	ARM69201.1	2e-70 (100%)	Staphylococcus phage vB_Sau_Clo6
Gp140	104694..105290	hypothetical protein	198	23582	ANH50470.1	4e-143 (100%)	Staphylococcus phage pSco-10
Gp141	105300..105605	DNA binding protein	101	11909	ARM69203.1	2e-68 (100%)	Staphylococcus phage vB_Sau_Clo6
Gp142	105681..108863	DNA polymerase A	1060	122611	ARM69417.1	0.0 (100%)	Staphylococcus phage vB_Sau_S24

Gp143	99155..101269	ribonucleotide reductase large subunit	704	80259	YP_009195958.1	0.0 (100%)	Staphylococcus phage philPLA-RODI
Gp144	101283..102332	ribonucleotide reductase small subunit	349	40444	YP_009195959.1	0.0 (100%)	Staphylococcus phage philPLA-RODI
Gp145	102350..102679	hypothetical protein	109	12458	AUV57013.1	1e-74 (100%)	Staphylococcus phage vB_SauM_LM12
Gp146	102663..102983	thioredoxin	106	12045	YP_009195961.1	2e-71 (100%)	Staphylococcus phage philPLA-RODI
Gp147	103190..103786	hypothetical protein	198	23600	YP_007002262.1	2e-143 (100%)	Staphylococcus phage GH15
Gp148	103796..104101	integration host factor	101	11928	YP_240952.1	9e-69 (100%)	Staphylococcus virus G1
Gp149	104177..107395	DNA polymerase A	1072	1E+05	YP_009195964.1	0.0 (100%)	Staphylococcus phage philPLA-RODI
Gp150	107423..107707	hypothetical protein	94	10843	YP_008854095.1	2e-51 (100%)	Staphylococcus phage S25-4
Gp151	107724..108206	hypothetical protein	160	18919	YP_007002266.1	3e-117 (100%)	Staphylococcus phage GH15
Gp152	108293..109600	hypothetical protein	435	48352	YP_009195967.1	0.0 (100%)	Staphylococcus phage philPLA-RODI
Gp153	109660..110916	DNA repair protein	418	46764	YP_009195968.1	0.0 (100%)	Staphylococcus phage philPLA-RODI
Gp154	110920..111273	hypothetical protein	117	13379	YP_240963.1	1e-81 (100%)	Staphylococcus virus G1
Gp155	111260..111922	RNA polymerase sigma factor	220	26610	YP_008873651.1	1e-158 (100%)	Staphylococcus phage Sb1
Gp156	112049..112681	hypothetical protein	210	23169	ASZ78069.1	3e-152 (100%)	Staphylococcus phage SA3
Gp157	112696..113217	tail protein	173	18161	AEA36766.1	2e-116 (100%)	Staphylococcus phage GH15
Gp158	113232..113459	Ig-like protein	75	7829	YP_007002273.1	6e-47 (100%)	Staphylococcus phage GH15
Gp159	113554..113814	hypothetical protein	86	10273	YP_007002274.1	2e-57 (100%)	Staphylococcus phage GH15
Gp160	113818..114573	hypothetical protein	251	29179	YP_007002275.1	4e-180 (100%)	Staphylococcus phage GH15
Gp161	114566..115816	metallophosphoesterase	416	47606	YP_007002276.1	0.0 (100%)	Staphylococcus phage GH15
Gp162	115830..116198	membrane protein	122	14010	YP_007002277.1	7e-83 (100%)	Staphylococcus phage GH15
Gp163	116185..116496	hypothetical protein	103	12038	YP_009099393.1	3e-70 (100%)	Staphylococcus phage P108
Gp164	116560..117096	hypothetical protein	178	20762	YP_009098082.1	1e-130 (100%)	Staphylococcus phage MCE-2014
Gp165	117089..117856	hypothetical protein	255	30073	YP_009098083.1	0.0 (100%)	Staphylococcus phage MCE-2014
Gp166	117834..118280	hypothetical protein	148	17367	YP_009099396.1	8e-106 (100%)	Staphylococcus phage P108
Gp167	118280..119143	hypothetical protein	287	32316	YP_007002282.1	0.0 (100%)	Staphylococcus phage GH15
Gp168	119515..120246	hypothetical protein	243	28351	ARQ96133.1	1e-175 (100%)	Staphylococcus phage qdsa002
Gp169	120264..120722	hypothetical protein	152	17850	YP_007002284.1	3e-108 (100%)	Staphylococcus phage GH15
Gp170	120787..121230	hypothetical protein	147	17537	YP_007002285.1	3e-101 (100%)	Staphylococcus phage GH15
Gp171	121247..121951	hypothetical protein	234	27445	YP_009098089.1	5e-170 (100%)	Staphylococcus phage MCE-2014
Gp172	122014..122412	putative membrane protein	132	15428	YP_009006819.1	9e-93 (100%)	Staphylococcus phage phiSA12
Gp173	122559..122801	hypothetical protein	80	9423	YP_009098091.1	3e-50 (100%)	Staphylococcus phage MCE-2014
Gp174	122806..123363	putative membrane protein	185	21691	YP_009099404.1	2e-133 (100%)	Staphylococcus phage P108
Gp175	123399..123575	hypothetical protein	58	6988	YP_009041391.1	7e-35 (100%)	Staphylococcus virus K
Gp176	123565..123816	putative membrane protein	83	9246	VEV88755.1	1e-50 (100%)	Staphylococcus phage Stab21
Gp177	123809..124042	hypothetical protein	77	8960	YP_009099407.1	2e-48 (100%)	Staphylococcus phage P108
Gp178	124124..124768	putative membrane protein	214	25205	YP_007002293.1	5e-151 (100%)	Staphylococcus phage GH15
Gp179	125044..125220	hypothetical protein	58	7005	YP_009006827.1	5e-33 (100%)	Staphylococcus phage phiSA12
Gp180	125213..125509	hypothetical protein	98	11451	YP_009195996.1	2e-64 (100%)	Staphylococcus phage philPLA-RODI
Gp181	125548..125739	putative membrane protein	63	7497	ACB89144.1	1e-34 (100%)	Staphylococcus phage A5W
Gp182	125752..126120	hypothetical protein	122	14160	YP_009098101.1	4e-83 (100%)	Staphylococcus phage MCE-2014
Gp183	126133..126480	hypothetical protein	115	12973	YP_009098102.1	2e-77 (100%)	Staphylococcus phage MCE-2014
Gp184	126486..126758	membrane protein	90	9942	YP_009196000.1	2e-53 (100%)	Staphylococcus phage philPLA-RODI]
Gp185	126819..127133	hypothetical protein	104	12493	VEV88776.1	1e-66 (100%)	Staphylococcus phage Stab21
Gp186	127148..127498	hypothetical protein	116	13682	YP_009099417.1	6e-77 (100%)	Staphylococcus phage P108
Gp187	127498..128100	hypothetical protein	200	23383	YP_007002302.1	2e-145 (100%)	Staphylococcus phage GH15
Gp188	128114..128293	hypothetical protein	59	7277	YP_009196003.1	8e-35 (100%)	Staphylococcus phage philPLA-RODI
Gp189	128296..128862	HNH endonuclease	188	21514	YP_009007668.1	1e-34 (96%)	Staphylococcus phage vB_SepS_SEP9
Gp190	129030..129440	membrane protein	136	15408	YP_009196004.1	9e-90 (100%)	Staphylococcus phage philPLA-RODI
Gp191	129442..129735	hypothetical protein	97	11644	AUV57037.1	1e-62 (100%)	Staphylococcus phage vB_SauM_LM12
Gp192	129752..130039	putative membrane protein	95	10554	YP_007112901.1	2e-59 (100%)	Staphylococcus phage JD007
Gp193	130050..130163	hypothetical protein	37	4422	YP_007002307.1	2e-13 (100%)	Staphylococcus phage GH15
Gp194	130156..130428	hypothetical protein	90	10423	AUV57045.1	1e-52 (100%)	Staphylococcus phage vB_SauM_LM12
Gp195	130443..131108	hypothetical protein	221	24930	AUV56940.1	1e-155 (100%)	Staphylococcus phage vB_SauM_LM12
Gp196	131185..131490	hypothetical protein	101	11684	AUV57027.1	3e-64 (100%)	Staphylococcus phage vB_SauM_LM12
Gp197	131490..131894	putative membrane protein	134	15205	AUV56992.1	6e-86 (100%)	Staphylococcus phage vB_SauM_LM12
Gp198	131899..132135	hypothetical protein	78	9159	AUV57061.1	2e-49 (100%)	Staphylococcus phage vB_SauM_LM12
Gp199	132132..132659	putative metallophosphatase	175	20607	AUV56964.1	7e-124 (100%)	Staphylococcus phage vB_SauM_LM12
Gp200	132640..132951	hypothetical protein	103	12523	AUV57022.1	1e-67 (100%)	Staphylococcus phage vB_SauM_LM12
Gp201	132997..133176	putative membrane protein	59	6342	YP_007002315.1	9e-28 (100%)	Staphylococcus phage GH15
Gp202	133191..133454	hypothetical protein	87	10223	AUV57050.1	1e-51 (100%)	Staphylococcus phage vB_SauM_LM12
Gp203	133457..133762	hypothetical protein	101	11520	YP_007002317.1	9e-41 (100%)	Staphylococcus phage GH15
Gp204	133840..133998	putative membrane protein	52	5706	YP_009098109.1	3e-23 (100%)	Staphylococcus phage MCE-2014
Gp205	134014..134238	hypothetical protein	74	8515	YP_009098110.1	9e-46 (100%)	Staphylococcus phage MCE-2014
Gp206	134251..134451	hypothetical protein	66	7600	YP_008873669.1	1e-41 (100%)	Staphylococcus phage Sb1
Gp207	134452..134742	putative membrane protein	96	11134	YP_007002323.1	1e-59 (100%)	Staphylococcus phage GH15
Gp208	134835..135128	hypothetical protein	97	11414	YP_009099439.1	2e-64 (100%)	Staphylococcus phage GH15
Gp209	135125..136033	Ribose-phosphate pyrophosphokinase	302	34960	YP_009196009.1	0.0 (100%)	Staphylococcus phage philPLA-RODI
Gp210	136051..138441	Nicotinamide phosphoribosyltransferase	796	92251	ARM69254.1	0.0 (100%)	Staphylococcus phage vB_Sau_Clo6
Gp211	138520..138765	hypothetical protein	81	9863	YP_009098116.1	1e-49 (100%)	Staphylococcus phage MCE-2014]
Gp212	138785..139189	hypothetical protein	134	16100	YP_009196017.1	5e-84 (100%)	Staphylococcus phage philPLA-RODI]
Gp213	139194..139448	hypothetical protein	84	9899	YP_009099444.1	1e-47 (100%)	Staphylococcus phage P108
Gp214	139469..139666	Hypothetical protein	65	7833	YP_009098118.1	1e-38 (100%)	Staphylococcus phage MCE-2014
Gp215	139732..140043	hypothetical protein	103	11624	YP_009098119.1	1e-66 (100%)	Staphylococcus phage MCE-2014
Gp216	140046..140555	hypothetical protein	169	20300	YP_009098120.1	4e-119 (100%)	Staphylococcus phage MCE-2014
Gp217	140557..140892	hypothetical protein	111	12900	YP_009098121.1	3e-70 (100%)	Staphylococcus phage MCE-2014
Gp218	140892..141110	hypothetical protein	72	8650	YP_007002335.1	7e-42 (100%)	Staphylococcus phage GH15
Gp219	141212..141436	hypothetical protein	74	8869	AUV57111.1	6e-15 (50%)	Staphylococcus phage vB_SauM_LM12
Gp220	141436..141630	hypothetical protein	64	7840	YP_008873679.1	7e-25 (100%)	Staphylococcus phage Sb1
Gp221	141654..141968	hypothetical protein	104	12080	AEJ79800.1	4e-61 (100%)	Staphylococcus phage Sb1
Gp222	141984..142274	hypothetical protein	96	11446	.....	...	.....
Gp223	142290..142460	hypothetical protein	56	6800	.....	.....	....
Gp001	143008..143178	Putative membrane protein	56	6298	AUV57100.1	1e-29 (100 %)	Staphylococcus phage vB_SauM_LM12
Gp002	143194..143487	Terminal repeat-encoded protein	97	11245	YP_009006863.1	5e-59 (100%)	Staphylococcus phage phiSA12
Gp003	143484..143669	hypothetical protein	61	6540	YP_009099453.1	2e-31 (100%)	Staphylococcus phage P108
Gp004	143699..143923	putative membrane protein	74	8889	TAG94958.1	0.70 (64%)	.....
Gp005	143939..144229	TreC	96	11361	AUV57038.1	7e-60 (100%)	Staphylococcus phage vB_SauM_LM12
Gp006	144229..144516	Terminal repeat-encoded protein	95	10858	YP_008853954.1	2e-61 (100%)	Staphylococcus phage S25-4
Gp007	144516..144809	Terminal repeat-encoded protein	97	11538	YP_009099457.1	7e-60 (100%)	Staphylococcus phage P108
Gp008	144813..145061	Terminal repeat-encoded protein	82	9964	YP_009006868.1	1e-48 (100%)	Staphylococcus phage phiSA12
Gp009	145138..145386	hypothetical protein	82	9382	YP_009099460.1	4e-38 (91%)	Staphylococcus phage vB_Sau_CG
Gp010	145383..145709	terminal repeat-encoded protein	108	13000	AUG85650.1	9e-63 (99%)	Staphylococcus phage phiSA12
Gp011c	(145928..146266)	hypothetical protein	112	13528	YP_007002124.1	1e-69 (100%)	Staphylococcus phage GH15
Gp012	146578..146886	TreJ	102	11808	YP_007112862.1	1e-68 (100%)	Staphylococcus phage JD007
Gp013	147092..147376	TreK	94	10985	YP_009196040.1	1e-62 (100%)	Staphylococcus phage philPLA-RODI
Gp014	147451..147642	Terminal repeat-encoded protein	63	7674	YP_007002127.1	9e-38 (100%)	Staphylococcus phage GH15
Gp015	148178..148336	hypothetical protein	52	6070	YP_008853962.1	1e-26 (100%)	Staphylococcus phage S25-4
Gp016	148502..148825	TreP	107	12453	YP_241037.1	9e-72 (100%)	Staphylococcus virus G1
Gp017	148919..149431	hypothetical protein	170	20668	AXU40178.1	2e-108 (98%)	Staphylococcus phage Stab21
Gp018	149495..149881	hypothetical protein	128	14985	YP_008853965.1	8e-85 (98%)	Staphylococcus phage Stab23
Gp019	150324..150545	hypothetical protein	73	8464	YP_008853966.1	1e-45 (100%)	Staphylococcus phage S25-4
Gp020	150626..150769	hypothetical protein	47	5668	YP_008853967.1	7e-24 (93%)	Staphylococcus phage S25-4
Gp021	150826..150999	hypothetical protein	57	6783	VEV88448.1	1e-31 (100%)	Staphylococcus phage Stab21
Gp022	151037..151315	hypothetical protein	78	9008	YP_009196049.1	6e-46 (100%)	Staphylococcus phage philPLA-RODI
Gp023	151406..151750	hypothetical protein	114	13653	ARM69507.1	2e-73 (100%)	Staphylococcus phage vB_Sau_S24
Gp024	151817..152089	hypothetical protein	90	10843	BBC69463.1	1e-50 (96%)	Staphylococcus phage phiSA039

Gp143	105112..105672	hypothetical protein	186	22107	YP_007002262.1	1e-132 (100%)	Staphylococcus phage GH15
Gp144	105682..105987	integration host factor	101	11928	YP_009098279.1	7e-67 (100%)	Staphylococcus phage Team1
Gp145	106063..109281	DNA polymerase I	1072	1E+05	YP_009006795.1	0.0 (100%)	Staphylococcus phage phiSA12
Gp146	109351..109593	hypothetical protein	80	9026	VEV88253.1	4e-50 (100%)	Staphylococcus phage Stab20
Gp147	109610..110092	hypothetical protein	160	18947	YP_009098069.1	1e-115 (100%)	Staphylococcus phage MCE-2014
Gp148	110179..111450	hypothetical protein	423	46908	YP_008854097.1	0.0 (100%)	Staphylococcus phage S25-4
Gp149	111510..112766	recombinase protein	418	46793	AZB49858.1	0.0 (100%)	Staphylococcus phage 812
Gp150	112770..113123	hypothetical protein	117	13379	YP_009041370.1	9e-80 (100%)	Staphylococcus virus K
Gp151	113110..113772	RNA polymerase sigma factor	220	26610	AQT25578.1	8e-157 (100%)	Staphylococcus phage pSa-3
Gp152	113899..114531	hypothetical protein	210	23172	YP_008854277.1	2e-150 (100%)	Staphylococcus phage S25-3
Gp153	114545..115066	Ig-like protein	173	18147	AEA36766.1	4e-116 (100%)	Staphylococcus phage GH15
Gp154	115081..115308	major tail protein	75	7787	YP_007112935.1	5e-45 (100%)	Staphylococcus phage JD007
Gp155	115403..115663	hypothetical protein	86	10273	YP_007002274.1	2e-55 (100%)	Staphylococcus phage GH15
Gp156	115667..116422	hypothetical protein	251	29112	YP_007112933.1	2e-180 (100%)	Staphylococcus phage JD007
Gp157	116415..117665	DNA polymerase	416	47534	YP_007112932.1	0.0 (100%)	Staphylococcus phage JD007
Gp158	117679..118047	membrane protein	122	14008	YP_007112931.1	2e-81 (100%)	Staphylococcus phage JD007
Gp159	118034..118345	hypothetical protein	103	12010	YP_008873658.1	1e-68 (100%)	Staphylococcus phage Sb1
Gp160	118409..118945	hypothetical protein	178	20824	YP_240973.1	6e-128 (100%)	Staphylococcus virus G1
Gp161	118938..119705	hypothetical protein	255	30046	YP_007112928.1	0.0 (100%)	Staphylococcus phage JD007
Gp162	119683..120129	hypothetical protein	148	17337	YP_008854111.1	4e-104 (100%)	Staphylococcus phage S25-4
Gp163	120129..120992	hypothetical protein	287	32356	YP_008854112.1	0.0 (100%)	Staphylococcus phage S25-4
Gp164	121364..122095	hypothetical protein	243	28351	ARQ96133.1	8e-174 (100%)	Staphylococcus phage qdsa002
Gp165	122113..122571	hypothetical protein	152	17850	YP_007002284.1	3e-106 (100%)	Staphylococcus phage GH15
Gp166	122636..123079	hypothetical protein	147	17497	YP_007112923.1	3e-99 (100%)	Staphylococcus phage JD007
Gp167	123096..123800	hypothetical protein	234	27400	YP_007112922.1	9e-169 (100%)	Staphylococcus phage JD007
Gp168	123862..124260	putative membrane protein	132	15429	YP_009098309.1	7e-91 (100%)	Staphylococcus phage Team1
Gp169	124407..124649	hypothetical protein	80	9421	YP_007112920.1	7e-49 (100%)	Staphylococcus phage JD007
Gp170	124654..125211	hypothetical protein	185	21702	ARM69003.1	9e-132 (100%)	Staphylococcus phage vB_Sau_CG
Gp171	125247..125423	hypothetical protein	58	6988	YP_240984.1	5e-33 (100%)	Staphylococcus virus G1
Gp172	125413..125664	hypothetical protein	83	9190	ARQ96141.1	5e-50 (100%)	Staphylococcus phage qdsa002
Gp173	125657..125890	hypothetical protein	77	8840	YP_008854298.1	8e-48 (100%)	Staphylococcus phage S25-3
Gp174	125971..126615	hypothetical protein	214	25138	YP_008854123.1	3e-148 (100%)	Staphylococcus phage S25-4
Gp175	126630..126878	hypothetical protein	82	9067	AVX47531.1	2e-45 (100%)	Staphylococcus phage vB_SauM_0414_108
Gp176	126890..127066	hypothetical protein	58	6991	YP_008854125.1	1e-31 (100%)	Staphylococcus phage S25-4
Gp177	127059..127355	hypothetical protein	98	11342	YP_240987.1	3e-63 (100%)	Staphylococcus virus G1
Gp178	127394..127585	Putative membrane protein	63	7511	ACB89144.1	4e-35 (100%)	Staphylococcus phage A5W
Gp179	127598..127966	hypothetical protein	122	14272	YP_007112910.1	1e-82 (100%)	Staphylococcus phage JD007
Gp180	127979..128326	hypothetical protein	115	13029	AFN38826.1	5e-77 (100%)	Staphylococcus phage MSA6
Gp181	128326..128604	hypothetical protein	92	10184	YP_009098321.1	1e-56 (100%)	Staphylococcus phage Team1
Gp182	128665..128979	hypothetical protein	104	12520	YP_008854307.1	1e-67 (97%)	Staphylococcus phage S25-3
Gp183	128994..129344	hypothetical protein	116	13711	YP_009041401.1	7e-77 (100%)	Staphylococcus virus K
Gp184	129344..129946	hypothetical protein	200	23356	YP_240994.1	2e-145 (100%)	Staphylococcus virus G1
Gp185	129960..130139	hypothetical protein	59	7275	YP_007112904.1	8e-35 (100%)	Staphylococcus phage JD007
Gp186	130366..130776	hypothetical protein	136	15400	YP_007112903.1	4e-91 (100%)	Staphylococcus phage JD007
Gp187	130778..131071	hypothetical protein	97	11672	YP_007112902.1	7e-63 (100%)	Staphylococcus phage JD007
Gp188	131088..131375	hypothetical protein	95	10540	YP_240999.1	1e-59 (100%)	Staphylococcus virus G1
Gp189	131492..131755	hypothetical protein	87	9918	YP_007112899.1	1e-53 (100%)	Staphylococcus phage JD007
Gp190	131833..132138	hypothetical protein	101	11777	YP_008854311.1	2e-65 (100%)	Staphylococcus phage S25-3
Gp191	132138..132542	hypothetical protein	134	15169	YP_007112897.1	3e-88 (100%)	Staphylococcus phage JD007
Gp192	132547..132783	hypothetical protein	78	9192	YP_007112896.1	2e-48 (100%)	Staphylococcus phage JD007
Gp193	132780..133307	Phosphoesterase	175	20597	YP_009006842.1	7e-126 (100%)	Staphylococcus phage phiSA12
Gp194	133288..133608	hypothetical protein	106	12898	YP_008854315.1	5e-68 (100%)	Staphylococcus phage S25-3
Gp195	133608..133838	hypothetical protein	76	8860	YP_008854316.1	3e-44 (100%)	Staphylococcus phage S25-3
Gp196	133891..134070	hypothetical protein	59	6422	AFN38011.1	1e-29 (100%)	Staphylococcus phage StaphIN
Gp197	134085..134348	hypothetical protein	87	10251	YP_007112891.1	7e-56% (100%)	Staphylococcus phage JD007
Gp198	134351..134668	hypothetical protein	105	11992	YP_007112890.1	1e-68 (100%)	Staphylococcus phage JD007
Gp199	134669..135349	hypothetical protein	226	25749	YP_007112889.1	8e-160 (100%)	Staphylococcus phage JD007
Gp200	135427..135585	membrane protein	52	5686	YP_007112888.1	2e-23 (100%)	Staphylococcus phage JD007
Gp201	135601..135825	hypothetical protein	74	8573	YP_007112887.1	2e-46 (100%)	Staphylococcus phage JD007
Gp202	135838..136038	hypothetical protein	66	7699	YP_008854323.1	9e-42 (100%)	Staphylococcus phage S25-3
Gp203	136039..136329	Putative membrane protein	96	11082	YP_009006850.1	2e-58 (100%)	Staphylococcus phage phiSA12
Gp204	136423..136731	hypothetical protein	102	12049	YP_007112884.1	5e-63 (100%)	Staphylococcus phage JD007
Gp205	136728..137636	Ribose-phosphate pyrophosphokinase	302	35262	YP_008854326.1	0.0 (100%)	Staphylococcus phage S25-3
Gp206	137651..138046	hypothetical protein	131	15360	BBC69643.1	2e-87 (100%)	Staphylococcus phage phiSA039
Gp207	138050..139519	Nicotinamide phosphoribosyltransferase	489	56176	YP_009006853.1	0.0 (100%)	Staphylococcus phage phiSA12
Gp208	139598..139843	hypothetical protein	81	9830	YP_008854328.1	2e-50 (100%)	Staphylococcus phage S25-3
Gp209	139860..140252	hypothetical protein	130	15352	YP_007112880.1	1e-86 (100%)	Staphylococcus phage JD007
Gp210	140254..140451	hypothetical protein	65	7838	YP_007112879.1	2e-39 (100%)	Staphylococcus phage JD007
Gp211	140516..140812	hypothetical protein	98	11330	YP_007112878.1	9e-64 (100%)	Staphylococcus phage JD007
Gp212	140816..141127	hypothetical protein	103	11715	YP_009006858.1	1e-66 (100%)	Staphylococcus phage phiSA12
Gp213	141130..141369	hypothetical protein	79	9779	BBC69651.1	2e-49 (100%)	Staphylococcus phage phiSA039
Gp214	141359..141514	hypothetical protein	51	6141	YP_007112875.1	2e-29 (100%)	Staphylococcus phage JD007
Gp215	141518..141712	hypothetical protein	64	7717	YP_009041432.1	3e-37 (100%)	Staphylococcus virus K
Gp216	141729..142082	hypothetical protein	117	13906	YP_007112873.1	4e-77 (100%)	Staphylococcus phage JD007
Gp217	142101..142487	hypothetical protein	128	15650	YP_007112872.1	5e-85 (100%)	Staphylococcus phage JD007
Gp001	143133..143294	hypothetical protein	53	6072	YP_007112871.1	9e-31 (100%)	Staphylococcus phage JD007
Gp002	143389..143697	hypothetical protein	102	11792	YP_007112870.1	6e-67 (100%)	Staphylococcus phage JD007
Gp003	143709..144008	hypothetical protein	99	11562	YP_007112869.1	1e-63 (100%)	Staphylococcus phage JD007
Gp004	144024..144209	TreB	61	6855	YP_007112868.1	3e-31 (100%)	Staphylococcus phage JD007
Gp005	144317..144637	hypothetical protein	106	12354	AVX47357.1	4e-46 (100%)	Staphylococcus phage vB_SauM_0414_108
Gp006	144652..144945	hypothetical protein	97	11604	BBC69665.1	7e-64 (100%)	Staphylococcus phage phiSA039
Gp007	144949..145206	hypothetical protein	85	10310	YP_009196035.1	1e-53 (100%)	Staphylococcus phage philPLA-RODI
Gp008	145320..145559	hypothetical protein	79	8973	YP_009196036.1	1e-41 (100%)	Staphylococcus phage philPLA-RODI
Gp009	145570..145917	hypothetical protein	115	13689	YP_009098142.1	4e-77 (100%)	Staphylococcus phage Team1
Gp010c	c(146124..146462)	hypothetical protein	112	13472	YP_009196038.1	1e-71 (100%)	Staphylococcus phage philPLA-RODI
Gp011	146773..147081	TreI	102	11768	AFN37829.1	5e-69 (100%)	Staphylococcus phage StaphIN
Gp012	147288..147575	hypothetical protein	95	11102	YP_007112861.1	1e-63 (100%)	Staphylococcus phage JD007
Gp013	147625..147897	hypothetical protein	90	10539	ARM69069.1	1e-57 (100%)	Staphylococcus phage vB_Sau_CG
Gp014	148421..148579	hypothetical protein	52	6070	YP_007112859.1	9e-27 (100%)	Staphylococcus phage JD007
Gp015	148746..149069	TreP	107	12352	YP_007112857.1	9e-72 (100%)	Staphylococcus phage JD007
Gp016	149163..149675	hypothetical protein	170	20780	AXU40178.1	1e-108 (98%)	Staphylococcus phage VB_SavM_JYL01
Gp017	149739..150095	hypothetical protein	118	13865	VEV88121.1	7e-71 (98%)	Staphylococcus phage Stab20
Gp018	150625..150846	hypothetical protein	73	8469	YP_007112855.1	2e-45 (100%)	Staphylococcus phage JD007
Gp019	150927..151064	hypothetical protein	45	5459	YP_007112854.1	4e-24 (100%)	Staphylococcus phage JD007
Gp020	151126..151299	hypothetical protein	57	6765	VEV88124.1	1e-31 (100%)	Staphylococcus phage Stab20
Gp021	151552..152022	hypothetical protein	156	17885	AVX47376.1	5e-103 (100%)	Staphylococcus phage vB_SauM_0414_108
Gp022	152082..152279	hypothetical protein	65	7481	VEV88129.1	5e-33 (100%)	Staphylococcus phage Stab20
Gp023	152285..152467	terminal repeat-encoded protein	60	6962	VEV88130.1	5e-31 (100%)	Staphylococcus phage Stab20
Gp024	152467..153123	hypothetical protein	218	25574	AUV56944.1	6e-72 (97%)	Staphylococcus phage vB_SauM_LM12
Gp025	153131..153406	Terminal repeat-encoded protein	91	10518	YP_009195839.1	4e-23 (100%)	Staphylococcus phage philPLA-RODI
Gp026	153482..153670	hypothetical protein	62	7512	YP_007002139.1	1e-35 (100%)	Staphylococcus phage GH15

Gp143	105896..106138	hypothetical protein	80	9144	ARM69418.1	1e-49 (100%)	Staphylococcus phage vB_Sau_S24
Gp144	106155..106637	hypothetical protein	160	18974	ANH50464.1	3e-117 (100%)	Staphylococcus phage pSco-10
Gp145	106724..107902	hypothetical protein	392	43625	ARM69420.1	0.0 (100%)	Staphylococcus phage vB_Sau_S24
Gp146	107962..109209	DNA repair recombinase protein	415	46753	YP_007002268.1	0.0 (100%)	Staphylococcus phage GH15
Gp147	109213..109566	hypothetical protein	117	13421	YP_007002269.1	2e-80 (99%)	Staphylococcus phage GH15
Gp148	109553..110215	RNA polymerase sigma factor	220	26600	ARM69210.1	2e-157 (100%)	Staphylococcus phage vB_Sau_Clo6
Gp149	110342..110974	hypothetical protein	210	23241	YP_008873652.1	2e-149 (100%)	Staphylococcus phage Sb1
Gp150	110987..111508	tail morphogenetic protein	173	18261	AEA36766.1	5e-113 (99%)	Staphylococcus phage GH15
Gp151	111523..111759	Ig-like domain	78	8101	AVX47510.1	1e-40 (92%)	Staphylococcus phage vB_SauM_0414_108
Gp152	111856..112116	hypothetical protein	86	10205	ARM69214.1	7e-57 (100%)	Staphylococcus phage vB_Sau_Clo6
Gp153	112120..112875	hypothetical protein	251	29132	ARM69215.1	1e-180 (100%)	Staphylococcus phage vB_Sau_Clo6
Gp154	112868..114118	metallophosphoesterase	416	47610	ARM69429.1	0.0 (100%)	Staphylococcus phage vB_Sau_S24
Gp155	114132..114500	membrane protein	122	14023	YP_009006809.1	4e-82 (100%)	Staphylococcus phage phiSA12
Gp156	114487..114798	hypothetical protein	103	11967	ARM69431.1	2e-70 (100%)	Staphylococcus phage vB_Sau_S24
Gp157	114864..115400	hypothetical protein	178	20826	ARM69219.1	5e-129 (100%)	Staphylococcus phage vB_Sau_Clo6
Gp158	115393..116160	hypothetical protein	255	30047	ARM69220.1	0.0 (100%)	Staphylococcus phage vB_Sau_Clo6
Gp159	116138..116584	hypothetical protein	148	17405	ANH50449.1	2e-130 (100%)	Staphylococcus phage pSco-10
Gp160	116584..117447	hypothetical protein	287	32298	VEV88618.1	0.0 (100%)	Staphylococcus phage Stab23
Gp161	117806..118537	hypothetical protein	243	28342	YP_007002283.1	9e-175 (100%)	Staphylococcus phage GH15
Gp162	118555..119013	hypothetical protein	152	17823	YP_007002284.1	2e-106 (100%)	Staphylococcus phage GH15
Gp163	119078..119521	hypothetical protein	147	17443	ARM69438.1	2e-99 (100%)	Staphylococcus phage vB_Sau_S24
Gp164	19538..120242	hypothetical protein	234	27570	ARM69439.1	6e-161 (100%)	Staphylococcus phage vB_Sau_S24
Gp165	120305..120703	hypothetical protein	132	15381	ARM69227.1	5e-79 (100%)	Staphylococcus phage vB_Sau_Clo6
Gp166	120851..121096	hypothetical protein	81	9504	ARM69228.1	2e-48 (97%)	Staphylococcus phage vB_Sau_Clo6
Gp167	121166..121342	hypothetical protein	58	7052	YP_007002290.1	1e-31 (100%)	Staphylococcus phage GH15
Gp168	121335..121583	putative membrane protein	82	9125	VEV88279.1	3e-48 (100%)	Staphylococcus phage Stab20
Gp169	121576..121809	hypothetical protein	77	8869	ARM69232.1	4e-47 (100%)	Staphylococcus phage vB_Sau_Clo6
Gp170	121889..122533	se 1,5-biphosphate carboxylase/oxygenase small	214	25071	ARM69446.1	1e-144 (100%)	Staphylococcus phage vB_Sau_S24
Gp171	122808..122984	hypothetical protein	58	6924	ARM69448.1	1e-32 (100%)	Staphylococcus phage vB_Sau_S24
Gp172	122977..123273	hypothetical protein	98	11449	YP_009098099.1	1e-61 (100%)	Staphylococcus phage MCE-2014
Gp173	123312..123503	membrane protein	63	7398	ACB89144.1	6e-32 (100%)	Staphylococcus phage A5W
Gp174	123515..123901	hypothetical protein	128	15095	YP_009195998.1	3e-64 (100%)	Staphylococcus phage philPLA-RODI
Gp175	123914..124261	hypothetical protein	115	13047	ANH50431.1	5e-74 (100%)	Staphylococcus phage pSco-10
Gp176	1246267..124539	membrane protein	90	9959	ARM69240.1	5e-53 (100%)	Staphylococcus phage vB_Sau_Clo6
Gp177	124599..124919	hypothetical protein	106	12690	VEV88288.1	1e-65 (97%)	Staphylococcus phage Stab20
Gp178	124942..126000	hypothetical protein	352	41190	YP_009098107.1	5e-70 (99%)	Staphylococcus phage MCE-2014
Gp179	125979..126356	hypothetical protein	125	14672	ARM69242.1	6e-71 (92%)	Staphylococcus phage vB_Sau_Clo6
Gp180	126356..126958	hypothetical protein	200	23377	YP_007002302.1	5e-145 (100%)	Staphylococcus phage GH15
Gp181	126978..127370	hypothetical protein	130	15125	AZB66577.1	4e-15 (100%)	Staphylococcus phage phiSP38-1
Gp182	127371..127559	hypothetical protein	62	7570	ANH50426.1	3e-30 (95%)	Staphylococcus phage pSco-10
Gp183	127885..128334	putative membrane protein	149	16749	ARM69244.1	5e-80 (91%)	Staphylococcus phage vB_Sau_Clo6
Gp184	128336..128626	hypothetical protein	97	11624	ARM69458.1	2e-60 (100%)	Staphylococcus phage vB_Sau_S24
Gp185	128646..128873	putative membrane protein	75	8216	YP_238658.1	8.6	Staphylococcus virus Twort
Gp186	128889..129176	hypothetical protein	95	10902	ARM69460.1	3e-45 (100%)	Staphylococcus phage vB_Sau_S24
Gp187	129178..129843	hypothetical protein	221	25004	ARM69461.1	1e-150 (100%)	Staphylococcus phage vB_Sau_S24
Gp188	129920..130225	hypothetical protein	101	11643	ARM69248.1	9e-63 (100%)	Staphylococcus phage vB_Sau_Clo6
Gp189	130225..130638	hypothetical protein	137	15305	ARM69249.1	1e-50 (100%)	Staphylococcus phage vB_Sau_Clo6
Gp190	130641..131165	metallophosphoesterase	174	20477	YP_007002312.1	3e-122 (100%)	Staphylococcus phage GH15
Gp191	131258..131437	putative membrane protein	59	6360	AUV57092.1	2e-30 (100%)	Staphylococcus phage vB_SauM_LM12
Gp192	131452..131715	hypothetical protein	87	10295	YP_009041416.1	8e-50 (98%)	Staphylococcus virus K
Gp193	131718..132035	hypothetical protein	105	12066	ARM69464.1	9e-67 (100%)	Staphylococcus phage vB_Sau_S24
Gp194	132036..132716	hypothetical protein	226	25789	ARM69036.1	2e-130 (100%)	Staphylococcus phage vB_Sau_CG
Gp195	132794..133018	hypothetical protein	74	8477	ARM69252.1	9e-48 (100%)	Staphylococcus phage vB_Sau_Clo6
Gp196	133034..133294	membrane protein	86	9708	BBC69640.1	1e-27 (97%)	Staphylococcus phage phiSA039
Gp197	13310..134218	ribose-phosphate pyrophosphokinase	302	34752	ARM69253.1	0.0 (100%)	Staphylococcus phage vB_Sau_Clo6
Gp198	134236..136674	nicotinamide phosphoribosyl transferase	812	93458	ARM69254.1	0.0 (100%)	Staphylococcus phage vB_Sau_Clo6
Gp199	136754..137008	hypothetical protein	84	9939	ARM69469.1	7e-47 (100%)	Staphylococcus phage vB_Sau_S24
Gp200	137031..137342	hypothetical protein	103	11707	ARM69256.1	1e-66 (100%)	Staphylococcus phage vB_Sau_Clo6
Gp201	137367..137801	hypothetical protein	144	16853	AVR55483.1	1e-77 (97%)	Staphylococcus phage phiSA_BS2
Gp202	137794..137938	hypothetical protein	48	5772	.....	.....	.....
Gp203	138187..138336	hypothetical protein	49	6062	AUV57107.1	1e-11 (100%)	Staphylococcus phage vB_SauM_LM12
Gp204	138365..138628	hypothetical protein	88	10778	ATP66760.1	.....	.....
Gp205	138660..138971	hypothetical protein	103	12128	ARM69266.1	2e-52 (100%)	Staphylococcus phage vB_Sau_Clo6
Gp206	138986..139333	hypothetical protein	115	13463	AVP40379.1	6e-59 (98%)	Staphylococcus phage phiSA_BS1
Gp207	139348..139827	hypothetical protein	159	18930	ANH50414.1	6e-22 (39%)	Staphylococcus phage pSco-10
Gp208	139901..140023	hypothetical protein	40	4852	ARM69258.1	3e-17 (92%)	Staphylococcus phage vB_Sau_Clo6
Gp209	140055..140228	hypothetical protein	57	7022	ANT44694.1	4e-24 (100%)	Staphylococcus phage vB_SscM-1
Gp210	140297..140494	hypothetical protein	66	7636	AXF38435.1	4e-15 (92%)	Staphylococcus phage Quidividi
Gp211	140773..140997	hypothetical protein	74	8632	YP_006561216.1	3e-32 (100%)	Staphylococcus virus IPLA7
Gp212	141043..141444	hypothetical protein	133	16128	AVP40385.1	3-87 (100%)	Staphylococcus phage phiSA_BS1
Gp213	141479..141814	hypothetical protein	111	12917	VEV88320.1	9e-69 (100%)	Staphylococcus phage Stab20
Gp214	141814..142221	hypothetical protein	135	15478	AVR55468.1	1e-71 (100%)	Staphylococcus phage phiSA_BS2
Gp215	142306..142581	hypothetical protein	91	10492	YP_009196021.1	2e-42 (100%)	Staphylococcus phage philPLA-RODI
Gp216	142600..142830	hypothetical protein	76	9027	....	....	.....
Gp217	142971..143378	hypothetical protein	136	16229	YP_009196025.1	1e-79 (97%)	Staphylococcus phage philPLA-RODI
Gp218	143412..143549	hypothetical protein	46	5679	....	.....	....
Gp001	143970..144149	hypothetical protein	59	7075	YP_008854130.1	3e-15 (96%)	Staphylococcus phage S25-4
Gp002	149269..149427	hypothetical protein	174	19562	AVP40463.1	4e-69 (98%)	Staphylococcus phage phiSA_BS1
Gp003	149501..149809	hypothetical protein	70	7958	....	.....	.....
Gp004	149972..150298	TreA	97	11137	ARM69483.1	2e-56 (100%)	Staphylococcus phage vB_Sau_S24
Gp005	150397..150714	membrane protein	61	7046	YP_008853952.1	8e-17 (98%)	Staphylococcus phage S25-4
Gp006	150794..151195	membrane protein	57	6631	ARM69272.1	5e-17 (100%)	Staphylococcus phage vB_Sau_Clo6
Gp007	151771..152025	hypothetical protein	98	11140	AXU40163.1	1e-50 (100%)	Staphylococcus phage VB_SavM_JYL01
Gp008	152239..152516	putative membrane protein	67	7957	.....	.....	......
Gp009	152609..153082	hypothetical protein	104	11887	ARM69275.1	2e-72 (100%)	Staphylococcus phage vB_Sau_Clo6
Gp010	153161..153325	hypothetical protein	99	11591	ARM69488.1	8e-63 (100%)	Staphylococcus phage vB_Sau_S24
Gp011	15338..1535601	TreC	95	10992	ARM69276.1	4e-51 (100%)	Staphylococcus phage vB_Sau_Clo6
Gp012	153605..153793	TreE	98	11398	ARM69278.1	3e-34 (100%)	Staphylococcus phage vB_Sau_Clo6
Gp013	153830..154075	TreF	75	8713	ARM69279.1	1e-44 (100%)	Staphylococcus phage vB_Sau_Clo6
Gp014	148413..148643	terminal repeat-encoded protein	76	9014	YP_009099458.1	3e-21 (100%)	Staphylococcus phage P108
Gp015	148727..148987	hypothetical protein	87	10195	YP_008853956.1	1e-37 (91%)	Staphylococcus phage S25-4
Gp016	148990..149238	terminal repeat-encoded protein	83	9443	BBC69667.1	2e-18 (100%)	Staphylococcus phage phiSA039
Gp017	149495..149776 c	hypothetical protein	94	11231	ASZ78147.1	1e-54 (100%)	Staphylococcus phage SA3
Gp018	150176..150490	TreI	104	12109	ARM69283.1	4e-60 (98%)	Staphylococcus phage vB_Sau_Clo6
Gp019	150597..151064	hypothetical protein	156	18824	AVP40358.1	4e-92 (100%)	Staphylococcus phage phiSA_BS1
Gp020	151141..151392	hypothetical protein	84	9973	AVR55650.1	5e-40 (100%)	Staphylococcus phage phiSA_BS2
Gp021	151923..152210	hypothetical protein	96	11308	....	.....	.....
Gp022	152550..152798	hypothetical protein	83	9758	AVP40364.1	2e-37 (100%)	Staphylococcus phage phiSA_BS1
Gp023	152872..153276	hypothetical protein	135	15710	YP_009097937.1	1e-83 (98%)	Staphylococcus phage MCE-2014
Gp024	153766..154092	hypothetical protein	105	11917	BBC69674.1	1e-49 (98%)	Staphylococcus phage phiSA039
Gp025	154170..154406	hypothetical protein	78	9068	ARM69294.1	6e-37 (100%)	Staphylococcus phage vB_Sau_Clo6
Gp026	154494..154976	terminal repeat-encoded protein	160	18554	YP_009195837.1	4e-76 (99%)	Staphylococcus phage philPLA-RODI
Gp027	155057..155245	hypothetical protein	62	7258	VEV88129.1	2e-33 (100%)	Staphylococcus phage Stab20
Gp028	155258..155527	TreT protein	89	10190	VEV88131.1	1e-48 (100%)	Staphylococcus phage Stab20
Gp029	155614..155844	TreU protein	76	9177	VEV88132.1	2e-36 (100%)	Staphylococcus phage Stab20

Gp143	108934..109176	hypothetical protein	80	9198	ARM69418.1	5e-51 (100%)	Staphylococcus phage vB_Sau_S24
Gp144	109193..109675	hypothetical protein	160	18932	ARM69206.1	3e-117 (100%)	Staphylococcus phage vB_Sau_Clo6
Gp145	109761..110933	hypothetical protein	390	43495	ARM69207.1	0.0 (100%)	Staphylococcus phage vB_Sau_Clo6
Gp146	110993..112249	repair recombinase	418	46734	ARM69208.1	0.0 (100%)	Staphylococcus phage vB_Sau_Clo6
Gp147	112253..112606	hypothetical protein	117	13352	ANH50461.1	1e-81 (100%)	Staphylococcus phage pSco-10
Gp148	112593..113255	RNA polymerase sigma factor	220	26594	ARM69210.1	7e-159 (100%)	Staphylococcus phage vB_Sau_Clo6
Gp149	113382..114014	hypothetical protein	210	23198	ARM69211.1	2e-151 (100%)	Staphylococcus phage vB_Sau_Clo6
Gp150	114036..114548	tail morphogenetic protein	170	17429	ARM69425.1	7e-114 (100%)	Staphylococcus phage vB_Sau_S24
Gp151	114563..114781	Ig-like domain	72	7399	YP_007002273.1	1e-37 (95%)	Staphylococcus phage GH15
Gp152	114877..115137	hypothetical protein	86	10232	ARM69214.1	2e-57 (100%)	Staphylococcus phage vB_Sau_Clo6
Gp153	115141..115896	hypothetical protein	251	29423	ARM69428.1	1e-178 (100%)	Staphylococcus phage vB_Sau_S24
Gp154	115889..117139	metallophosphoesterase	416	47594	ARM69429.1	0.0 (100%)	Staphylococcus phage vB_Sau_S24
Gp155	117153..117521	membrane protein	122	14068	ANH50453.1	2e-83 (100%)	Staphylococcus phage pSco-10
Gp156	117508..117819	hypothetical protein	103	11981	ARM69431.1	3e-70 (100%)	Staphylococcus phage vB_Sau_S24
Gp157	117883..118419	hypothetical protein	178	20830	ARM69219.1	2e-130 (100%)	Staphylococcus phage vB_Sau_Clo6
Gp158	118412..119179	hypothetical protein	255	30033	ANH50450.1	0.0 (100%)	Staphylococcus phage pSco-10
Gp159	119157..119603	hypothetical protein	148	17427	ARM69434.1	3e-105 (100%)	Staphylococcus phage vB_Sau_S24
Gp160	119603..120466	hypothetical protein	287	32318	ARM69222.1	0.0 (100%)	Staphylococcus phage vB_Sau_Clo6
Gp161	120825..121556	hypothetical protein	243	28351	ARQ96133.1	1e-175 (100%)	Staphylococcus phage qdsa002
Gp162	121574..122032	hypothetical protein	152	17907	ARM69437.1	2e-108 (100%)	Staphylococcus phage vB_Sau_S24
Gp163	122097..122540	hypothetical protein	147	17398	ARM69438.1	1e-100 (100%)	Staphylococcus phage vB_Sau_S24
Gp164	122557..123279	hypothetical protein	240	28131	ARM69439.1	2e-152 (100%)	Staphylococcus phage vB_Sau_S24
Gp165	123340..123738	putative membrane protein	132	15345	ARM69227.1	8e-81 (100%)	Staphylococcus phage vB_Sau_Clo6
Gp166	123886..124128	hypothetical protein	80	9393	ARM69441.1	4e-51 (100%)	Staphylococcus phage vB_Sau_S24
Gp167	124133..124690	putative membrane protein	185	21584	ARM69229.1	6e-129 (100%)	Staphylococcus phage vB_Sau_Clo6
Gp168	124726..124902	hypothetical protein	58	6935	ARM69443.1	6e-34 (100%)	Staphylococcus phage vB_Sau_S24
Gp169	124895..125143	putative membrane protein	82	9103	ARM69231.1	9e-51 (100%)	Staphylococcus phage vB_Sau_Clo6
Gp170	125136..125369	hypothetical protein	77	8916	ARM69445.1	2e-48 (100%)	Staphylococcus phage vB_Sau_S24
Gp171	125450..126094	ribulose 1, 5-biphosphate carboxylase/oxygenase small subunit	214	25158	ARM69446.1	6e-141 (100%)	Staphylococcus phage vB_Sau_S24
Gp172	126109..126357	hypothetical protein	82	8829	ARM69447.1	2e-45 (100%)	Staphylococcus phage vB_Sau_S24
Gp173	126369..126545	hypothetical protein	58	7009	YP_009098098.1	2e-33 (100%)	Staphylococcus phage MCE-2014
Gp174	126538..126834	hypothetical protein	98	11243	ARM69236.1	2e-60 (100%)	Staphylococcus phage vB_Sau_Clo6
Gp175	126882..127064	putative membrane protein	60	7068	ARM69237.1	3e-34 (100%)	Staphylococcus phage vB_Sau_Clo6
Gp176	127077..127445	hypothetical protein	122	14063	ARM69451.1	2e-84 (100%)	Staphylococcus phage vB_Sau_S24
Gp177	127458..127805	hypothetical protein	115	13026	ARM69239.1	1e-76 (100%)	Staphylococcus phage vB_Sau_Clo6
Gp178	127805..128083	putative membrane protein	92	10180	ARM69240.1	1e-56 (68%)	Staphylococcus phage vB_Sau_Clo6
Gp179	128153..128458	hypothetical protein	101	12163	YP_007002300.1	3e-70 (100%)	Staphylococcus phage GH15
Gp180	128473..128823	hypothetical protein	116	13666	YP_007112906.1	3e-77 (100%)	Staphylococcus phage JD007
Gp181	128823..129206	hypothetical protein	127	15075	ANH50427.1	2e-19 (100%)	Staphylococcus phage pSco-10
Gp182	129207..129386	hypothetical protein	59	7220	ANH50426.1	2e-31 (100%)	Staphylococcus phage pSco-10
Gp183	129612..130022	putative membrane protein	136	15168	ARM69244.1	2e-90 (100%)	Staphylococcus phage vB_Sau_Clo6
Gp184	130024..130317	hypothetical protein	97	11641	ARM69458.1	5e-63 (100%)	Staphylococcus phage vB_Sau_S24
Gp185	130334..130621	putative membrane protein	95	10554	ARM69459.1	2e-61 (100%)	Staphylococcus phage vB_Sau_S24
Gp186	130669..131334	hypothetical protein	221	25046	ARM69247.1	1e-153 (100%)	Staphylococcus phage vB_Sau_Clo6
Gp187	131411..131716	hypothetical protein	101	11646	ARM69248.1	1e-66 (100%)	Staphylococcus phage vB_Sau_Clo6
Gp188	131716..132123	hypothetical protein	135	15378	ARM69249.1	6e-85 (100%)	Staphylococcus phage vB_Sau_Clo6
Gp189	132126..132443	hypothetical protein	105	12152	ARM69464.1	3e-70 (100%)	Staphylococcus phage vB_Sau_S24
Gp190	132521..132724	hypothetical protein	60	7771	ARM69251.1	4e-37 (100%)	Staphylococcus phage vB_Sau_Clo6
Gp191	132758..132982	hypothetical protein	74	8477	ARM69252.1	1e-47 (100%)	Staphylococcus phage vB_Sau_Clo6
Gp192	132999..133907	ribose-phosphate pyrophosphokinase	302	35002	ARM69253.1	0.0 (100%)	Staphylococcus phage vB_Sau_Clo6
Gp193	133926..135395	Nicotinamide phosphoribosyltransferase	489	56102	ARM69468.1	0.0 (100%)	Staphylococcus phage vB_Sau_S24
Gp194	135476..135730	hypothetical protein	84	9866	ARM69469.1	1e-47 (100%)	Staphylococcus phage vB_Sau_S24
Gp195	135754..136065	hypothetical protein	103	11704	ARM69256.1	3e-69 (100%)	Staphylococcus phage vB_Sau_Clo6
Gp196	136144..136434	hypothetical protein	96	11568	ARM69471.1	6e-65 (100%)	Staphylococcus phage vB_Sau_S24
Gp197	136431..136544	hypothetical protein	37	4458	ARM69258.1	5e-17 (100%)	Staphylococcus phage vB_Sau_Clo6
Gp198	136575..136757	hypothetical protein	60	7226	....	....	.....
Gp199	136799..136960	hypothetical protein	53	6171	....	......	......
Gp200	137003..137284	hypothetical protein	93	10814	ARM69261.1	8e-57 (100%)	Staphylococcus phage vB_Sau_Clo6
Gp201	137342..139615	RNA ligase	757	89137	ARM69476.1	0.0 (100%)	Staphylococcus phage vB_Sau_S24
Gp202	139715..139984	hypothetical protein	89	10321	ARM69263.1	7e-59 (100%)	Staphylococcus phage vB_Sau_Clo6
Gp203	140013..140402	hypothetical protein	129	15203	ARM69264.1	3e-90 (100%)	Staphylococcus phage vB_Sau_Clo6
Gp204	140429..140578	hypothetical protein	49	5846	ARM69265.1	1e-26 (100%)	Staphylococcus phage vB_Sau_Clo6
Gp205	141030..142235	hypothetical protein	103	11972	ARM69266.1	1e-68 (100%)	Staphylococcus phage vB_Sau_Clo6
Gp206	141030..142235	hypothetical protein	401	47057	ANT44859.1	2e-91 (87%)	Staphylococcus phage vB_SscM-1
Gp001	553..1536	hypothetical protein	327	36796	ARM69482.1	1e-119 (79%)	Staphylococcus phage vB_Sau_S24
Gp002	1766..2059	hypothetical protein	97	11159	ARM69271.1	6e-58 (100%)	Staphylococcus phage vB_Sau_Clo6
Gp003	2056..2217	putative membrane protein	53	5951	ARM69484.1	2e-26 (98%)	Staphylococcus phage vB_Sau_S24
Gp004	2314..2499	putative membrane protein	61	7238	....	....	.....
Gp005	2515..2829	hypothetical protein	104	11954	ARM69275.1	1e-70 (100%)	Staphylococcus phage vB_Sau_Clo6
Gp006	2843..3136	hypothetical protein	97	11232	ARM69277.1	5e-55 (100%)	Staphylococcus phage vB_Sau_Clo6
Gp007	3140..3391	hypothetical protein	83	9746	ARM69279.1	2e-52 (100%)	Staphylococcus phage vB_Sau_Clo6
Gp008	3479..3727	hypothetical protein	82	9860	ARM69280.1	4e-37 (100%)	Staphylococcus phage vB_Sau_Clo6
Gp009	3740..3976	hypothetical protein	78	8862	ARM69493.1	2e-50 (100%)	Staphylococcus phage vB_Sau_S24
Gp010c	(4219..4551)c	hypothetical protein	110	13307	ARM69282.1	8e-61 (100%)	Staphylococcus phage vB_Sau_Clo6
Gp011	4862..5170	TreJ	102	11938	ARM69283.1	8e-64 (100%)	Staphylococcus phage vB_Sau_Clo6
Gp012	5362..5835	hypothetical protein	157	18855	ARM69285.1	3e-101 (98%)	Staphylococcus phage vB_Sau_Clo6
Gp013	5892..6137	hypothetical protein	81	9901	AVP40359.1	3e-36 (96%)	Staphylococcus phage phiSA_BS1
Gp014	6995..7153	TreN	52	5985	ARM69287.1	2e-27 (100%)	Staphylococcus phage vB_Sau_Clo6
Gp015	7227..7535	hypothetical protein	102	11944	ARM69500.1	2e-68 (100%)	Staphylococcus phage vB_Sau_Clo6
Gp016	7698..8024	TreP	108	12401	ARM69501.1	1e-71 (100%)	Staphylococcus phage vB_Sau_S24
Gp017	8123..8440	hypothetical protein	105	12181	BBC69674.1	8e-33 (100%)	Staphylococcus phage phiSA039
Gp018	8514..8921	hypothetical protein	135	15740	ARM69291.1	1e-84 (98%)	Staphylococcus phage vB_Sau_Clo6
Gp019	9497..9751	hypothetical protein	84	9730	ARM69292.1	3e-56 (100%)	Staphylococcus phage vB_Sau_Clo6
Gp020	9965..10243	hypothetical protein	92	10789	VEV88348.1	2e-51 (100%)	Staphylococcus phage Stab20
Gp021	10335..10808	hypothetical protein	157	18016	ARM69078.1	8e-87 (100%)	Staphylococcus phage vB_Sau_CG
Gp022	10887..11051	hypothetical protein	54	6181	ARM69297.1	1e-29 (100%)	Staphylococcus phage vB_Sau_Clo6
Gp023	11064..11327	hypothetical protein	87	10250	ARM69079.1	6e-55 (100%)	Staphylococcus phage vB_Sau_CG
Gp024	11331..11519	hypothetical protein	62	7173	VEV88129.1	2e-33 (100%)	Staphylococcus phage Stab20
Gp025	11532..11801	TreT	89	10112	ARM69081.1	6e-55 (100%)	Staphylococcus phage vB_Sau_CG
Gp026	11886..12107	TreU	73	8944	VEV88132.1	6e-45 (100%)	Staphylococcus phage Stab20
